# Supplementary material for: Homo/Hetero-Dimers of Aromatic Bisabolane Sesquiterpenoids with Neuroprotective Activity from the Fungus Aspergillus versicolor A18 from South China Sea
Source: Mar Drugs. 2022 May 13;20(5):322. doi: 10.3390/md20050322 (PMC9146609; doi:10.3390/md20050322)
Supplement: Supplementary file 1 [file marinedrugs-20-00322-s001.zip › marinedrugs-1714875-supplementary.pdf]

Supporting Information

Homo/Hetero-Dimers of Aromatic Bisabolane Sesquiterpenoids with Neuroprotective Activity from the Fungus *Aspergillus versicolor* A18 from South China Sea

Han-Zhuang Weng [1], Jun-Yu Zhu [1], Fang-Yu Yuan [1], Zhuo-Ya Tang [1], Xiao-Qing Tian[2], Ye Chen [1], Cheng-Qi Fan [2,*], Gui-Hua Tang [1,*] and Sheng Yin [1]

[1] School of Pharmaceutical Sciences, Sun Yat-sen University, Guangzhou, Guangdong 510006, China; wenghzh@mail2.sysu.edu.cn (H-Z. Weng); zhujy76@mail2.sysu.edu.cn (J-Y Zhu); yuanfy@mail2.sysu.edu.cn (F-Y. Yuan); tangzhy23@mail2.sysu.edu.cn (Z-Y. Tang); chenye8@mail.sysu.edu.cn (Y. Chen); yinsh2@mail.sysu.edu.cn (S. Yin);

[2] East China Sea Fisheries Research Institute, Chinese Academy of Fishery Sciences, Shanghai 200090, China; amytian0904@126.com (X.-Q. Tian)

* Correspondence: tanggh5@mail.sysu.edu.cn (G-H. Tang); fancq@ecsf.ac.cn (C-Q. Fan)

Table of Contents

Table S1. NMR data for compound **1** in $CDCl_3$ (δ in ppm) …….………………………………4
Table S2. NMR data for compound **2** in $CDCl_3$ (δ in ppm) …….………………………………5
Table S3. NMR data for compound **3** in $CDCl_3$ (δ in ppm) …….………………………………6
Table S4. NMR data for compound **4** in $CDCl_3$ (δ in ppm) …….………………………………7
Table S5. NMR data for compound **5** in $CDCl_3$ (δ in ppm) …….………………………………8
Table S6. NMR data for compounds **6** and **7** in $CDCl_3$ (δ in ppm) …….………………………9
Table S7. NMR data for compound **8** in $CDCl_3$ (δ in ppm) …….………………….....................10
Table S8. NMR data for compounds **9** and **10** in $CDCl_3$ (δ in ppm) …….……………………11
Table S9. NMR data for compounds **12** and **13** (δ in ppm) …….…………………………...12
Table S10. NMR data for compounds **14** and **15** (δ in ppm) …….……………………………13
Figure S1. ECD Spectra of Compounds **2–4** and **12**…….…………………….. …..……………13
Figure S2. ^{1}H NMR (500 MHz, $CDCl_3$) spectrum of compound **1a**……..…..………………14
Figure S3. ^{13}C NMR (125 MHz, $CDCl_3$) spectrum of compound **1a**……..……………………15
Figure S4. ^{1}H NMR (500 MHz, $CDCl_3$) spectrum of compound **1b**………..…..………………16
Figure S5. ^{13}C NMR (125 MHz, $CDCl_3$) spectrum of compound **1b**………..………………….17
Figure S6. HSQC (500 MHz, $CDCl_3$) spectrum of compound **1b**………..…..………………18
Figure S7. ^{1}H-^{1}H COSY (500 MHz, $CDCl_3$) spectrum of compound **1b**……….……………19
Figure S8. HMBC (500 MHz, $CDCl_3$) spectrum of compound **1b**…………..…...……………..20
Figure S9. NOESY (500 MHz, $CDCl_3$) spectrum of compound **1b**……………...……………...21
Figure S10. HR-ESI-MS (E+) spectrum of compound **1**………..………………………………22
Figure S11. HR-ESI-MS (E−) spectrum of compound **1**…..………………….…………………23
Figure S12. ^{1}H NMR (400 MHz, $CDCl_3$) spectrum of compound **2**…….……………………...24
Figure S13. ^{13}C NMR (100 MHz, $CDCl_3$) spectrum of compound **2**……………………………25
Figure S14. HSQC (400 MHz, $CDCl_3$) spectrum of compound **2**………………………………26
Figure S15. ^{1}H-^{1}H COSY (400 MHz, $CDCl_3$) spectrum of compound **2**……………………..27
Figure S16. HMBC (400 MHz, $CDCl_3$) spectrum of compound **2**………………….................28
Figure S17. HR-ESI-MS spectrum of compound **2**……………………………………………...29
Figure S18. ^{1}H NMR (500 MHz, $CDCl_3$) spectrum of compound **3**……………….................30
Figure S19. ^{13}C NMR (125 MHz, $CDCl_3$) spectrum of compound **3**……………………………31
Figure S20. HSQC (500 MHz, $CDCl_3$) spectrum of compound **3**……………………………….32
Figure S21. ^{1}H-^{1}H COSY (500 MHz, $CDCl_3$) spectrum of compound **3**……………………..33
Figure S22. HMBC (500 MHz, $CDCl_3$) spectrum of compound **3**………………..…………….34
Figure S23. HR-ESI-MS spectrum of compound **3**…………………………………..………….35
Figure S24. ^{1}H NMR (400 MHz, $CDCl_3$) spectrum of compound **4**…………………………….36
Figure S25. ^{13}C NMR (100 MHz, $CDCl_3$) spectrum of compound **4**……………………………37
Figure S26. HSQC (400 MHz, $CDCl_3$) spectrum of compound **4**……………………………….38
Figure S27. ^{1}H-^{1}H COSY (400 MHz, $CDCl_3$) spectrum of compound **4**……………….……….39

Figure S28. HMBC (400 MHz, $CDCl_3$) spectrum of compound **4**……………….……………40
Figure S29. ^{1}H NMR (400 MHz, $CDCl_3$) spectrum of compound **5**…………………………41
Figure S30. ^{13}C NMR (100 MHz, $CDCl_3$) spectrum of compound **5**…………………………42
Figure S31. HSQC (400 MHz, $CDCl_3$) spectrum of compound **5**………………………………43
Figure S32. ^{1}H-^{1}H COSY (400 MHz, $CDCl_3$) spectrum of compound **5**…………………44
Figure S33. HMBC (400 MHz, $CDCl_3$) spectrum of compound **5**………………………………45
Figure S34. ^{1}H NMR (400 MHz, $CDCl_3$) spectrum of compound **6**…………………………46
Figure S35. ^{13}C NMR (100 MHz, $CDCl_3$) spectrum of compound **6**…………………………47
Figure S36. ^{1}H NMR (400 MHz, $CDCl_3$) spectrum of compound **7**…………………………48
Figure S37. ^{13}C NMR (100 MHz, $CDCl_3$) spectrum of compound **7**…………………………49
Figure S38. ^{1}H NMR (400 MHz, $CDCl_3$) spectrum of compound **8**…………………………50
Figure S39. ^{13}C NMR (100 MHz, $CDCl_3$) spectrum of compound **8**…………………………51
Figure S40. HSQC (400 MHz, $CDCl_3$) spectrum of compound **8**………………………………52
Figure S41. ^{1}H-^{1}H COSY (400 MHz, $CDCl_3$) spectrum of compound **8**…………………53
Figure S42. HMBC (400 MHz, $CDCl_3$) spectrum of compound **8**………………………………54
Figure S43. ^{1}H NMR (500 MHz, $CDCl_3$) spectrum of compound **9**…………………………55
Figure S44. ^{13}C NMR (125 MHz, $CDCl_3$) spectrum of compound **9**…………………………56
Figure S45. ^{1}H NMR (400 MHz, $CDCl_3$) spectrum of compound **10**…………………………57
Figure S46. ^{13}C NMR (100 MHz, $CDCl_3$) spectrum of compound **10**………………………58
Figure S47. ^{1}H NMR (400 MHz, Acetone-d_6) spectrum of compound **11**……………………59
Figure S48. ^{13}C NMR (100 MHz, Acetone-d_6) spectrum of compound **11**…………………60
Figure S49. ^{1}H NMR (400 MHz, $CDCl_3$) spectrum of compound **12**…………………………61
Figure S50. ^{13}C NMR (100 MHz, $CDCl_3$) spectrum of compound **12**………………………62
Figure S51. ^{1}H NMR (500 MHz, Acetone-d_6) spectrum of compound **13**……………………63
Figure S52. ^{13}C NMR (125 MHz, Acetone-d_6) spectrum of compound **13**…………………64
Figure S53. ^{1}H NMR (500 MHz, Acetone-d_6) spectrum of compound **14**……………………65
Figure S54. ^{13}C NMR (125 MHz, Acetone-d_6) spectrum of compound **14**…………………66
Figure S55. ^{1}H NMR (400 MHz, $CDCl_3$) spectrum of compound **15**…………………………67
Figure S56. ^{13}C NMR (100 MHz, $CDCl_3$) spectrum of compound **15**………………………68
Figure S57. B3LYP/6-31G(d) optimized lowest energy conformers for compound **1a**……………………………………………………………………………………………69
Table S11. Energy (298.15 K) analysis for **1a**…………………………………………………69
Table S12. Calculated ECD data for **1a** in PCM (acetonitrile)……………………………………70

Table S1. NMR data for compound **1** in $CDCl_3$ (δ in ppm)

Position	δ_C, Type	δ_H (J in Hz)	HMBC correlations	^1H–^1H COSY correlations
1	129.9, C			
2	151.6, C			
3	117.4, CH	6.64, s	C-1, C-2, C-5, and C-15	
4	134.3, C			
5	122.4, CH	6.62, d (7.7)	C-1, C-3, C-6, and C-15	H-5/H-6
6	127.9, CH	6.93, d (7.7)	C-2, C-4, and C-7	
7	131.7, C			
8	132.0, CH	5.53, t (7.0)	C-1, C-9, C-10, and C-14	
9	26.5, CH_2	2.20, q (8.0)	C-7, C-8, C-10, and C-11	H-8/H-9,
10	38.7, CH_2	1.32, q (7.5)	C-8, C-9, C-11, C-12, and C-13	H-9/H-10,
11	27.9, CH	1.60, m	C-9, C-10, C-12, and C-13	H-10/H-11,
12	22.6, CH_3	0.91, d (6.6)	C-10, C-11, and C-13	H-11/H-12 and H-13
13	22.6, CH_3	0.91, d (6.6)	C-10, C-11, and C-12	
14	18.0, CH_3	1.94, s	C-1, C-7, and C-8	
15	40.3, CH_2	2.94, d (13.4)<br>3.31, d (13.4)	C-3, C-4, C-5, C-3′, C-4′, and C-5′	
1′	157.1, C			
2′	124.0, CH	6.10, s	C-4′, C-6′, and C-7′	
3′	197.6, C			
4′	81.8, C			
5′	118.9, C			
6′	42.0, CH_2	3.01, d (19.1)<br>3.14, d (19.0)	C-1′,C-2′, C-4′, C-5′, and C-7′	
7′	24.3, CH_3	2.10, s	C-1′, C-2′, and C-6′	
8′	132.2, C			
9′	102.4, CH	6.33, s	C-8′, C-10′, C-11′, C-13′, and C-14′	
10′	148.6, C			
11′	132.2, C			
12′	138.1, C			
13′	111.3, CH	6.26, s	C-9′, C-11′, C-12′, and C-14′	
14′	21.5, CH_3	2.23, s	C-8′, C-9′, and C-13′	
2-OH		5.51, s	C-1, C-2, and C-3	
4′-OH		3.52, s	C-15, C-3′, C-4′, and C-5′	
10′-OH		4.78, s		

Table S2. NMR data for compound **2** in $CDCl_3$ (δ in ppm)

Position	δ_C, Type	δ_H (*J* in Hz)	HMBC correlations	^1H–^1H COSY correlations
1	128.0, C			
2	156.1, C			
3	116.4, CH	6.92, s	C-1, C-2, C-5, and C-15	
4	137.3, C			
5	119.1, CH	6.89, d (7.9)	C-1, C-3, and C-15	H-5/H-6
6	127.7, CH	7.00, d (7.9)	C-2, C-4, and C-7	
7	82.9, C			
8	40.2, CH_2	1.82, m<br>1.93, m	C-1, C-7, C-9, C-10, and C-14	H-8/H-9,
9	21.8, CH_2	1.33, m	C-10 and C-11	H-9/H-10,
10	39.1, CH_2	1.14, m	C-8, C-9, C-11, C-12, and C-13	H-10/H-11,
11	27.9, CH	1.50, m	C-10, C-12, and C-13	H-11/H-12 and H-13
12	22.7, CH_3	0.83, d (6.6)	C-10, C-11, and C-13	
13	22.8, CH_3	0.84, d (6.6)	C-10, C-11, and C-12	
14	22.4, CH_3	1.58, s	C-1, C-7, and C-8	
15	66.2, CH_2	5.27, s	C-3, C-4, C-5, and C-15′	
1′	134.5, C			
2′	156.3, C			
3′	119.2, CH	7.56, s	C-1′, C-2′, C-4′, C-5′, and C-15′	
4′	130.7, C			
5′	120.8, CH	7.52, d (8.2)	C-1′, C-3′, and C-15′	H-5′/H-6′
6′	126.4, CH	7.05, d (8.2)	C-2′, C-4′, and C-7′	
7′	79.2, C			
8′	43.1, CH_2	1.81, m<br>1.90, m	C-1′, C-7′, C-9′, C-10′, and C-14′	H-8′/H-9′,
9′	21.8, CH_2	1.29, m	C-10′ and C-11′	H-9′/H-10′,
10′	39.3, CH_2	1.14, m	C-8′, C-9′, C-11′, C-12′, and C-13′	H-10′/H-11′,
11′	27.9, CH	1.50, m	C-10′, C-12′, and C-13′	H-11′/H-12′ and H-13′
12′	22.7, CH_3	0.83, d (6.6)	C-10′, C-11′, and C-13′	
13′	22.7, CH_3	0.83, d (6.6)	C-10′, C-11′, and C-12′	
14′	29.3, CH_3	1.66, s	C-1′, C-7′, and C-8′	
15′	166.3, C			
2-OH		8.88, s	C-1, C-2, and C-3	
2′-OH		9.30, s	C-2′	
7-OMe	50.6, CH_3	3.22, s	C-7	

Table S3. NMR data for compound **3** in $CDCl_3$ (δ in ppm)

**Position**	**δ_C, Type**	**δ_H (*J* in Hz)**	**HMBC correlations**	**^{1}H–^{1}H COSY correlations**
1	131.1, C			
2	152.2, C			
3	115.0, CH	6.98, s	C-1, C-2, C-5, and C-15	
4	136.2, C			
5	119.9, CH	6.93, d (7.8)	C-1, C-3, and C-15	H-5/H-6
6	128.6, CH	7.07, d (7.8)	C-1, C-2, C-4, and C-7	
7	131.6, C			
8	132.3, CH	5.56, t (7.1)	C-1, C-9, and C-14	
9	26.5, CH_2	2.22, q (7.5)	C-7, C-8, C-10, and C-11	H-8/H-9,
10	38.7, CH_2	1.33, m	C-8, C-9, C-11, C-12, and C-13	H-9/H-10,
11	27.9, CH	1.60, m	C-10, C-12, and C-13	H-10/H-11,
12	22.7, CH_3	0.93, d (6.6)	C-10, C-11, and C-13	H-11/H-12 and H-13
13	22.7, CH_3	0.93, d (6.6)	C-10, C-11, and C-12	
14	18.0, CH_3	1.98, s	C-1, C-7, and C-8	
15	66.4, CH_2	5.27, s	C-3, C-4, C-5, and C-15′	
1′	134.5, C			
2′	156.3, C			
3′	119.0, CH	7.55, s	C-1′, C-2′, C-5′, and C-15′	
4′	130.7, C			
5′	120.8, CH	7.52, d (8.2)	C-1′, C-3′, and C-15′	H-5′/H-6′
6′	126.4, CH	7.04, d (8.1)	C-2′, C-4′, and C-7′	
7′	79.2, C			
8′	43.1, CH_2	1.79, m<br>1.91, m	C-1′, C-7′, and C-14′	
9′	21.8, CH_2	1.28, m	C-8′ and C-10′	H-8′/H-9′,
10′	39.1, CH_2	1.14, m	C-11′, C-12′, and C-13′	H-9′/H-10′,
11′	27.9, CH	1.49, m	C-10′, C-12′, and C-13′	H-10′/H-11′,
12′	22.7, CH_3	0.82, d (6.6)	C-10′, C-11′, and C-13′	H-11′/H-12′ and H-13′
13′	22.7, CH_3	0.83, d (6.6)	C-10′, C-11′, and C-12′	
14′	29.3, CH_3	1.66, s	C-1′, C-7′, and C-8′	
15′	166.3, C			
2′-OH		9.30, s		

Table S4. NMR data for compound **4** in $CDCl_3$ (δ in ppm)

Position	δ_C, Type	δ_H (*J* in Hz)	HMBC correlations	^1H–^1H COSY correlations
1	129.7, C			
2	156.2, C			
3	117.0, CH	6.90, s	C-1, C-2, C-5, and C-15	
4	136.9, C			
5	118.9, CH	6.86, d (7.9)	C-1, C-3, and C-15	H-5/H-6
6	126.5, CH	6.98, d (7.9)	C-2 and C-4	
7	78.9, C			
8	43.0, CH_2	1.80, m<br>1.89, m	C-1, C-7, C-9, C-10, and C-14	H-8/H-9,<br>H-9/H-10,<br>H-10/H-11,<br>H-11/H-12 and H-13
9	21.8, CH_2	1.29, m	C-7, C-8, C-10, and C-11	
10	39.2, CH_2	1.15, m	C-8, C-9, C-11, C-12, and C-13	
11	27.9, CH	1.49, m	C-9, C-10, C-12, and C-13	
12	22.7, CH_3	0.82, d (6.6)	C-10, C-11, and C-13	
13	22.6, CH_3	0.84, d (6.6)	C-10, C-11, and C-12	
14	29.1, CH_3	1.64, s	C-1, C-7, and C-8	
15	66.2, CH_2	5.24, s	C-3, C-4, C-5, and C-15′	
1′	134.8, C			
2′	156.1, C			
3′	118.9, CH	7.50, s	C-1′, C-2′, C-4′, C-5′, and C-15′	
4′	130.6, C			
5′	120.9, CH	7.50, d (8.2)	C-1′, C-3′, and C-15′	H-5′/H-6′
6′	126.4, CH	6.98, d (8.2)	C-2′, C-4′, and C-7′	
7′	79.1, C			
8′	43.0, CH_2	1.80, m<br>1.89, m	C-1′, C-7′, C-9′, C-10′, and C-14′	
9′	21.8, CH_2	1.29, m	C-7′, C-8′, C-10′, and C-11′	H-8′/H-9′,<br>H-9′/H-10′,<br>H-10′/H-11′,<br>H-11′/H-12′ and H-13′
10′	39.1, CH_2	1.15, m	C-8′, C-9′, C-11′, C-12′, and C-13′	
11′	27.9, CH	1.49, m	C-9′, C-10′, C-12′, and C-13′	
12′	22.7, CH_3	0.81, d (6.6)	C-10′, C-11′, and C-13′	
13′	22.7, CH_3	0.82, d (6.6)	C-10′, C-11′, and C-12′	
14′	29.1, CH_3	1.65, s	C-1′, C-7′, and C-8′	
15′	166.4, C			

Peniciaculin B (**4**): colorless oil; $[\alpha]_D^{20}$= + 11.00 (c 0.1, MeCN); UV (MeCN): λ_{max} (logε) 223 (3.08), 246 (2.76), 286 (1.19) nm; ECD (MeCN): λ_{ext} (Δε) 191 (+7.76), 209 (–5.61), 230 (+1.61), 282 (+2.52) nm.

Table S5. NMR data for compound **5** in $CDCl_3$ (δ in ppm)

Position	δ_C, Type	δ_H (J in Hz)	HMBC correlations	^{1}H–^{1}H COSY correlations
1	125.7, C			
2	151.7, C			
3	114.9, CH	6.81, s	C-1, C-2, C-5, and C-15	
4	140.7, C			
5	120.7, CH	6.79, d (7.5)	C-1, C-3, and C-15	H-5/H-6
6	128.7, CH	6.89, d (7.5)	C-2 and C-4	
7	131.1, C			
8	131.9, CH	5.66, td (1.2, 7.2)	C-1, C-7, C-9, C-10, and C-14	H-8/H-9, H-9/H-10, H-10/H-11, H-11/H-12 and H-13
9	27.2, CH_2	1.80, dd (7.2, 15.2)	C-7, C-8, C-10, and C-11	
10	38.9, CH_2	1.17, dd (7.0, 15.2)	C-8, C-9, C-11, C-12, and C-13	
11	27.5, CH	1.45, m	C-9, C-10, C-12, and C-13	
12	22.6, CH_3	0.77, d (6.6)	C-10, C-11, and C-13	
13	22.6, CH_3	0.77, d (6.6)	C-10, C-11, and C-12	
14	25.4, CH_3	1.93, s	C-1, C-7, C-8, and C-10	
15	29.1, CH_2	3.92, s	C-3, C-4, C-5, C-3′, and C-4′	
1′	138.3, C			
2′	112.5, CH	6.45, s	C-3′, C-4′, C-6′, and C-7′	
3′	155.3, C			
4′	116.4, C			
5′	155.2, C			
6′	113.3, CH	6.38, s	C-2′, C-3′, C-4′, and C-7′	
7′	21.3, CH_3	2.22, s	C-1′, C-2′, and C-6′	
8′	141.0, C			
9′	111.2, CH	6.34, s	C-10′, C-11′, C-13′, and C-14′	
10′	159.1, C			
11′	102.5, CH	6.21, t (2.0)	C-10′, and C-12′	
12′	156.7, C			
13′	110.6, CH	6.34, s	C-9′, C-11′, C-12′, and C-14′	
14′	21.6, CH_3	2.24, s	C-8′, C-9′, and C-13′	

Table S6. NMR data for compounds **6** and **7** in $CDCl_3$ (δ in ppm)

Position	6		7	
	δ_C, Type	δ_H (J in Hz)	δ_C, Type	δ_H (J in Hz)
1	125.1, C		128.6, C	
2	151.3, C		151.6, C	
3	114.5, CH	6.67, s	115.0, CH	6.66, s
4	140.4, C		140.4, C	
5	120.7, CH	6.71, d (7.4)	120.5, CH	6.67, d (7.7)
6	128.3, CH	6.87, d (7.4)	128.1, CH	6.92, d (7.7)
7	131.2, C		132.0, C	
8	131.8, CH	5.65, t (6.4)	131.7, CH	5.51, t (6.6)
9	27.2, CH_2	1.80, m	26.5, CH_2	1.74, m
10	38.9, CH_2	1.17, m	38.8, CH_2	1.31, dd (7.2, 15.2)
11	27.5, CH	1.44, m	27.9, CH	1.59, m
12	22.5, CH_3	0.76, d (6.1)	22.6, CH_3	0.91, d (6.6)
13	22.5, CH_3	0.76, d (6.1)	22.6, CH_3	0.91, d (6.6)
14	25.4, CH_3	1.94, s	18.0, CH_3	1.94, s
15	31.5, CH_2	3.90, s	31.4, CH_2	3.88, s
1′	141.0, C		141.0, C	
2′	123.0, C		122.9, C	
3′	155.8, C		155.7, C	
4′	104.7, CH	6.29, s	104.8, CH	6.29, s
5′	154.5, C		154.5, C	
6′	113.1, CH	6.46, s	113.4, CH	6.46, s
7′	20.2, CH_3	2.22, s	21.3, CH_3	2.22, s
8′	141.5, C		141.1, C	
9′	111.5, CH	6.33, s	111.4, CH	6.32, s
10′	158.8, C		158.8, C	
11′	102.7, CH	6.18, s	102.6, CH	6.17, s
12′	156.6, C		156.7, C	
13′	110.8, CH	6.33, s	110.7, CH	6.32, s
14′	21.6, CH_3	2.22, s	21.6, CH_3	2.22, s

Table S7. NMR data for compound **8** in $CDCl_3$ (δ in ppm)

Position	δ_C, Type	δ_H (J in Hz)	HMBC correlations	^1H–^1H COSY correlations
1	125.8, C			
2	155.9, C			
3	116.5, CH	6.64, s	C-2, C-5, and C-15	
4	141.2, C			
5	119.5, CH	6.65, d (7.8)	C-1, C-3, and C-15	H-5/H-6
6	127.6, CH	6.89, d (7.8)	C-2, C-4, and C-7	
7	82.9, C			
8	40.2, CH_2	1.78, m	C-1, C-9, and C-14	
9	21.7, CH_2	1.13, m<br>1.32, m	C-7, C-8, C-10, and C-11	H-8/H-9,<br>H-9/H-10,<br>H-10/H-11,<br>H-11/H-12 and H-13
10	39.2, CH_2	1.11, m	C-8, C-9, C-11, C-12, and C-13	
11	27.9, CH	1.48, m	C-9, C-10, C-12, and C-13	
12	22.6, CH_3	0.82, d (6.6)	C-10, C-11, and C-13	
13	22.7, CH_3	0.84, d (6.6)	C-10, C-11, and C-12	
14	22.4, CH_3	1.55, s	C-6, C-7, and C-8	
15	31.2, CH_2	3.94, s	C-3, C-4, C-5, C-1′, C-2′, and C-3′	
1′	139.9, C			
2′	120.4, C			
3′	155.1, C			
4′	104.5, CH	6.36, s	C-1′, C-2′, C-4′, and C-5′	
5′	156.0, C			
6′	113.4, CH	6.45, s	C-1′, C-2′, C-3′, C-4′, and C-7′	
7′	20.3, CH_3	2.22, s	C-1′, C-2′, and C-6′	
8′	141.0, C			
9′	111.2, CH	6.40, s	C-10′, C-11′, C-12′, and C-14′	
10′	156.9, C			
11′	103.4, CH	6.30, s	C-9′, C-10′, C-12′, C-13′	
12′	158.4, C			
13′	112.0, CH	6.42, s	C-9′, C-11′, C-12′, and C-14′,	
14′	21.6, CH_3	2.27, s	C-8′, C-9′, and C-13′	
7-OMe	50.5, CH_3	3.19, s	C-7	
2-OH		8.79, s	C-1, C-2, and C-3	

Expansol A (**8**): colorless oil; $[\alpha]_D^{20}$= + 3.67 (c 0.3, MeCN); UV (MeCN): λ_{max} (logε) 197 (3.87), 280 (0.51) nm.

Table S8. NMR data for compounds **9** and **10** in $CDCl_3$ (δ in ppm)

Position	**9**		**10**	
	δ_C, Type	δ_H (*J* in Hz)	δ_C, Type	δ_H (*J* in Hz)
1	129.8, C		129.3, C	
2	157.0, C		157.9, C	
3	117.5, CH	6.91, d (1.5)	116.9, CH	6.87, s
4	136.2, C		140.8, C	
5	119.3, CH	6.86, dd (1.5, 8.0)	118.7, CH	6.87, d (7.8)
6	126.6, CH	7.00, d (8.0)	126.6, CH	6.98, d (7.8)
7	79.1, C		79.1, C	
8	43.0, CH_2	1.80, m<br>1.91, m	43.0, CH_2	1.79, m<br>1.88, m
9	21.8, CH_2	1.29, m	21.8, CH_2	1.28, m
10	39.1, CH_2	1.15, m	39.2, CH_2	1.15, m
11	27.9, CH	1.50, m	27.9, CH	1.50, m
12	22.7, CH_3	0.83, d (6.6)	22.7, CH_3	0.83, d (6.5)
13	22.7, CH_3	0.83, d (6.6)	22.7, CH_3	0.83, d (6.5)
14	29.2, CH_3	1.66, s	29.1, CH_3	1.64, s
15	66.7, CH_2	5.31, s	69.6, CH_2	4.94, s
1′	143.9, C		137.8, C	
2′	ND [a]		112.6, CH	
3′	165.4, C		158.2, C	
4′	103.4, CH	6.34, d (2.3)	103.4, CH	6.24, s
5′	162.5, C		156.0, C	
6′	113.1, CH	6.36, d (2.3)	112.2, CH	6.43, s
7′	24.8, CH_3	2.51, s	21.8, CH_3	2.46, s
8′	141.4, C		141.0, C	
9′	112.7, CH	6.49, s	111.3, CH	6.54, s
10′	156.1, C		159.7, C	
11′	105.2, CH	6.36, d (2.6)	103.2, CH	6.43, s
12′	156.5, C		156.7, C	
13′	113.6, CH	6.44, s	111.4, CH	6.43, s
14′	21.6, CH_3	2.28, s	21.6, CH_3	2.28, s
15′	171.5, C			
2-OH		9.25, s		9.30, s
3′-OH		11.65, s		

[a] ND: No detected.

Aspertenol A (**9**): colorless oil; UV (MeCN): λ_{max} (log ε) 200 (2.78), 243 (0.35) nm, 264 (0.53) nm; ECD (MeCN): λ_{ext} (Δε) 205 (–1.68), 230 (+0.60), 280 (+0.74) nm.

Peniciaculin A (**10**): colorless oil; $[\alpha]_D^{20}$= + 5.00 (c 0.3, MeCN); UV (MeCN): λ_{max} (log ε) 201 (2.29), 278 (0.20) nm; ECD (MeCN): λ_{ext} (Δε) 224 (–0.63), 232 (+0.87), 279 (+2.23) nm.

Table S9. NMR data for compounds **11–13** (δ in ppm)

Position	**11** [a]		**12** [b]		**13** [a]	
	δ_C, Type	δ_H (J in Hz)	δ_C, Type	δ_H (J in Hz)	δ_C, Type	δ_H (J in Hz)
1	134.1, C		137.1, C		129.1, C	
2	154.6, C		157.0, C		154.9, C	
3	114.5, CH	6.83, s	119.1, CH	7.34, s	109.4, CH	7.38, s
4	143.4, C		136.1, C		139.2, C	
5	118.3, CH	6.73, d (7.6)	120.5, CH	7.15, d (7.4)	121.8, CH	7.17, d (7.8)
6	129.6, CH	6.85, d (7.6)	127.1, CH	7.35, d (7.4)	119.0, C	7.38, d (7.8)
7	128.1, C		79.4, C		110.0, C	
8	130.1, CH	5.41, t (7.2)	43.1, CH_2	1.82, m<br>1.93, m	155.2, C	
9	27.8, CH_2	1.79, dd (7.4,15.0)	21.7, CH_2	1.33, m	24.7, CH_2	2.75, t (7.6)
10	39.7, CH_2	1.13, dd (7.4,15.0)	39.1, CH_2	1.17, m	37.9, CH_2	1.58, m
11	28.2, CH	1.41, m	27.9, CH	1.49, m	28.3, CH	1.58, m
12	22.8, CH_3	0.70, d (6.6)	22.6, CH_3	0.83, d (6.6)	22.6, CH_3	0.94, d (6.4)
13	22.8, CH_3	0.70, d (6.6)	22.7, CH_3	0.83, d (6.6)	22.6, CH_3	0.94, d (6.4)
14	25.0, CH_3	1.88, s	29.3, CH_3	1.69, s	7.9, CH_3	2.15, s
15	64.5, CH_2	4.47, s	192.1, CH	9.92, s	64.9, CH_2	4.69, d (5.4)

[a] Measured in acetone-d_6.

[b] Measured in $CDCl_3$.

Table S10. NMR data for compounds **14** and **15** (δ in ppm)

Position	**14** [a]		**15** [b]	
	δ_C, Type	δ_H (J in Hz)	δ_C, Type	δ_H (J in Hz)
1	141.2, C		141.1, C	
2	112.5, CH	6.29, s	112.2, CH	6.40, s
3	159.0, C		158.1, C	
4	102.0, CH	6.25, t (2.0)	103.6, CH	6.29, s
5	159.1, C		156.7, C	
6	111.4, CH	6.43, s	111.4, CH	6.40, s
7	21.5, CH_3	2.25, s	21.6, CH_3	2.25, s
8	141.2, C		141.1, C	
9	112.0, CH	6.51, s	112.2, CH	6.40, s
10	159.4, C		158.1, C	
11	104.0, CH	6.35, s	103.6, CH	6.29, s
12	161.8, C		156.7, C	
13	110.3, CH	6.39, s	111.4, CH	6.40, s
14	21.4, CH_3	2.21, s	21.6, CH_3	2.25, s
12-OMe	55.5, CH_3	3.74, s		

[a] Measured in acetone-d_6.

[b] Measured in $CDCl_3$.

Figure S1. ECD Spectra of Compounds **2–4** and **12**

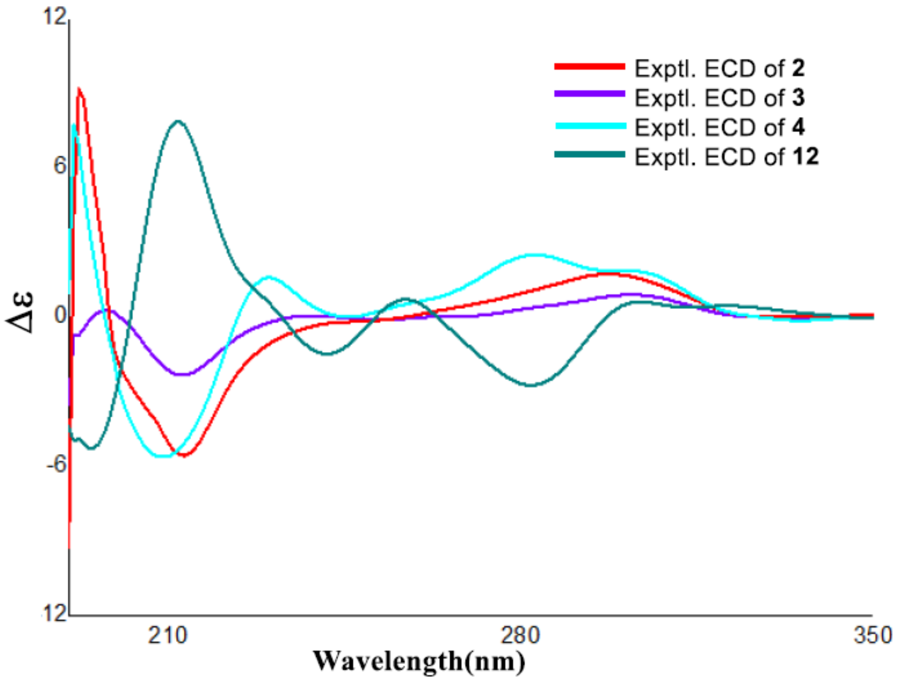

Figure S2. ¹H NMR (500 MHz, $CDCl_3$) Spectrum of Compound **1a**

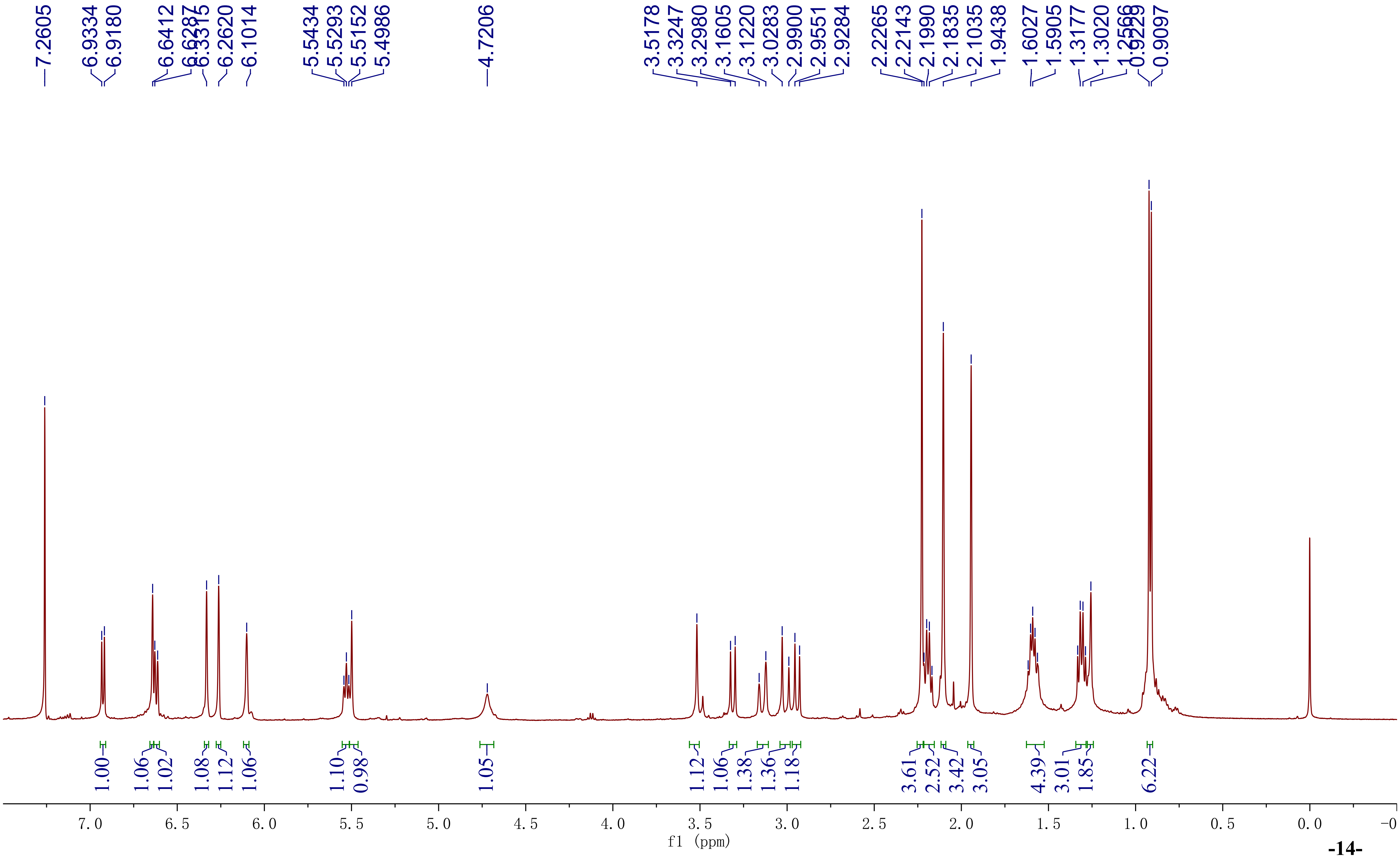

Figure S3. ^{13}C NMR (125 MHz, $CDCl_3$) Spectrum of Compound **1a**

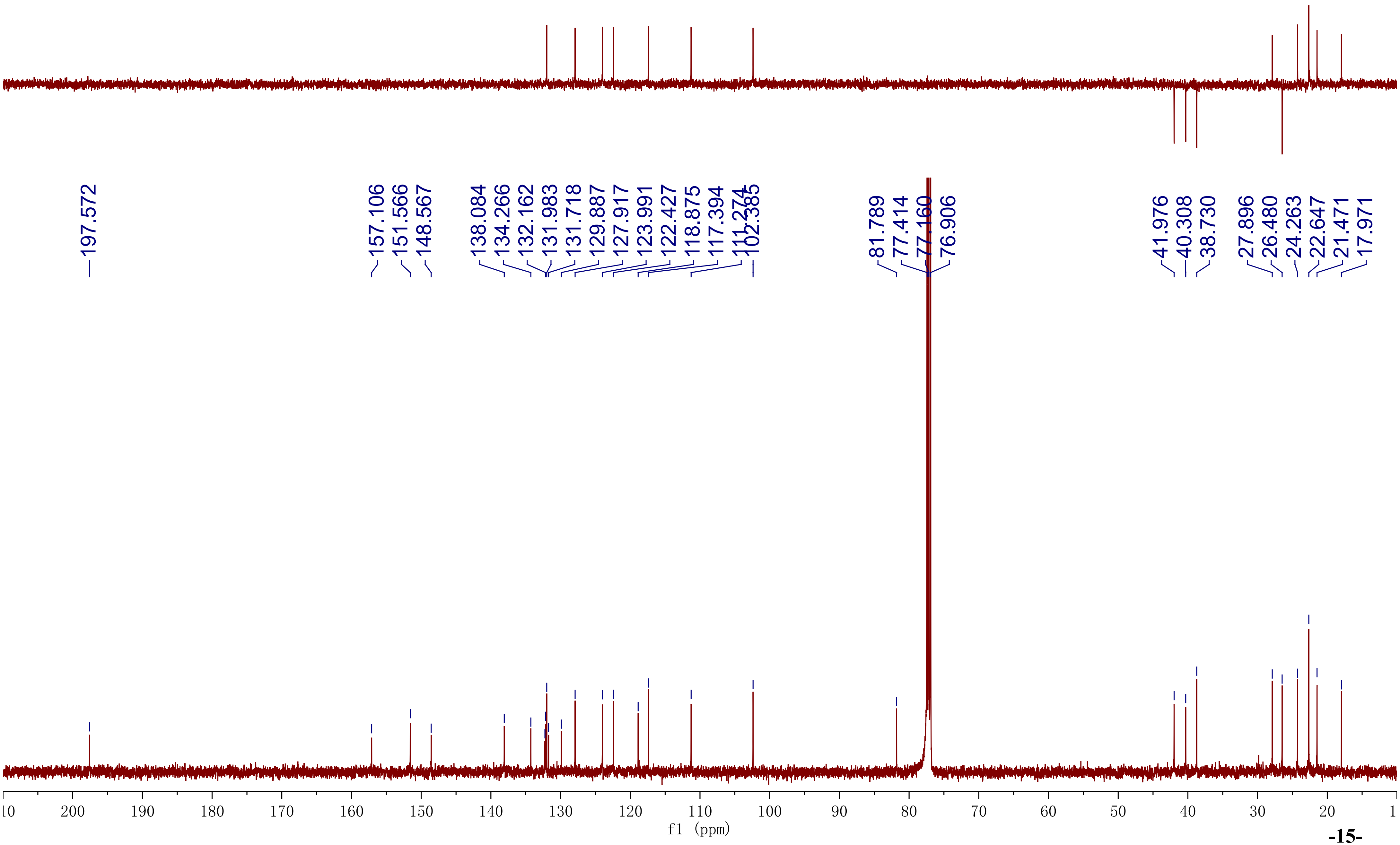

Figure S4. ^{1}H NMR (500 MHz, $CDCl_3$) Spectrum of Compound **1b**

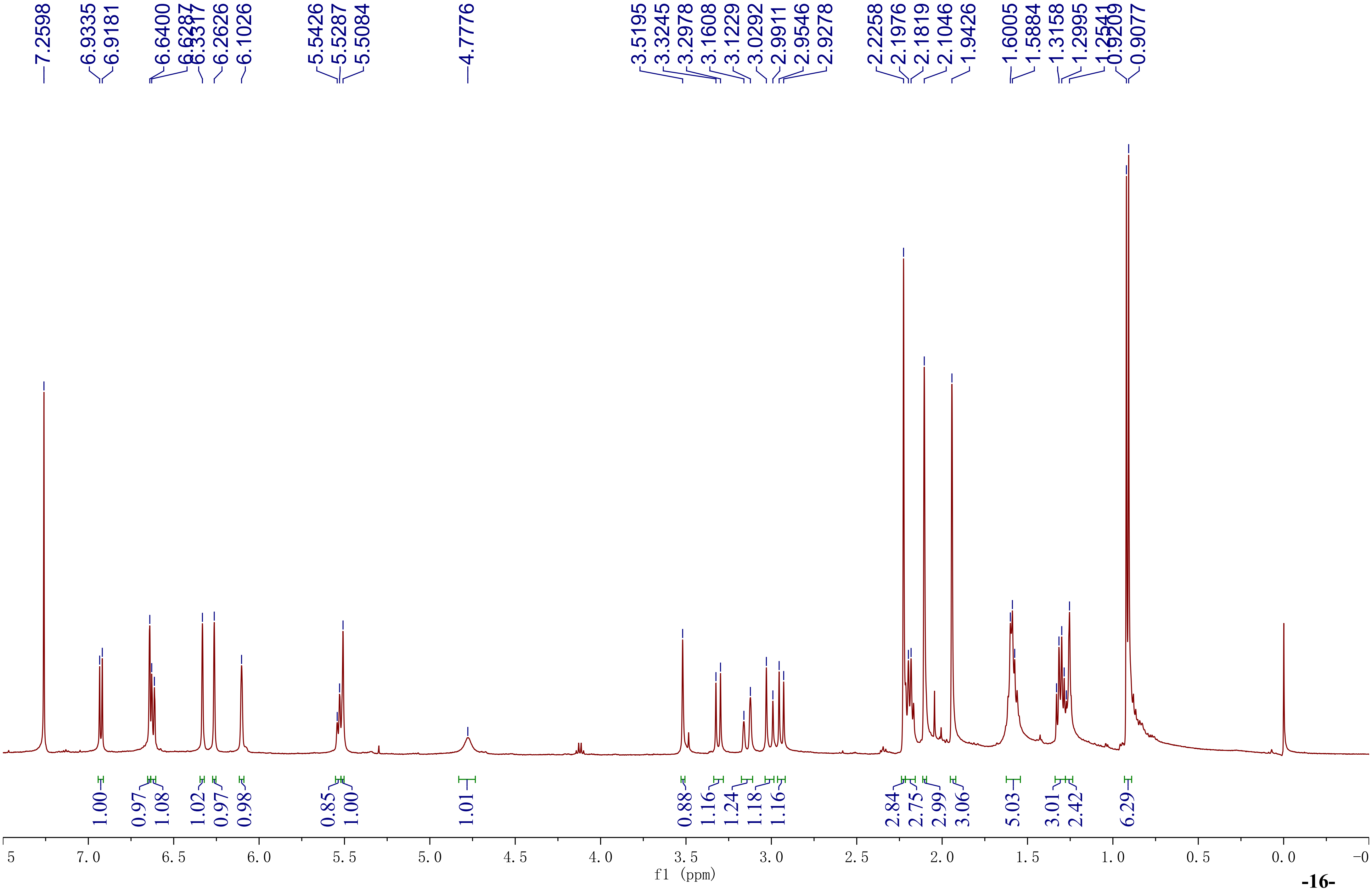

Figure S5. ^{13}C NMR (125 MHz, $CDCl_3$) Spectrum of Compound **1b**

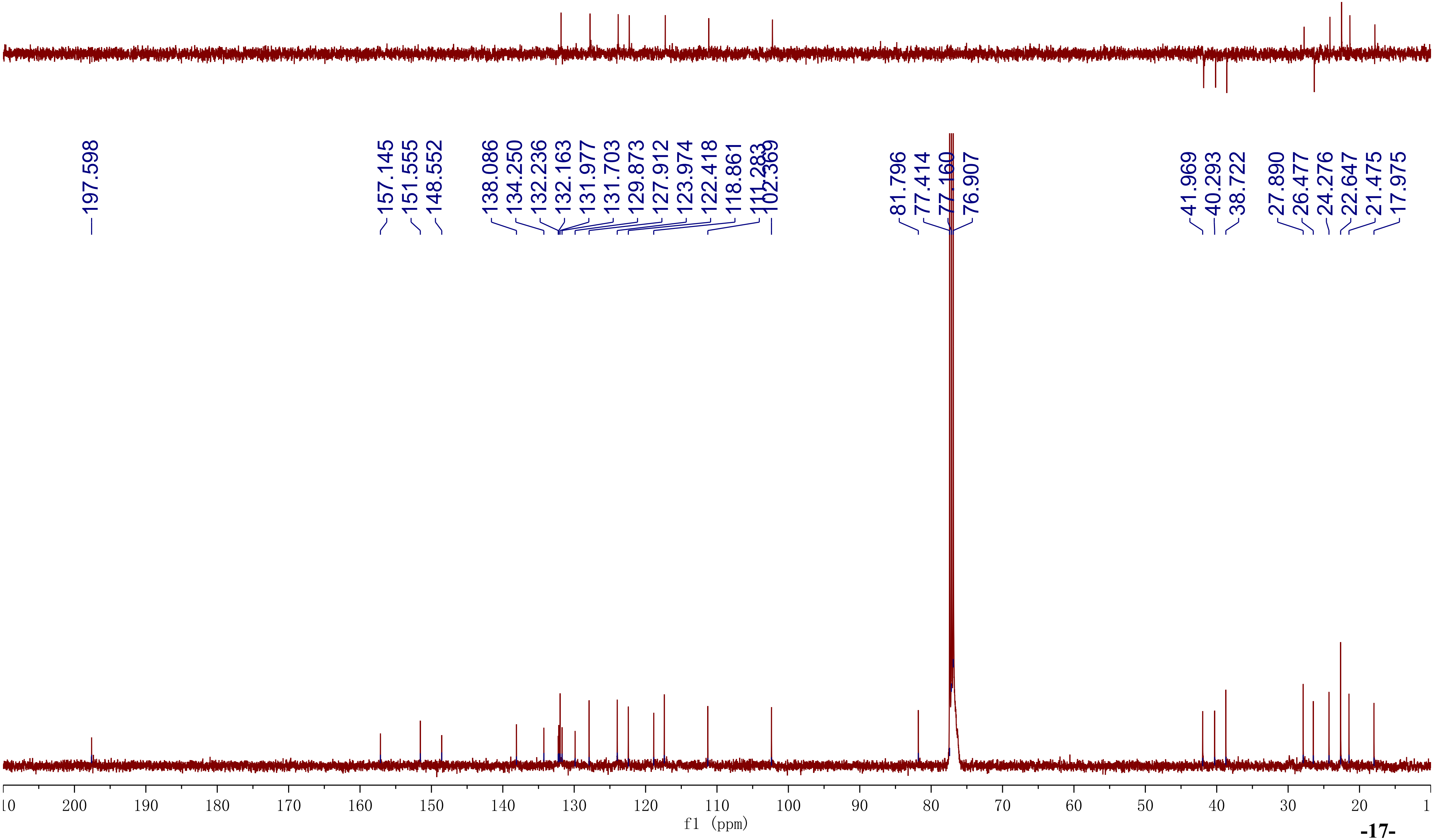

Figure S6. HSQC (500 MHz, $CDCl_3$) Spectrum of Compound **1b**

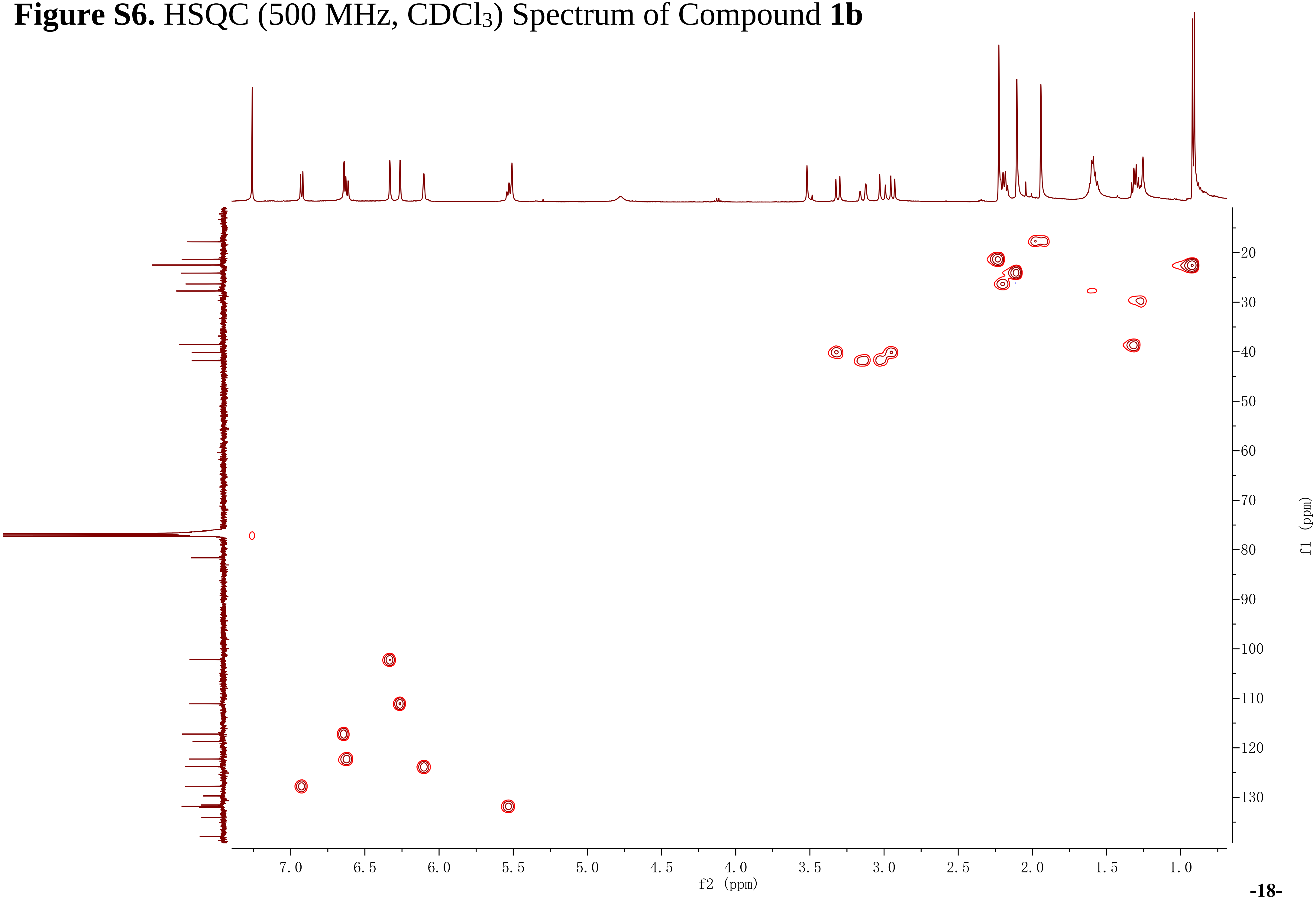

Figure S7. ^{1}H-^{1}H COSY (500 MHz, $CDCl_3$) Spectrum of Compound **1b**

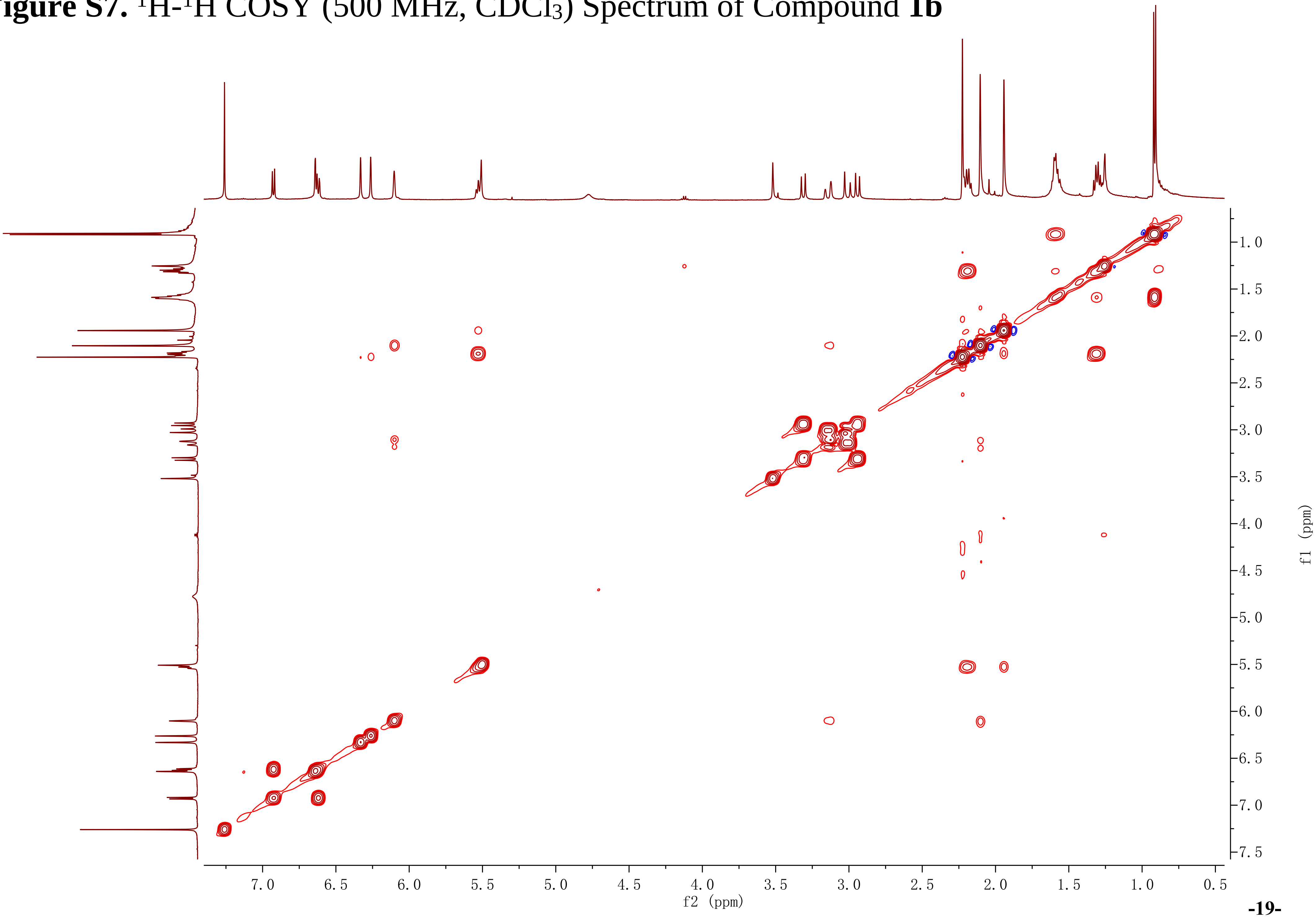

Figure S8. HMBC (500 MHz, $CDCl_3$) Spectrum of Compound **1b**

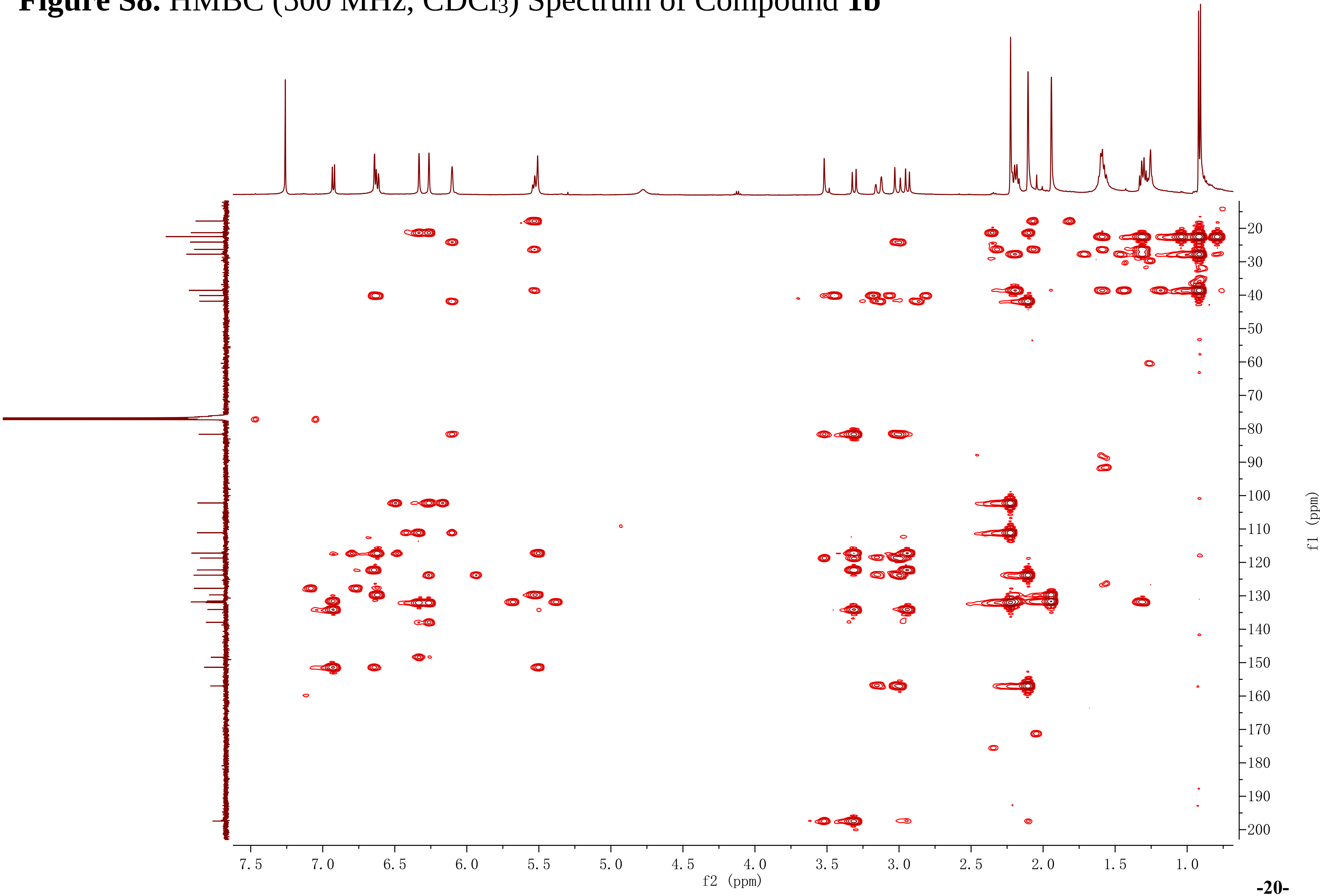

Figure S9. NOESY (500 MHz, $CDCl_3$) Spectrum of Compound **1b**

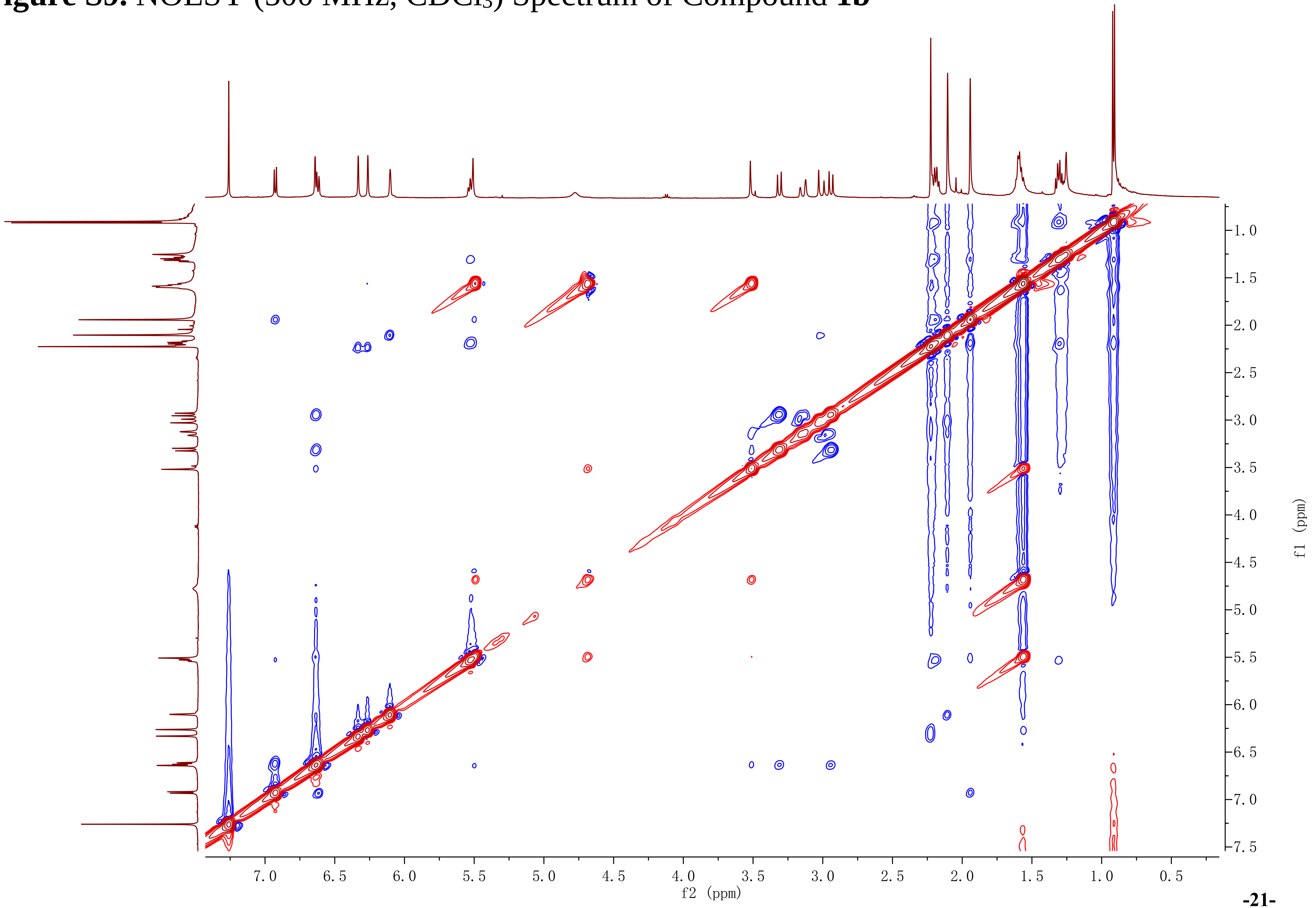

Figure S10. HR-ESI-MS (E+) Spectrum of Compound **1**

Elmt	Val.	Min	Max
H	1	30	38
B	3	0	0
C	4	29	29
N	3	0	0

Elmt	Val.	Min	Max
O	2	6	6
F	1	0	0
S	2	0	0
Cl	1	0	0

Elmt	Val.	Min	Max
Br	1	0	0
I	3	0	0

Use Adduct
H
Na
K
NH4

Error Margin (mDa):	50.0	DBE Range:	-100.0 - 200.0	Electron Ions:	both
HC Ratio:	0.0 - 100.0	Apply N Rule:	no	Use MSn Info:	no
Max Isotopes:	all	Isotope RI (%):	1.00	Isotope Res:	10000
MSn Iso RI (%):	75.00	MSn Logic Mode:	OR	Max Results:	1000

Event#: 1 MS(E+) Ret. Time : 0.983 Scan# : 147

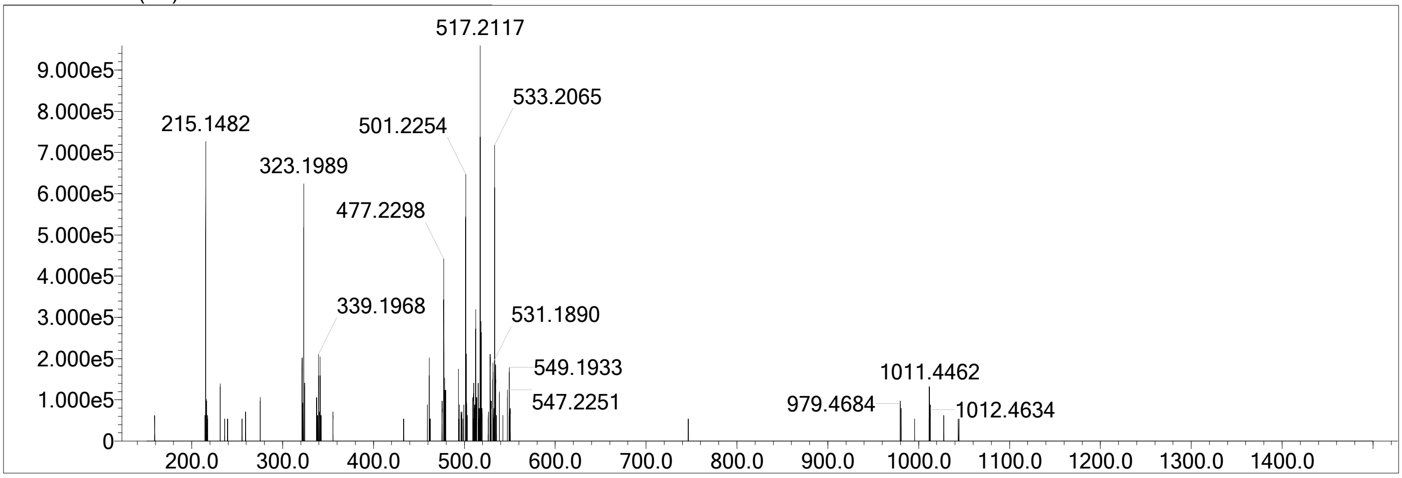

Measured region for 501.2254 m/z

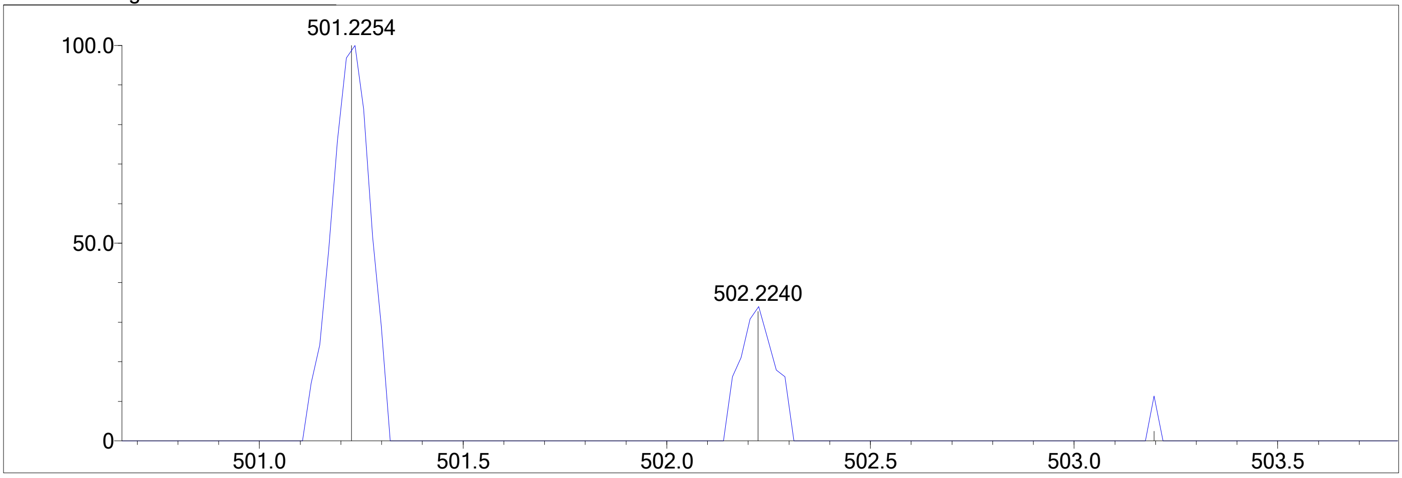

C29 H34 O6 [M+Na]+ : Predicted region for 501.2248 m/z

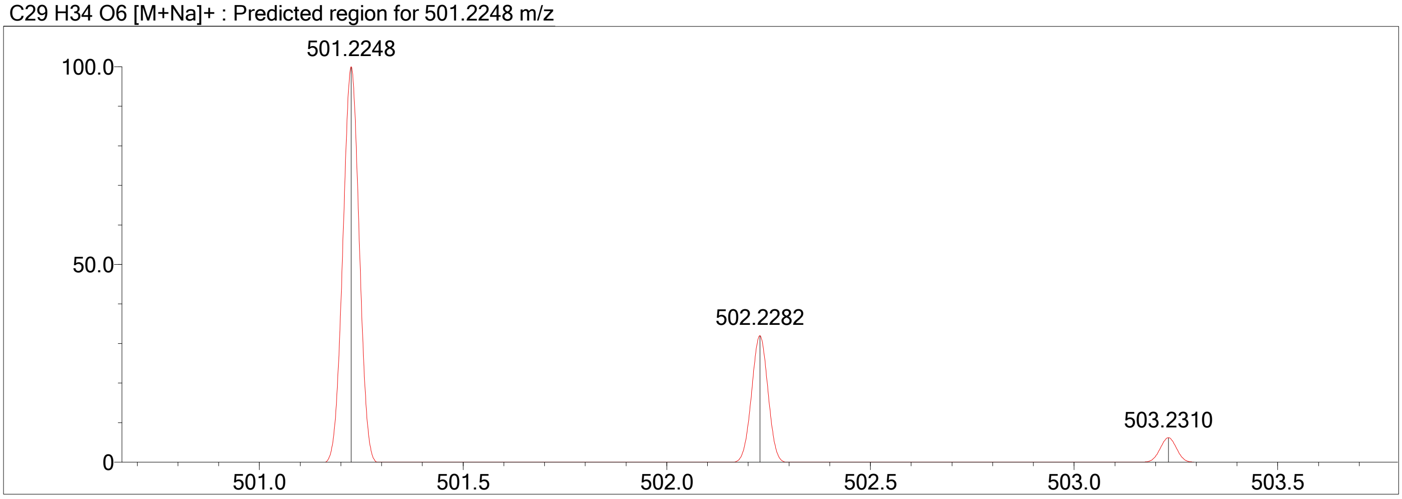

Rank	Score	Formula (M)	Ion	Meas. m/z	Pred. m/z	Df. (mDa)	Df. (ppm)	Iso	DBE
1	87.80	C29 H34 O6	[M+Na]+	501.2254	501.2248	0.6	1.20	88.24	13.0

Figure S11. HR-ESI-MS (E-) Spectrum of Compound **1**

Elmt	Val.	Min	Max	Elmt	Val.	Min	Max	Elmt	Val.	Min	Max	Use Adduct
H	1	30	38	O	2	6	6	Br	1	0	0	H
B	3	0	0	F	1	0	0	I	3	0	0	HCOO
C	4	29	29	S	2	0	0					CH3COO
N	3	0	0	Cl	1	0	0					Cl

Error Margin (mDa):	50.0	DBE Range:	-100.0 - 200.0	Electron Ions:	both
HC Ratio:	0.0 - 100.0	Apply N Rule:	no	Use MSn Info:	no
Max Isotopes:	all	Isotope RI (%):	1.00	Isotope Res:	10000
MSn Iso RI (%):	75.00	MSn Logic Mode:	OR	Max Results:	1000

Event#: 2 MS(E-) Ret. Time : 0.810 Scan# : 122

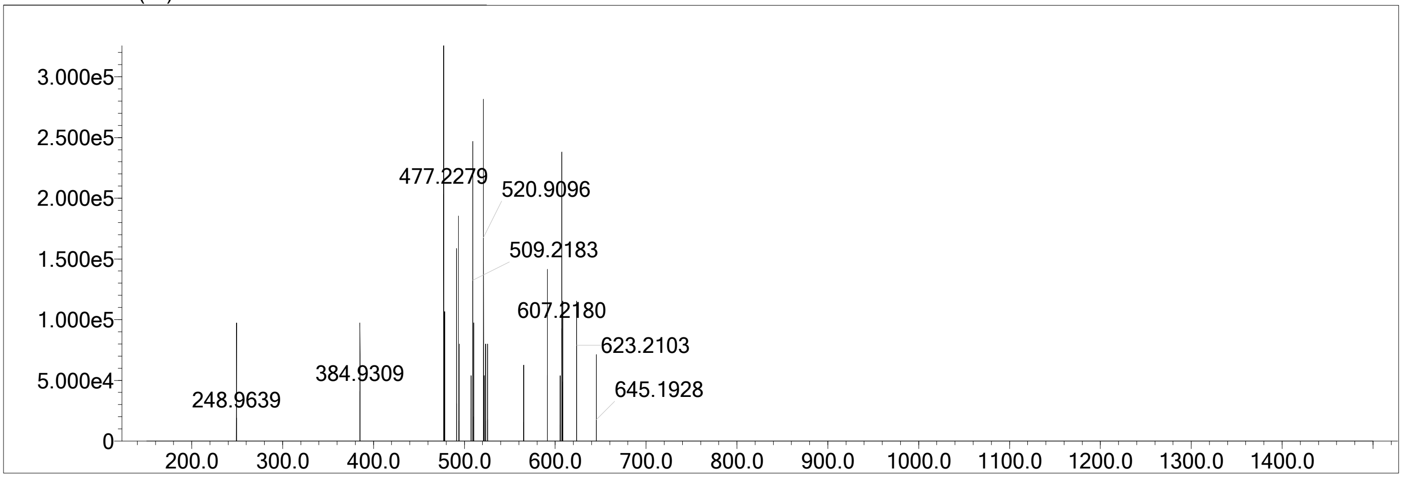

Measured region for 477.2279 m/z

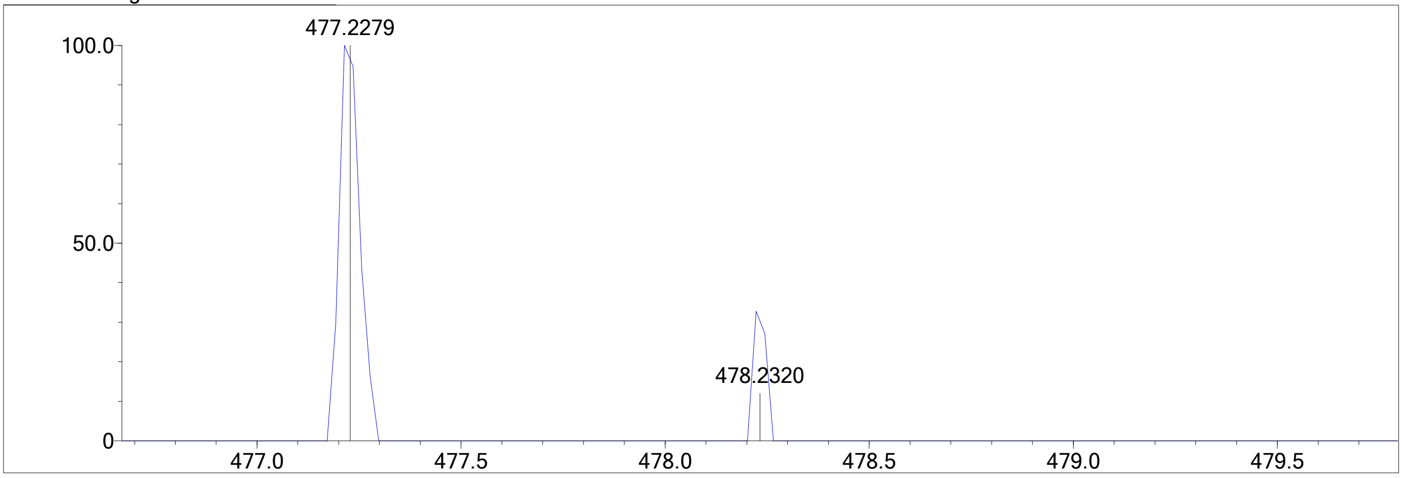

C29 H34 O6 [M-H]- : Predicted region for 477.2283 m/z

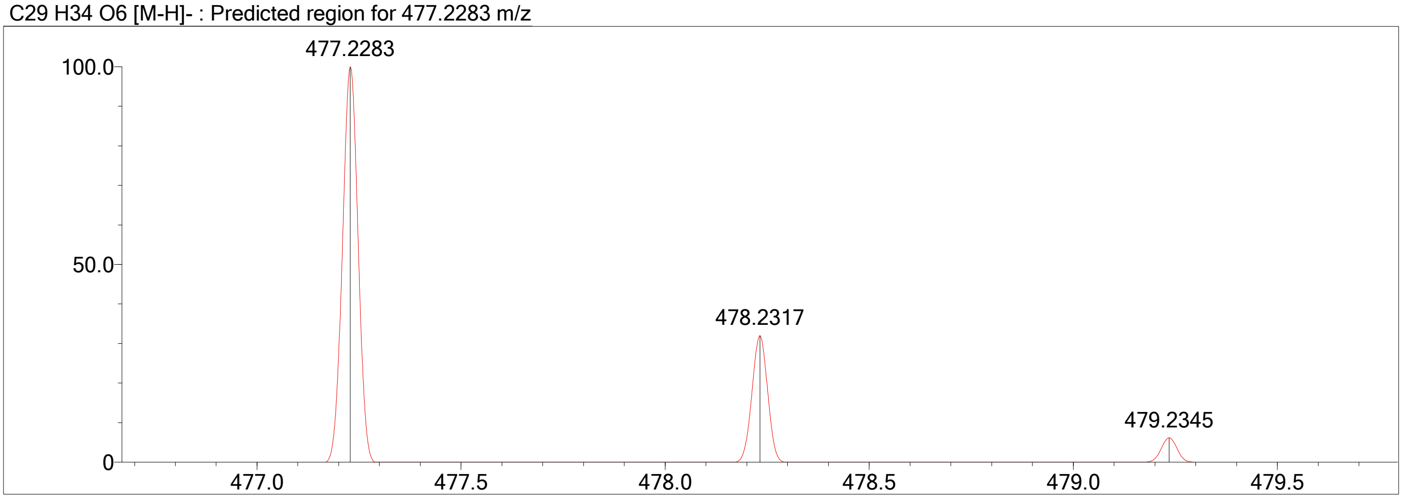

Rank	Score	Formula (M)	Ion	Meas. m/z	Pred. m/z	Df. (mDa)	Df. (ppm)	Iso	DBE
1	84.64	C29 H34 O6	[M-H]-	477.2279	477.2283	-0.4	-0.84	84.64	13.0

Figure S12. ^{1}H NMR (400 MHz, $CDCl_3$) Spectrum of Compound **2**

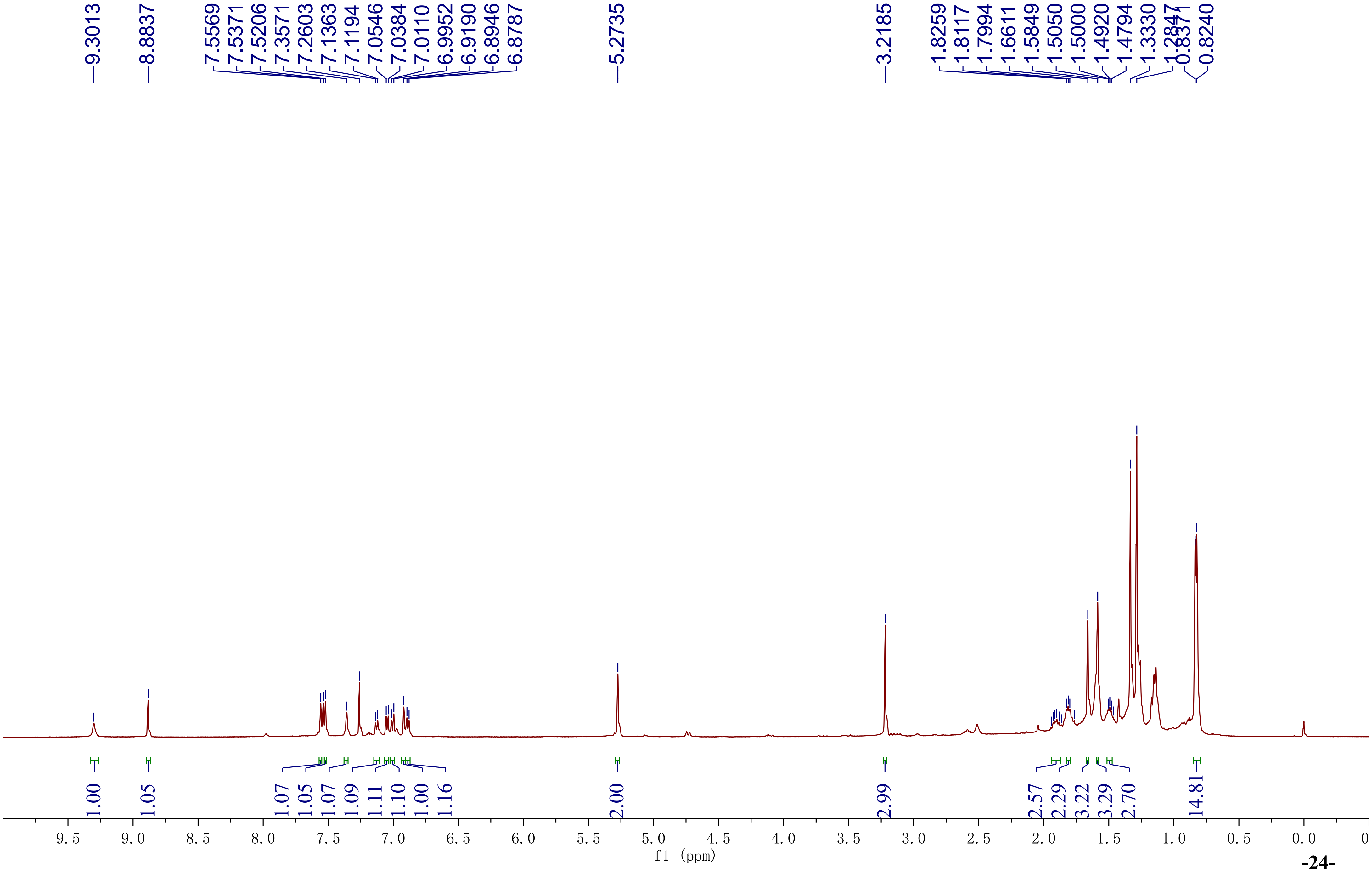

Figure S13. ^{13}C NMR (100 MHz, $CDCl_3$) Spectrum of Compound **2**

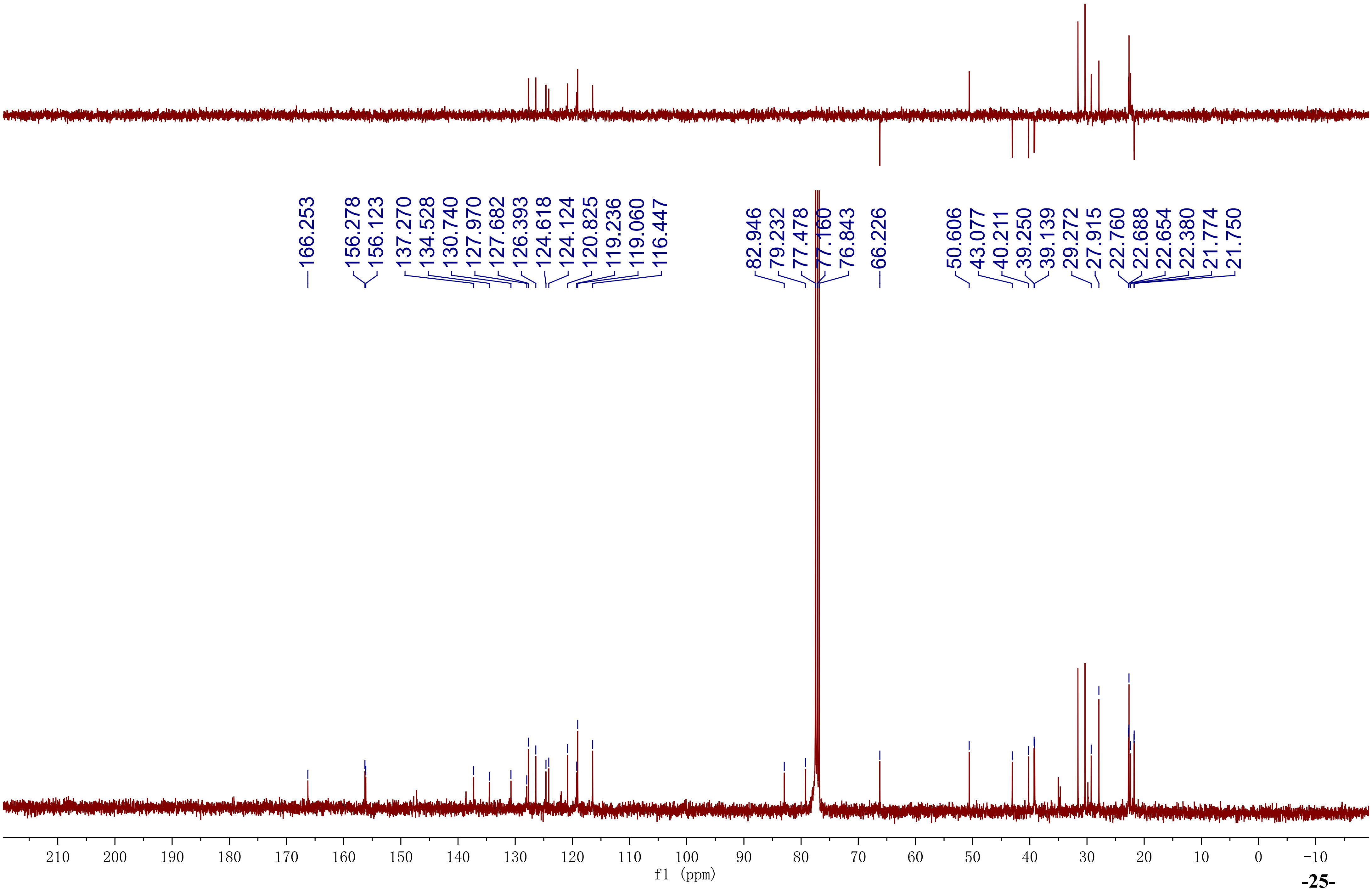

Figure S14. HSQC (400 MHz, $CDCl_3$) Spectrum of Compound **2**

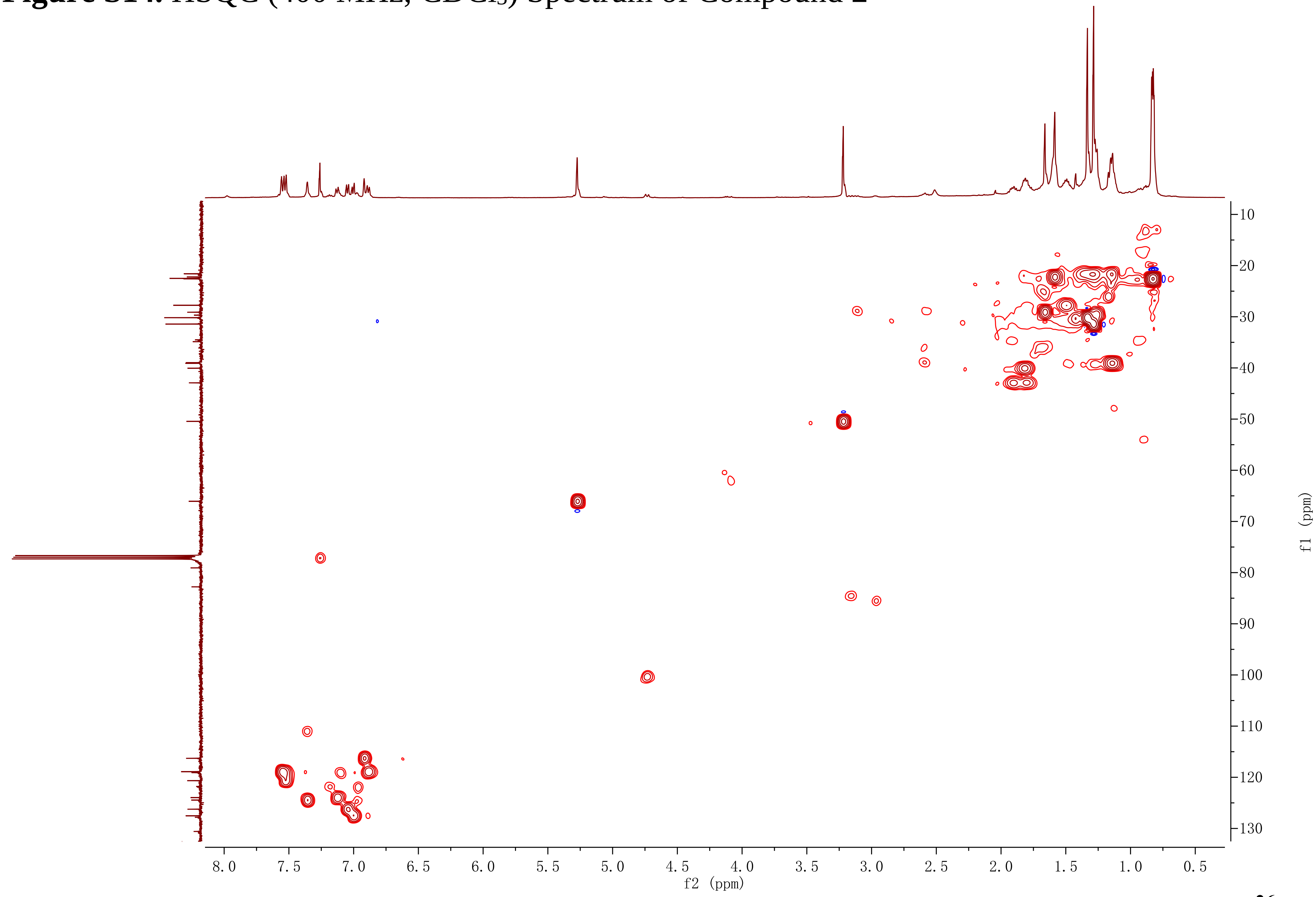

Figure S15. ^{1}H-^{1}H COSY (400 MHz, $CDCl_3$) Spectrum of Compound **2**

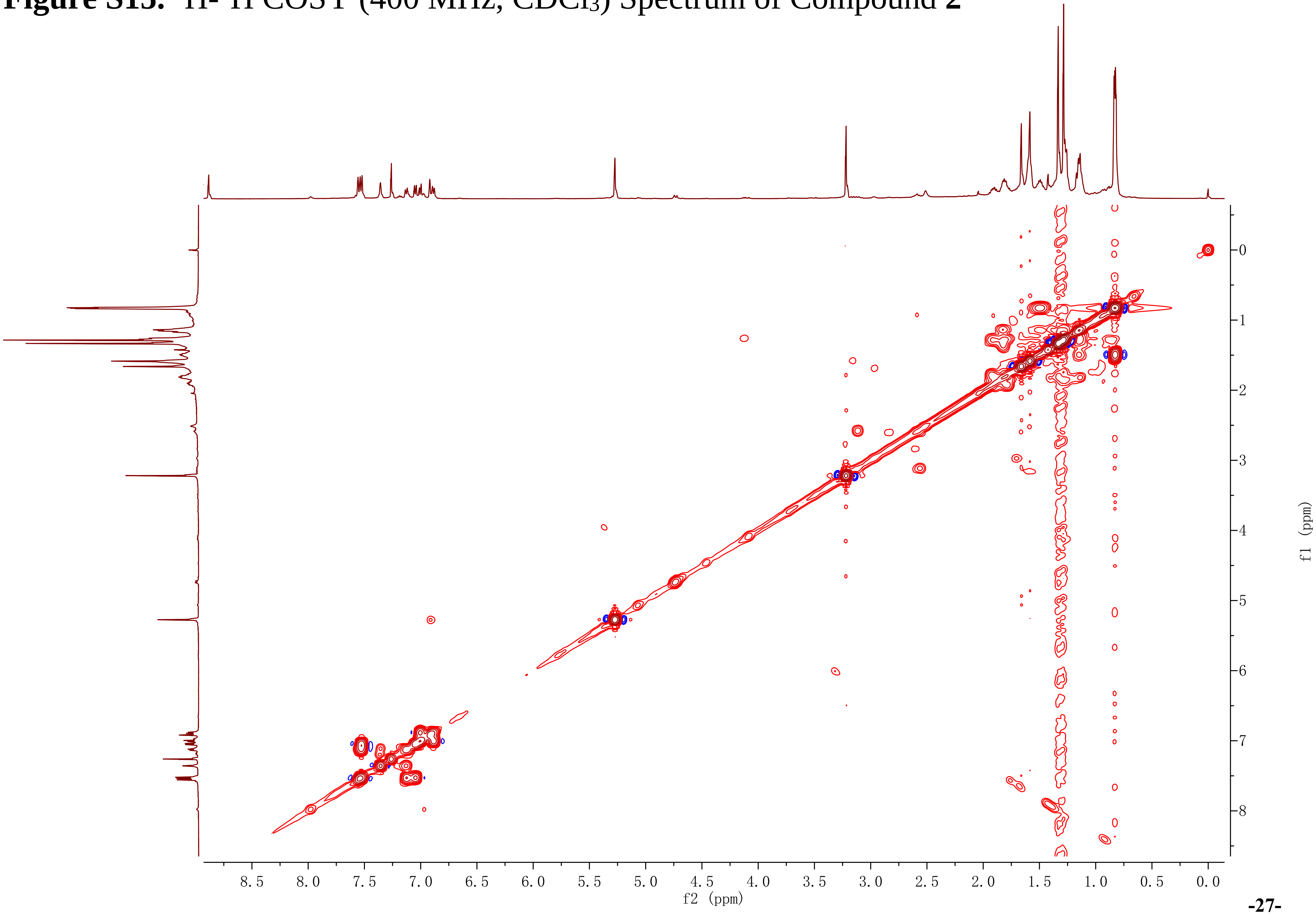

Figure S16. HMBC (400 MHz, CDCl3) Spectrum of Compound **2**

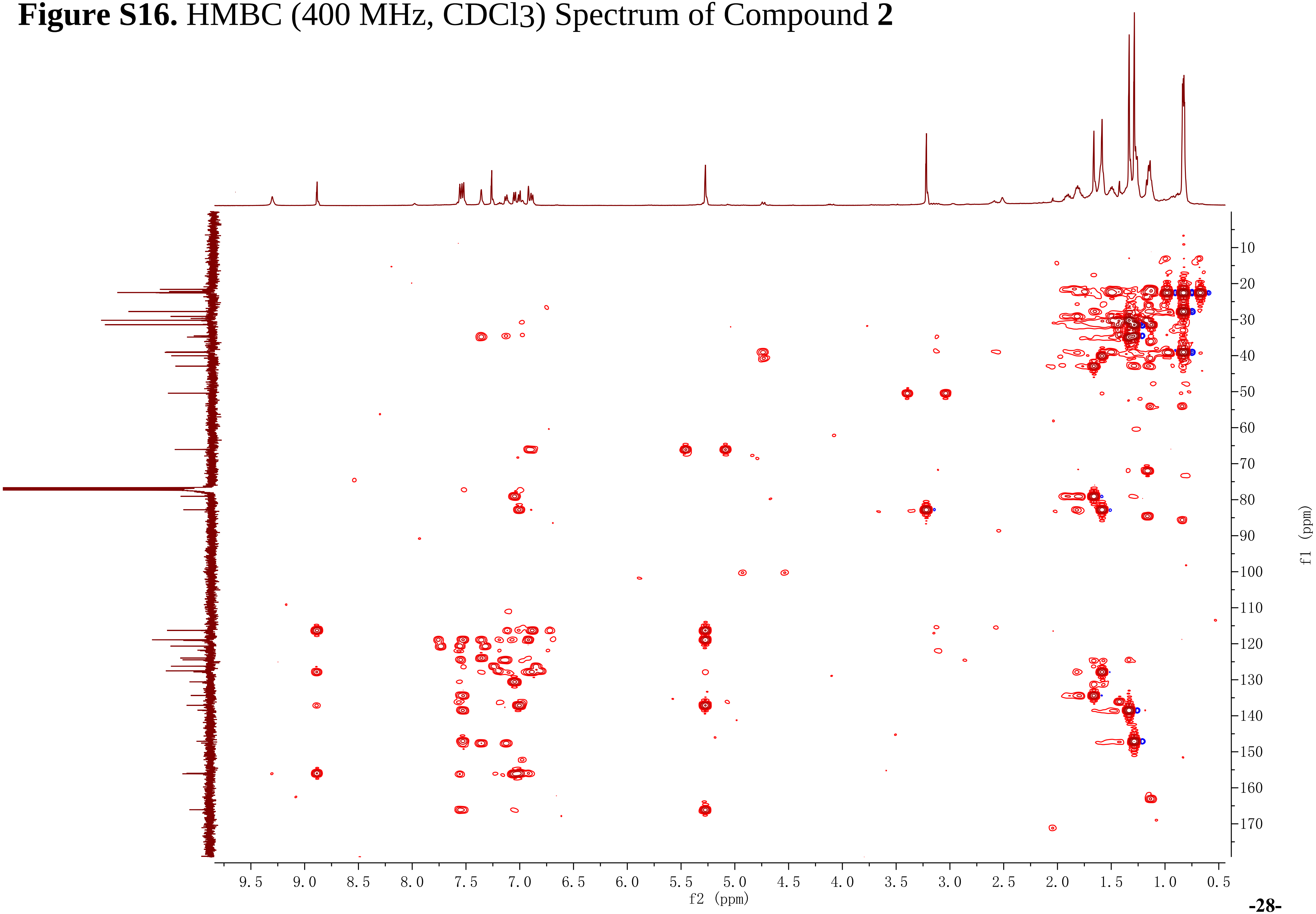

Figure S17. HR-ESI-MS Spectrum of Compound **2**

Elmt	Val.	Min	Max
H	1	46	46
C	4	30	31
N	3	0	2
O	2	3	7

Elmt	Val.	Min	Max
F	1	0	1
S	2	0	0

Use Adduct
H
Na
K
NH4

Error Margin (ppm):	20	DBE Range:	-100.0 - 200.0	Electron Ions:	both
HC Ratio:	0.0 - 100.0	Apply N Rule:	no	Use MSn Info:	no
Max Isotopes:	all	Isotope RI (%):	1.00	Isotope Res:	10000
MSn Iso RI (%):	75.00	MSn Logic Mode:	OR	Max Results:	1000

Event#: 1 MS(E+) Ret. Time : 1.463 Scan# : 219

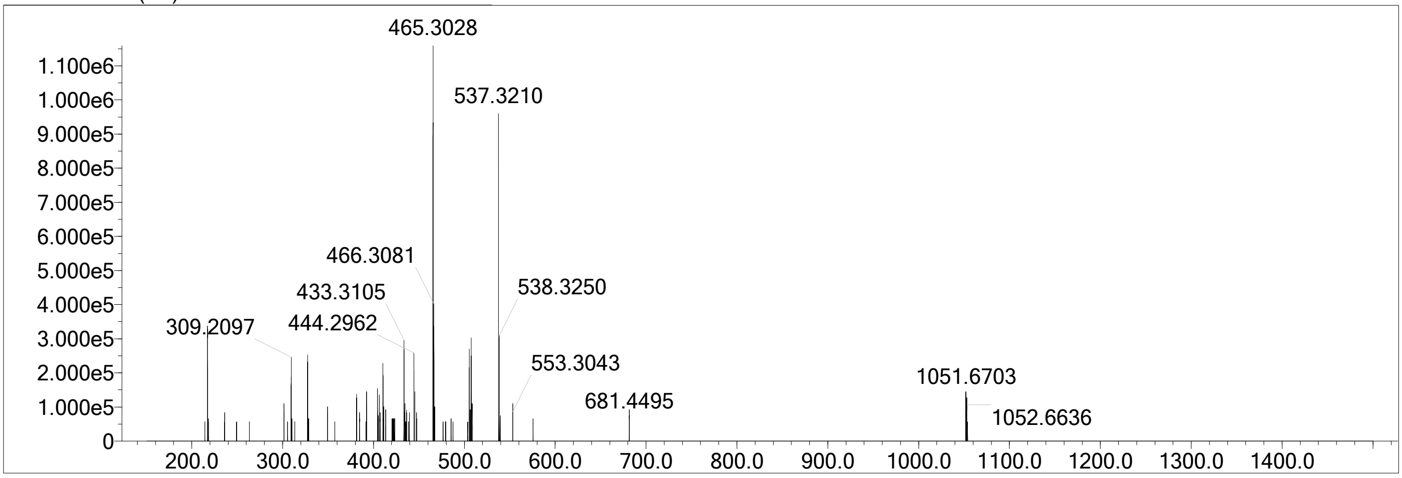

Measured region for 537.3210 m/z

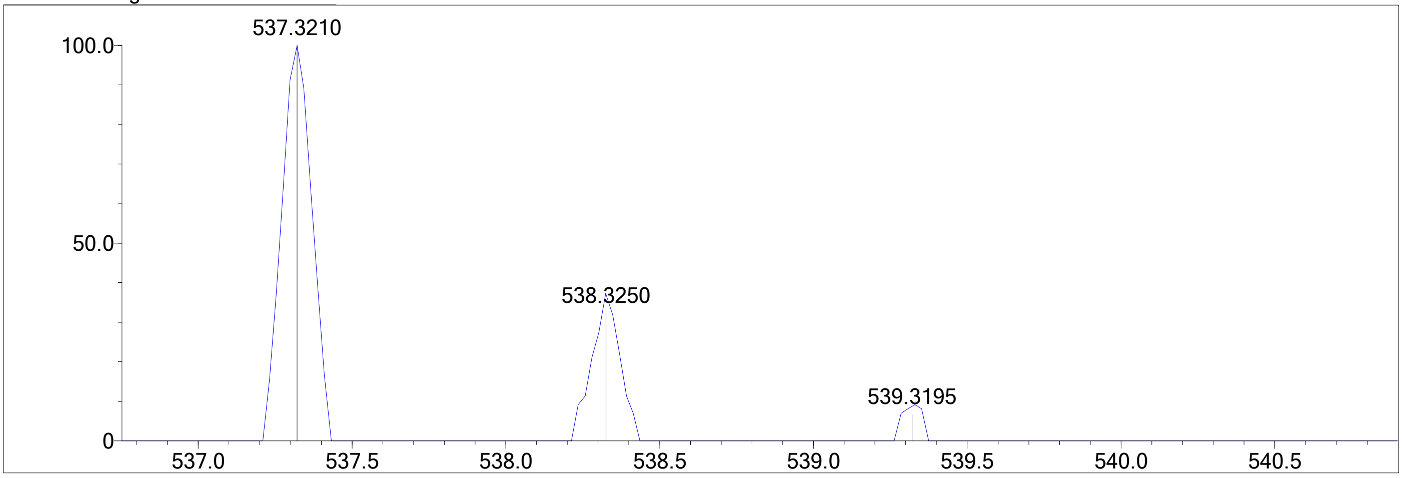

C31 H46 O6 [M+Na]+ : Predicted region for 537.3187 m/z

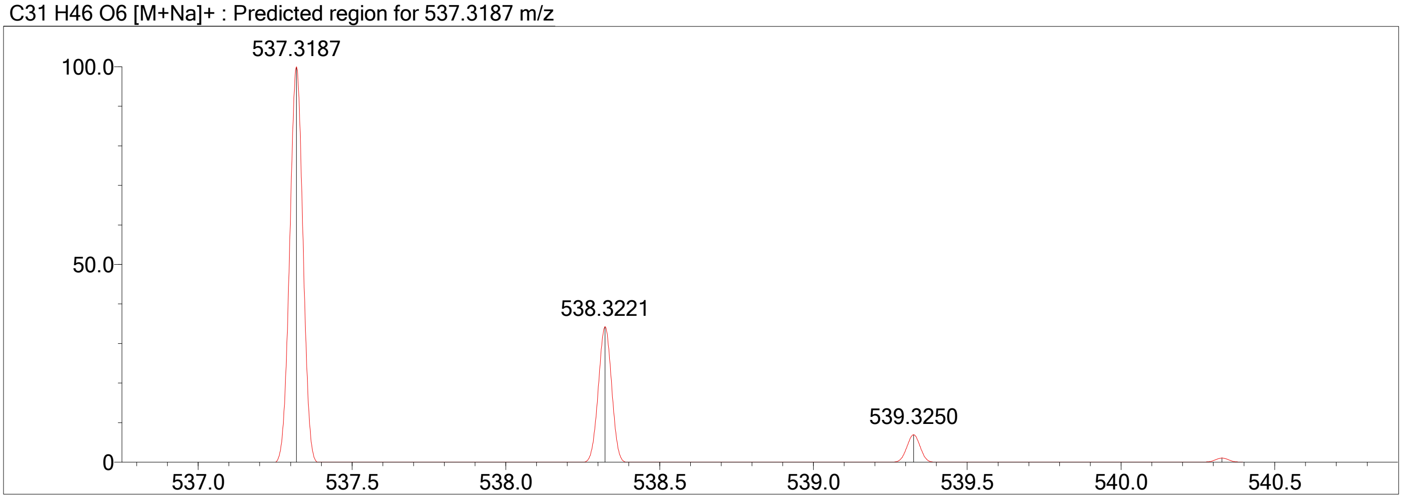

Rank	Score	Formula (M)	Ion	Meas. m/z	Pred. m/z	Df. (mDa)	Df. (ppm)	Iso	DBE
1	79.09	C31 H46 O6	[M+Na]+	537.3210	537.3187	2.3	4.28	86.16	9.0

Figure S18. ^{1}H NMR (500 MHz, $CDCl_3$) Spectrum of Compound **3**

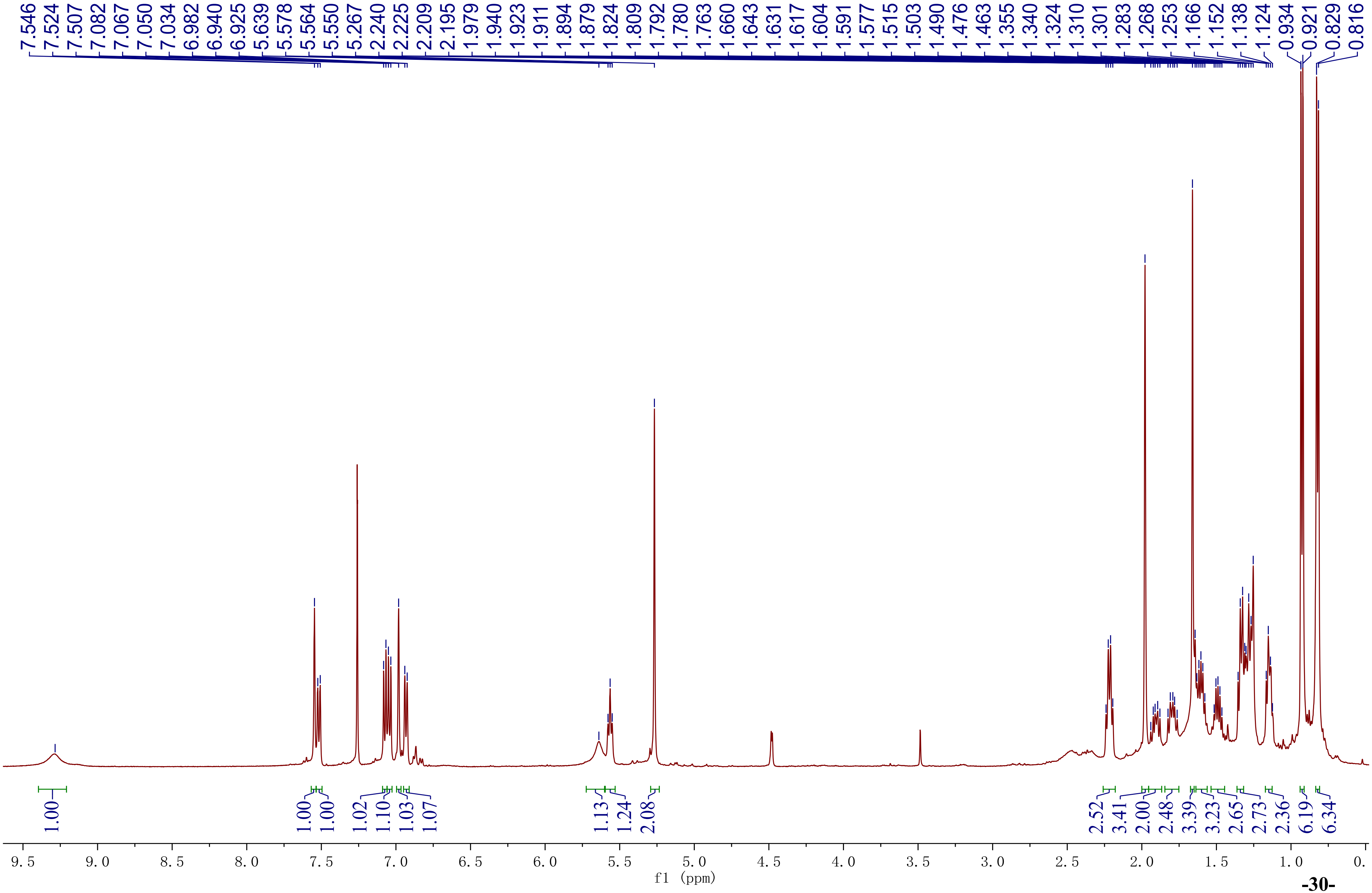

Figure S19. ^{13}C NMR (125 MHz, $CDCl_3$) Spectrum of Compound **3**

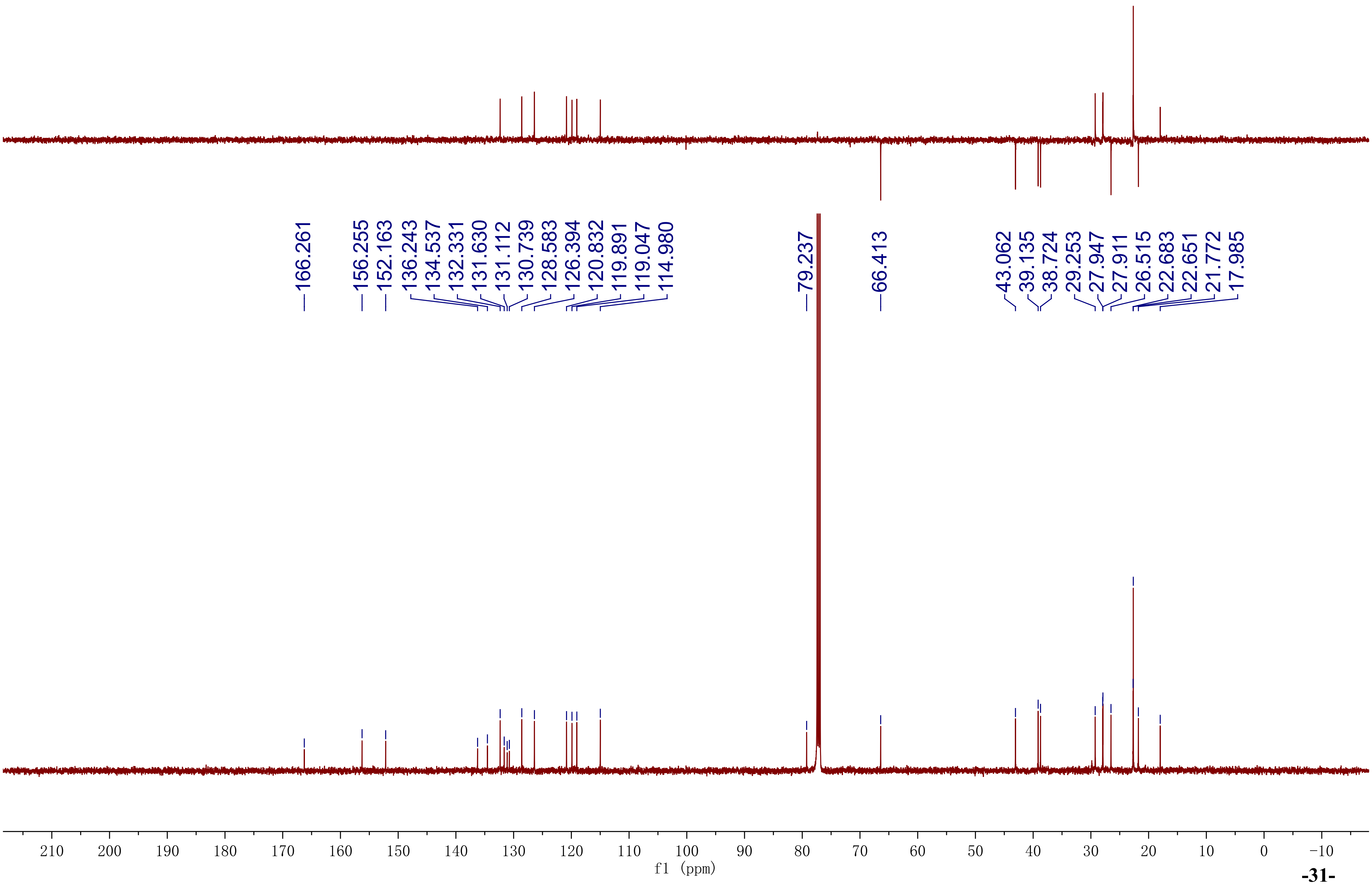

Figure S20. HSQC (500 MHz, $CDCl_3$) Spectrum of Compound **3**

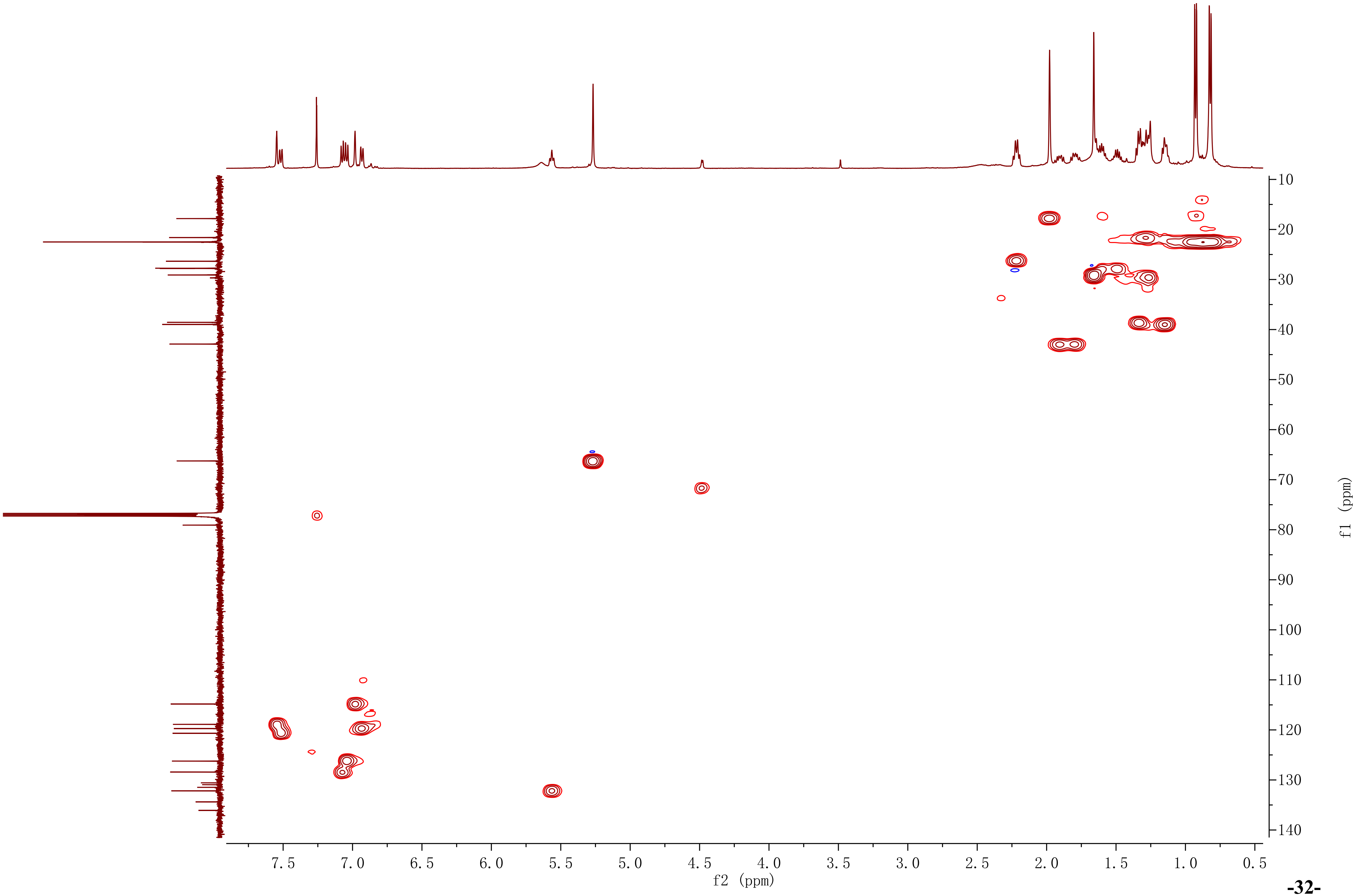

Figure S21. ^{1}H-^{1}H COSY (500 MHz, $CDCl_3$) Spectrum of Compound **3**

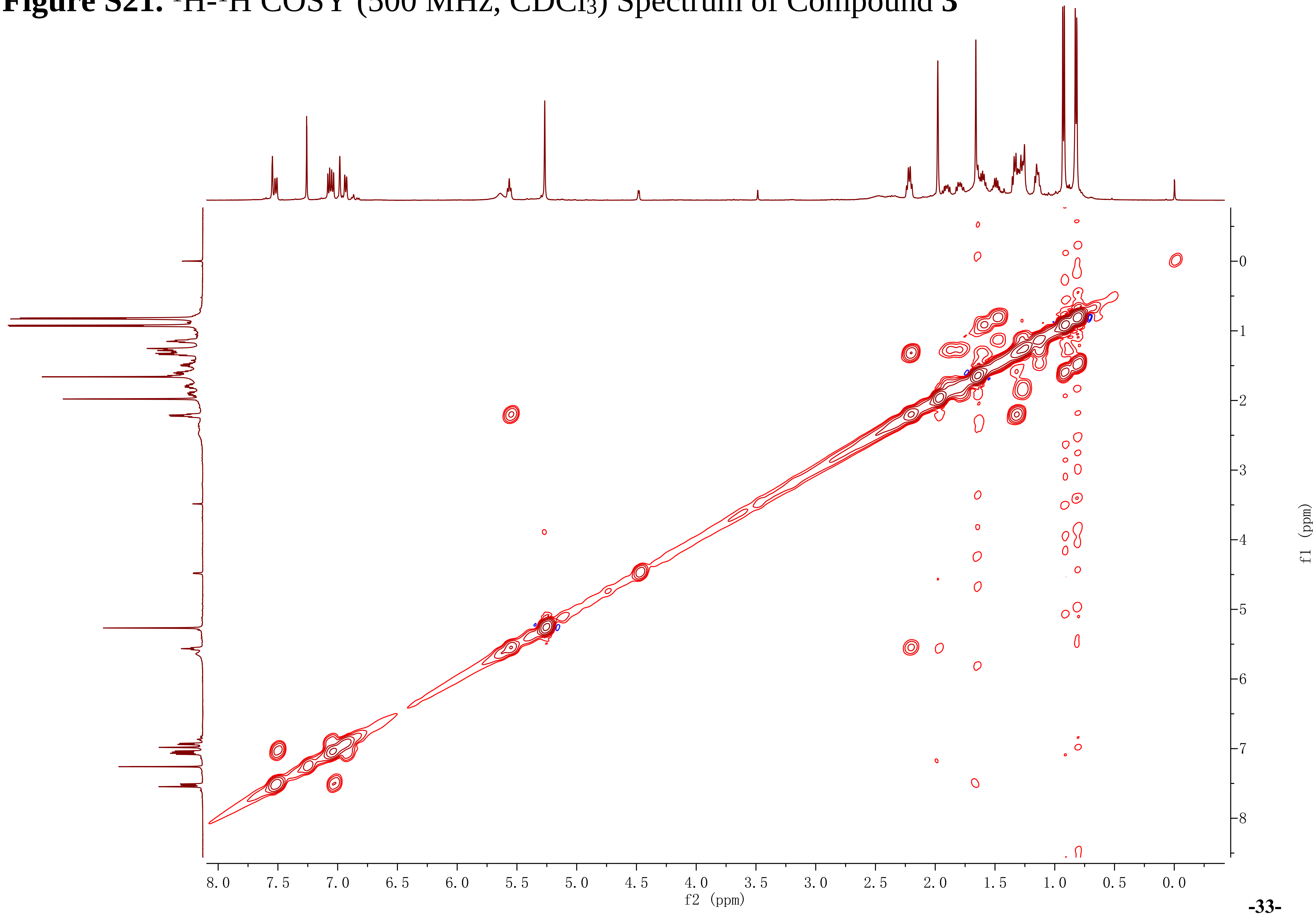

Figure S22. HMBC (500 MHz, $CDCl_3$) Spectrum of Compound **3**

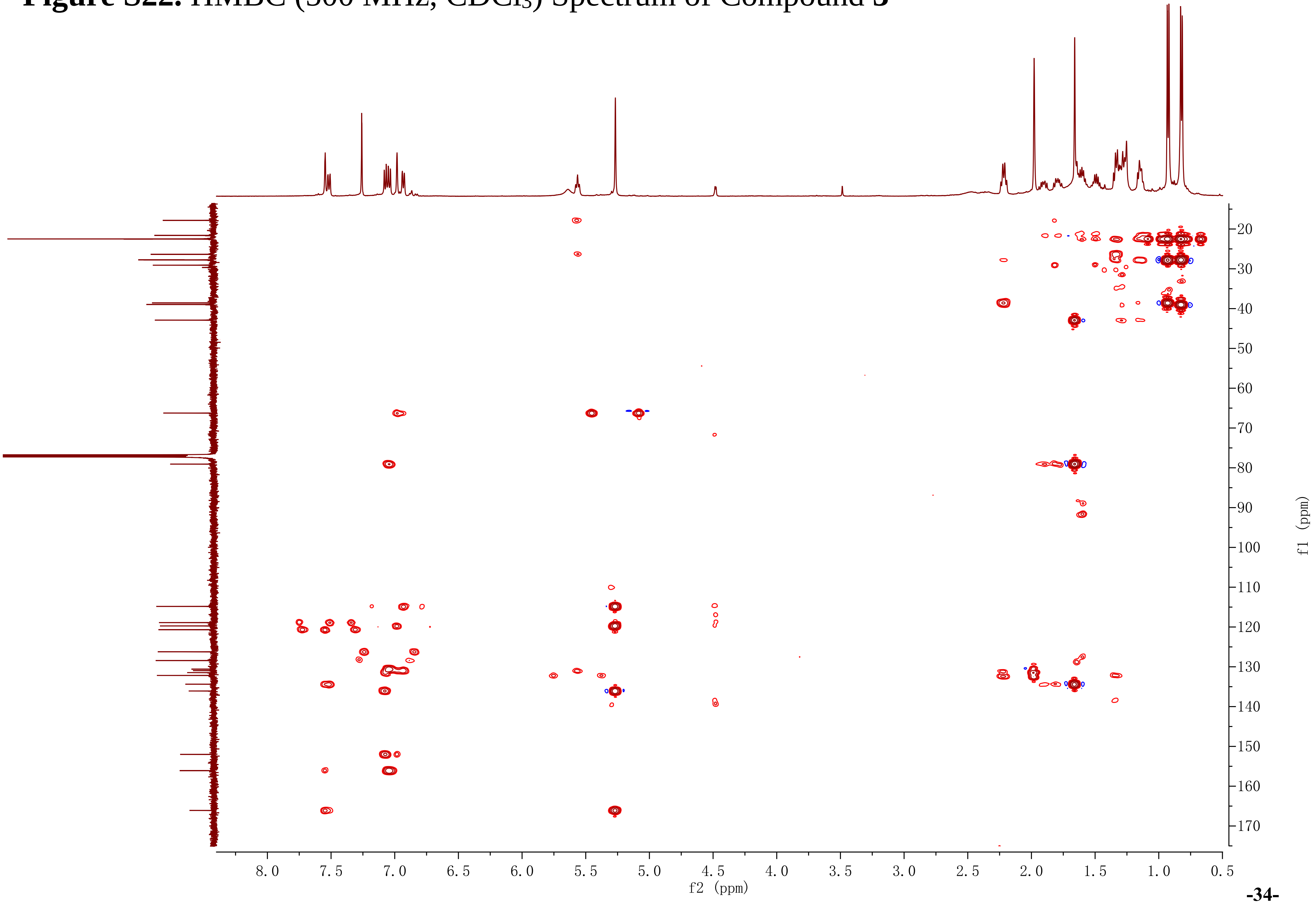

Figure S23. HR-ESI-MS Spectrum of Compound **3**

Elmt	Val.	Min	Max	Elmt	Val.	Min	Max	Elmt	Val.	Min	Max	Elmt	Val.	Min	Max	Use Adduct
H	1	42	42	O	2	5	5	S	2	0	0	Br	1	0	0	H
B	3	0	0	F	1	0	0	Cl	1	0	0	I	3	0	0	Na
C	4	30	30	Si	4	0	0	As	3	0	0	Au	1	0	0	K
N	3	0	0	P	3	0	0	Se	2	0	0					NH4

Error Margin (ppm):	100	DBE Range:	-100.0 - 200.0	Electron Ions:	both
HC Ratio:	0.0 - 100.0	Apply N Rule:	no	Use MSn Info:	no
Max Isotopes:	all	Isotope RI (%):	1.00	Isotope Res:	10000
MSn Iso RI (%):	75.00	MSn Logic Mode:	OR	Max Results:	1000

Event#: 1 MS(E+) Ret. Time : 1.503 Scan# : 225

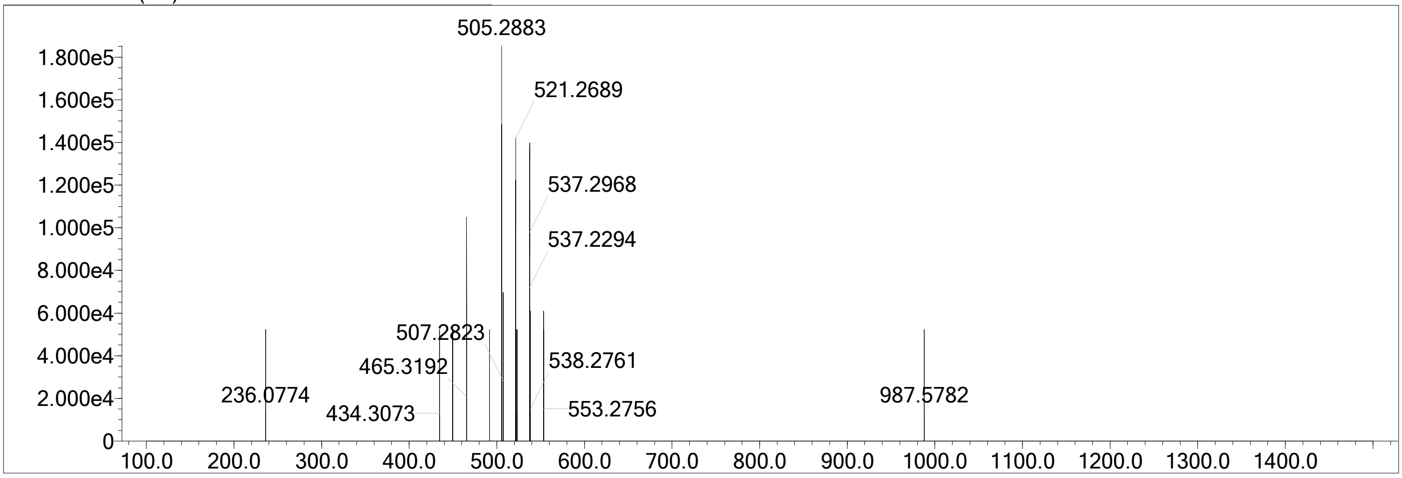

Measured region for 521.2689 m/z

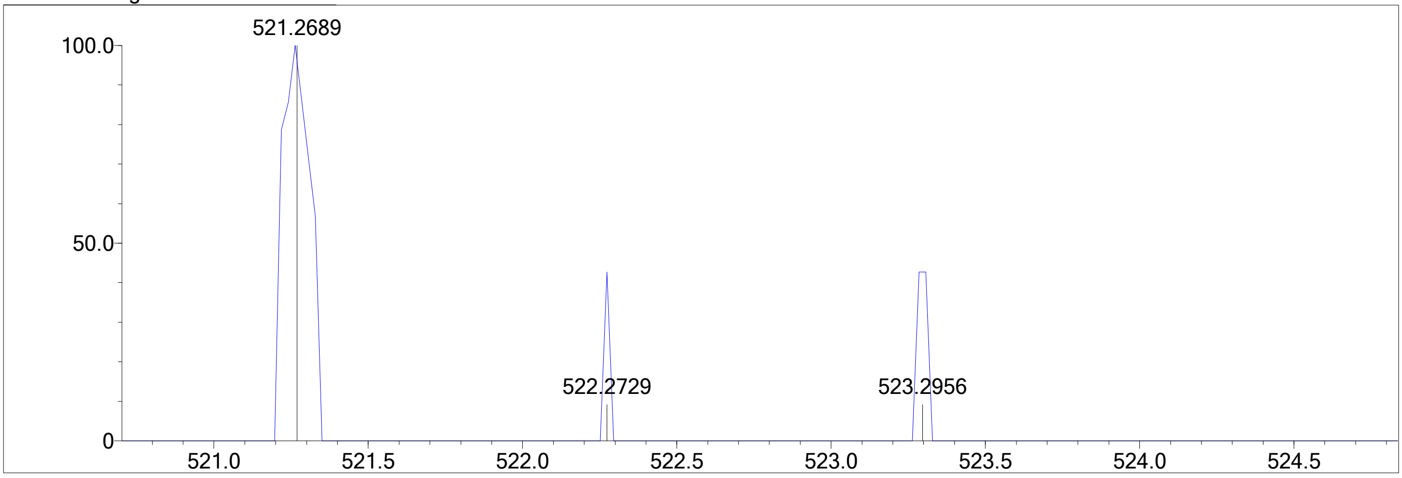

C30 H42 O5 [M+K]+ : Predicted region for 521.2664 m/z

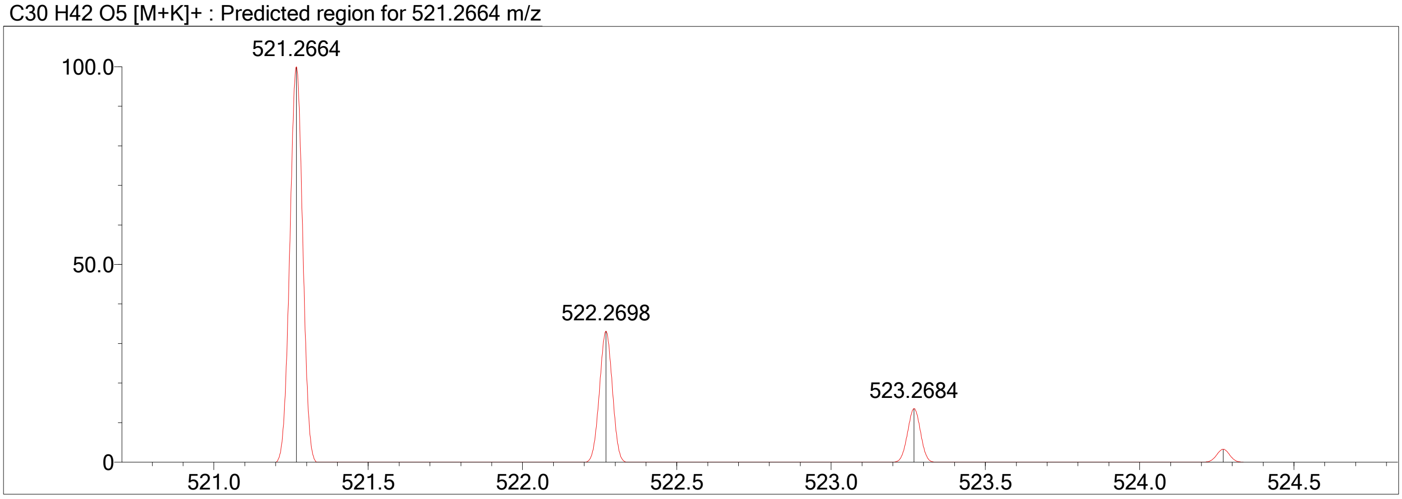

Rank	Score	Formula (M)	Ion	Meas. m/z	Pred. m/z	Df. (mDa)	Df. (ppm)	Iso	DBE
1	41.53	C30 H42 O5	[M+K]+	521.2689	521.2664	2.5	4.80	45.89	10.0

Figure S24. ^{1}H NMR (400 MHz, $CDCl_3$) Spectrum of Compound **4**

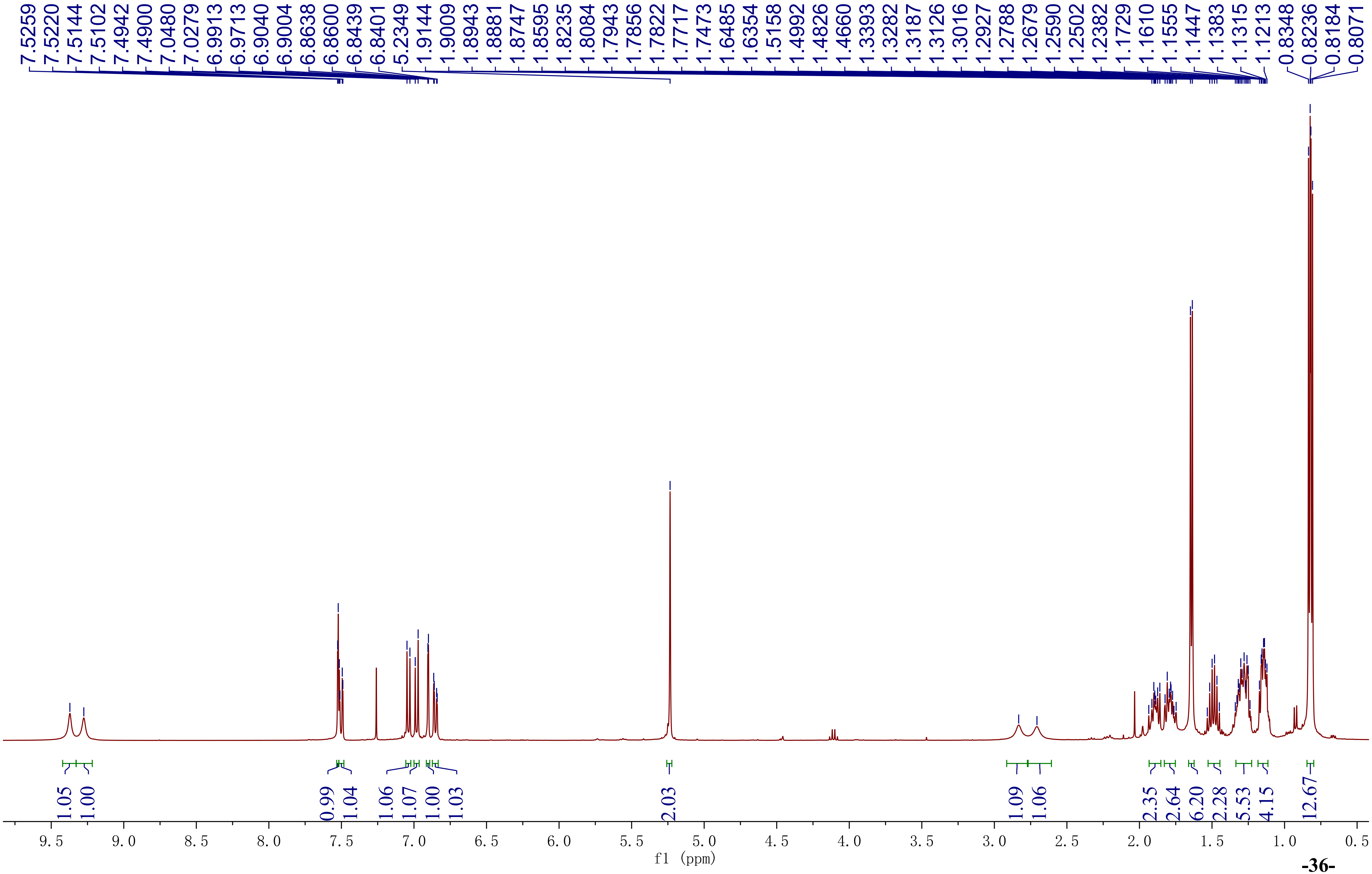

Figure S25. ^{13}C NMR (100 MHz, $CDCl_3$) Spectrum of Compound **4**

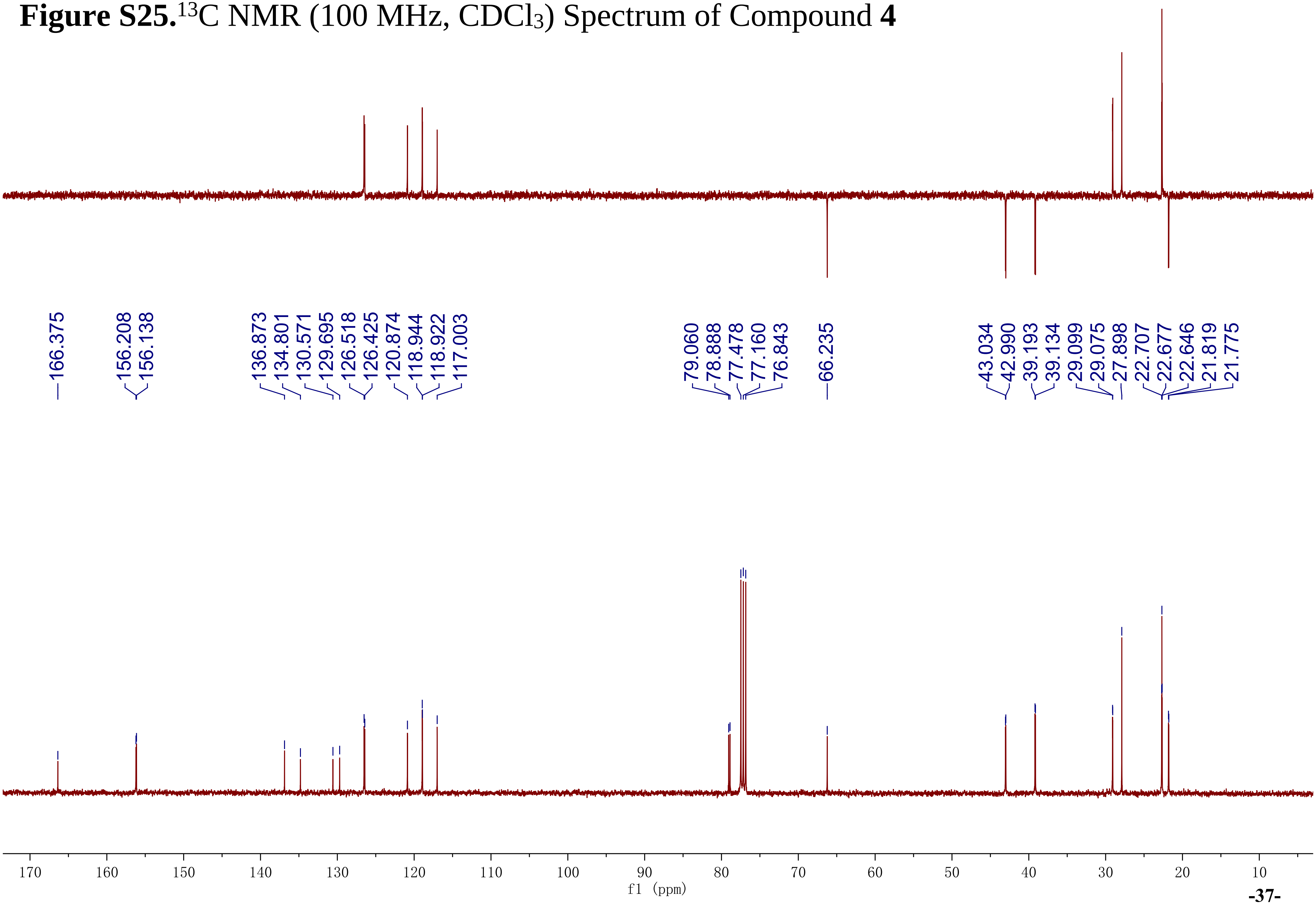

Figure S26. HSQC (400 MHz, $CDCl_3$) Spectrum of Compound **4**

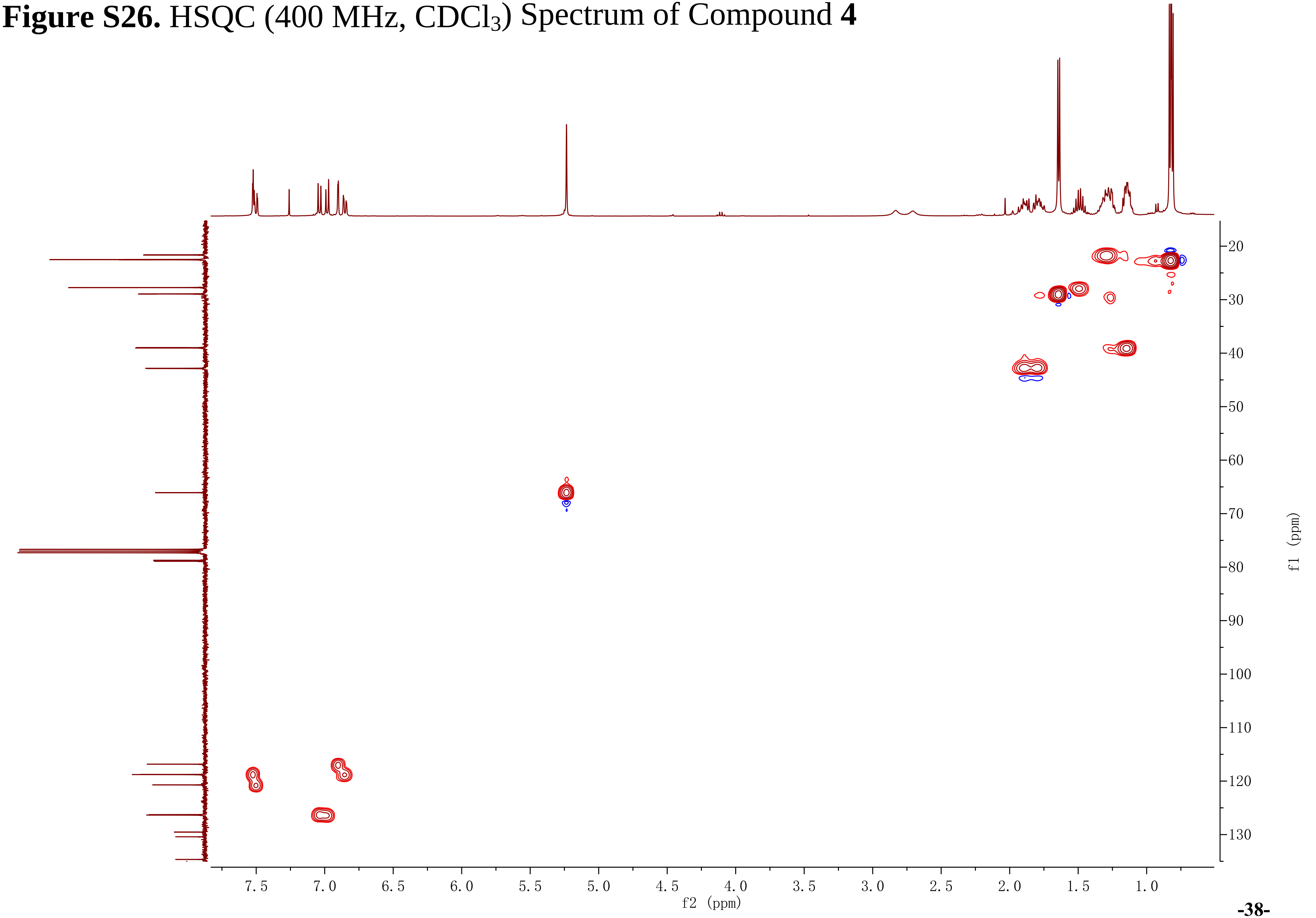

Figure S27. ^{1}H-^{1}H COSY (400 MHz, $CDCl_3$) Spectrum of Compound **4**

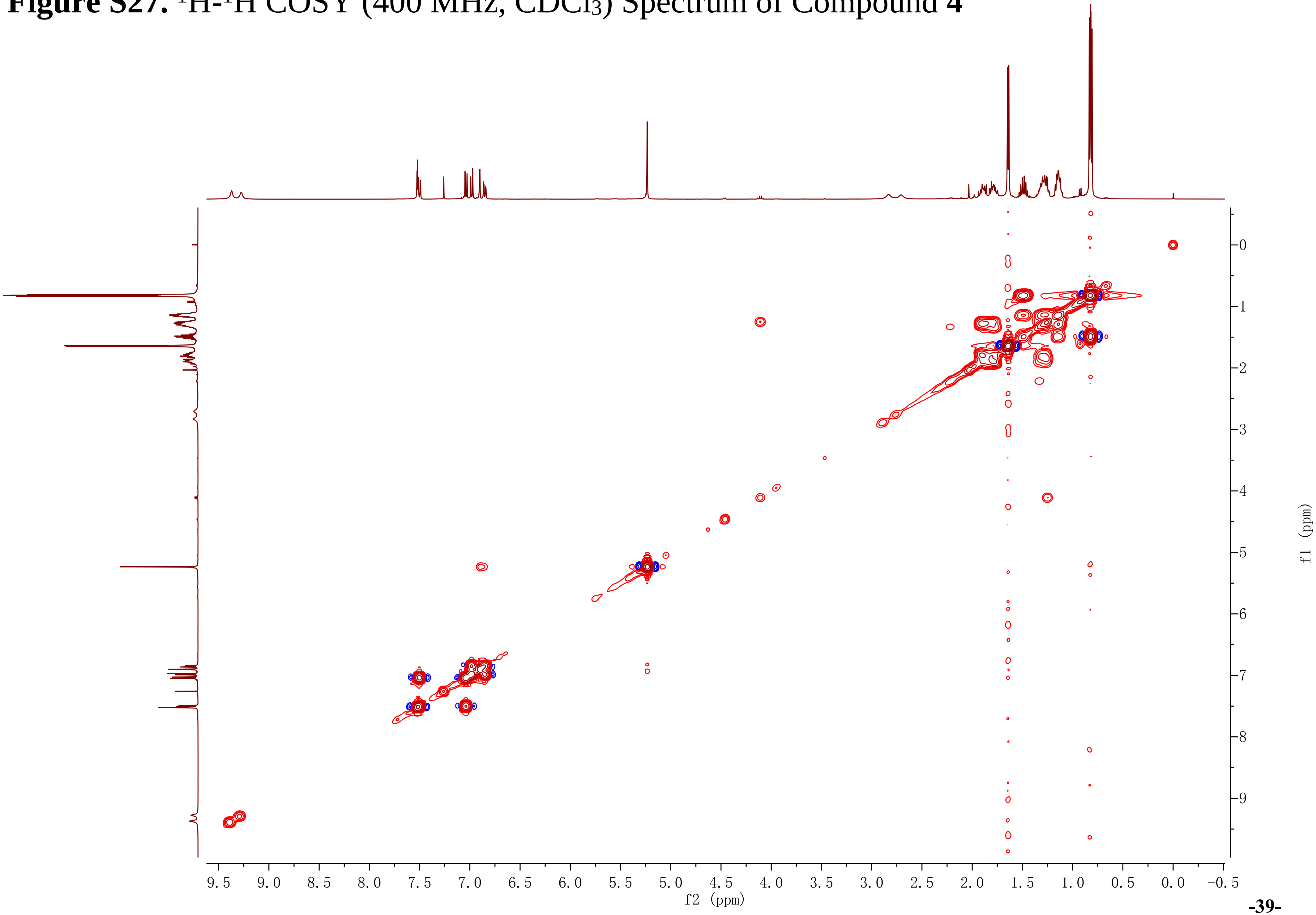

Figure S28. HMBC (400 MHz, $CDCl_3$) Spectrum of Compound **4**

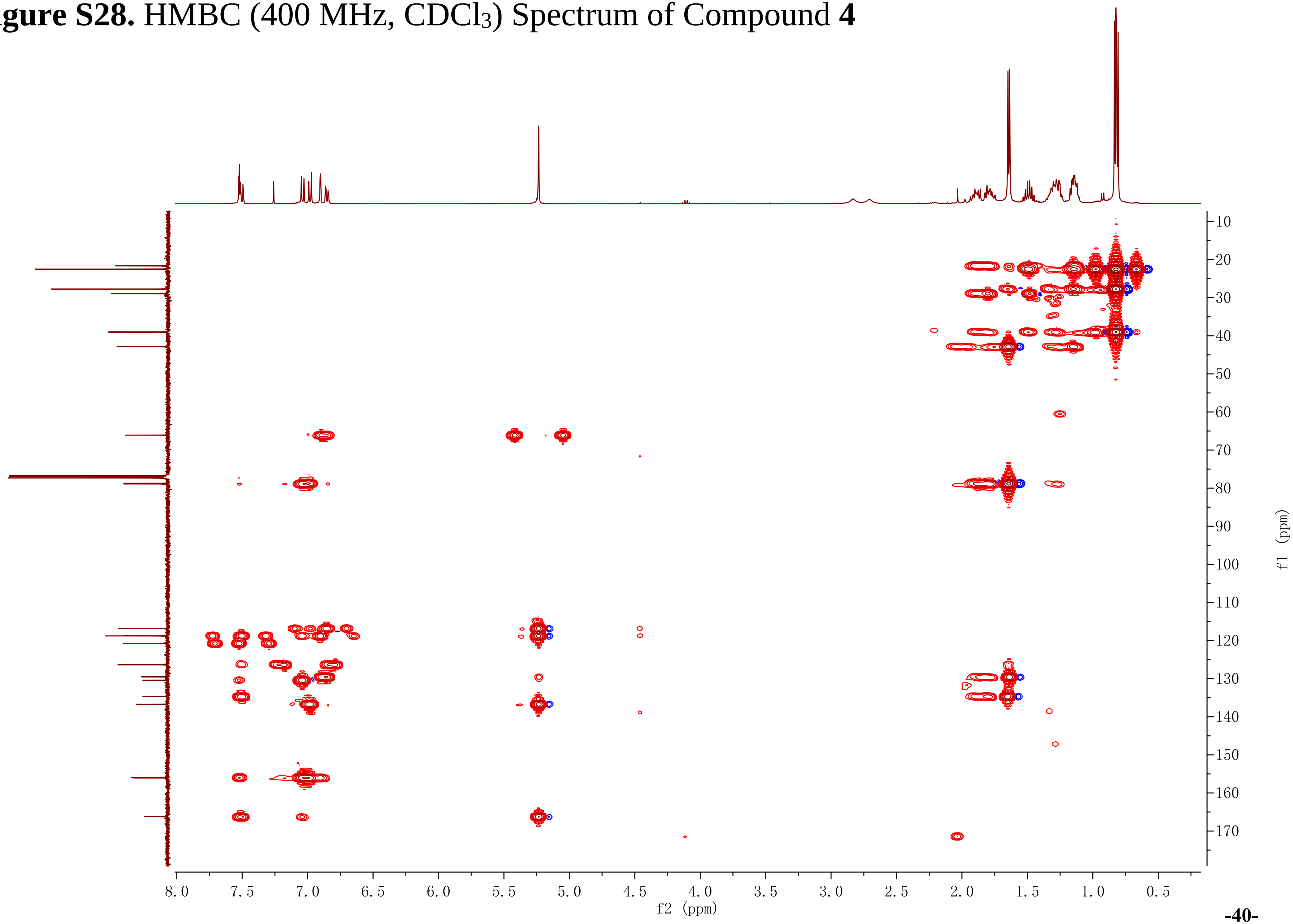

Figure S29. ^{1}H NMR (400 MHz, $CDCl_3$) Spectrum of Compound **5**

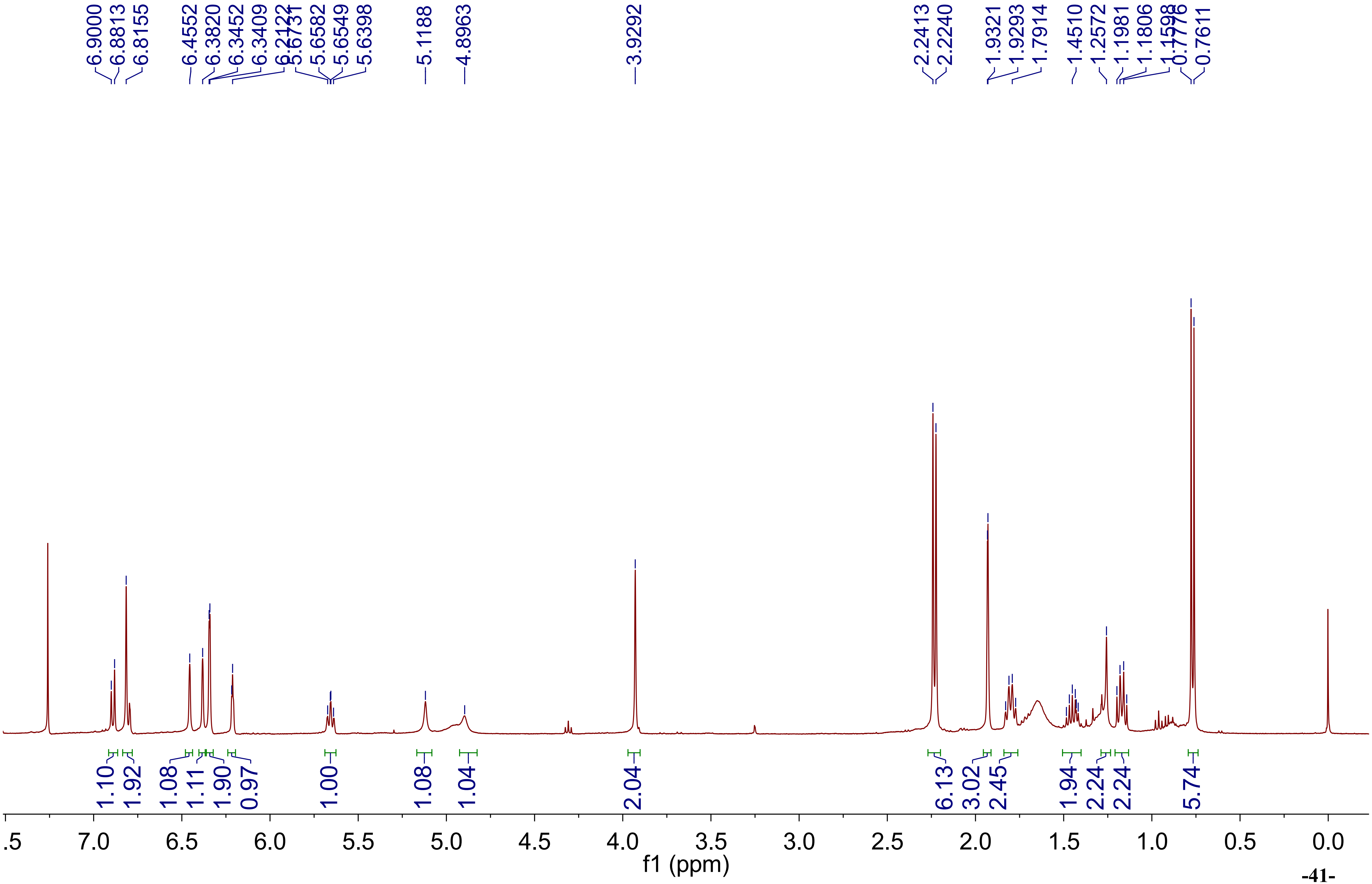

Figure S30.^{13}C NMR (100 MHz, $CDCl_3$) Spectrum of Compound **5**

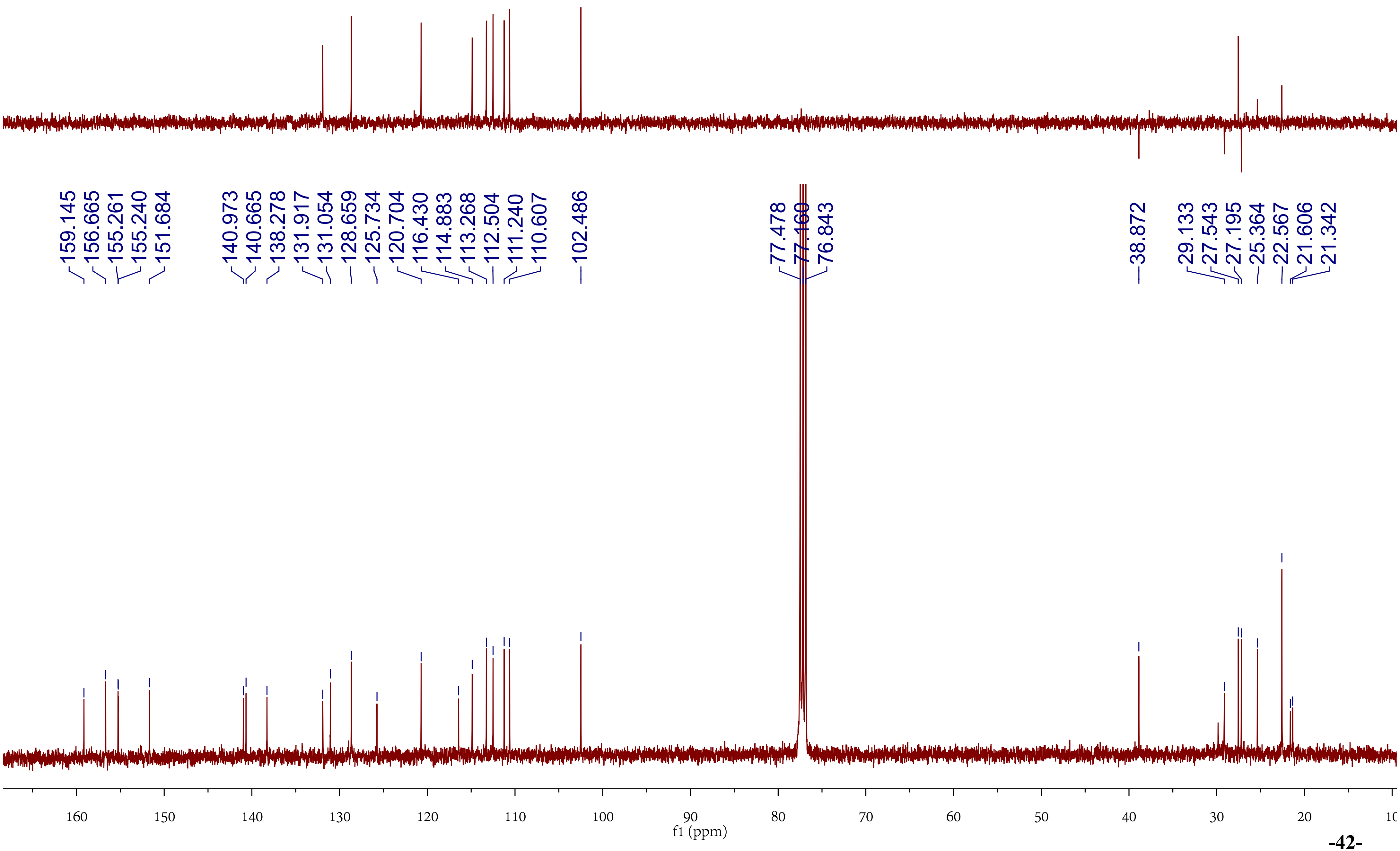

Figure S31. HSQC (400 MHz, $CDCl_3$) Spectrum of Compound **5**

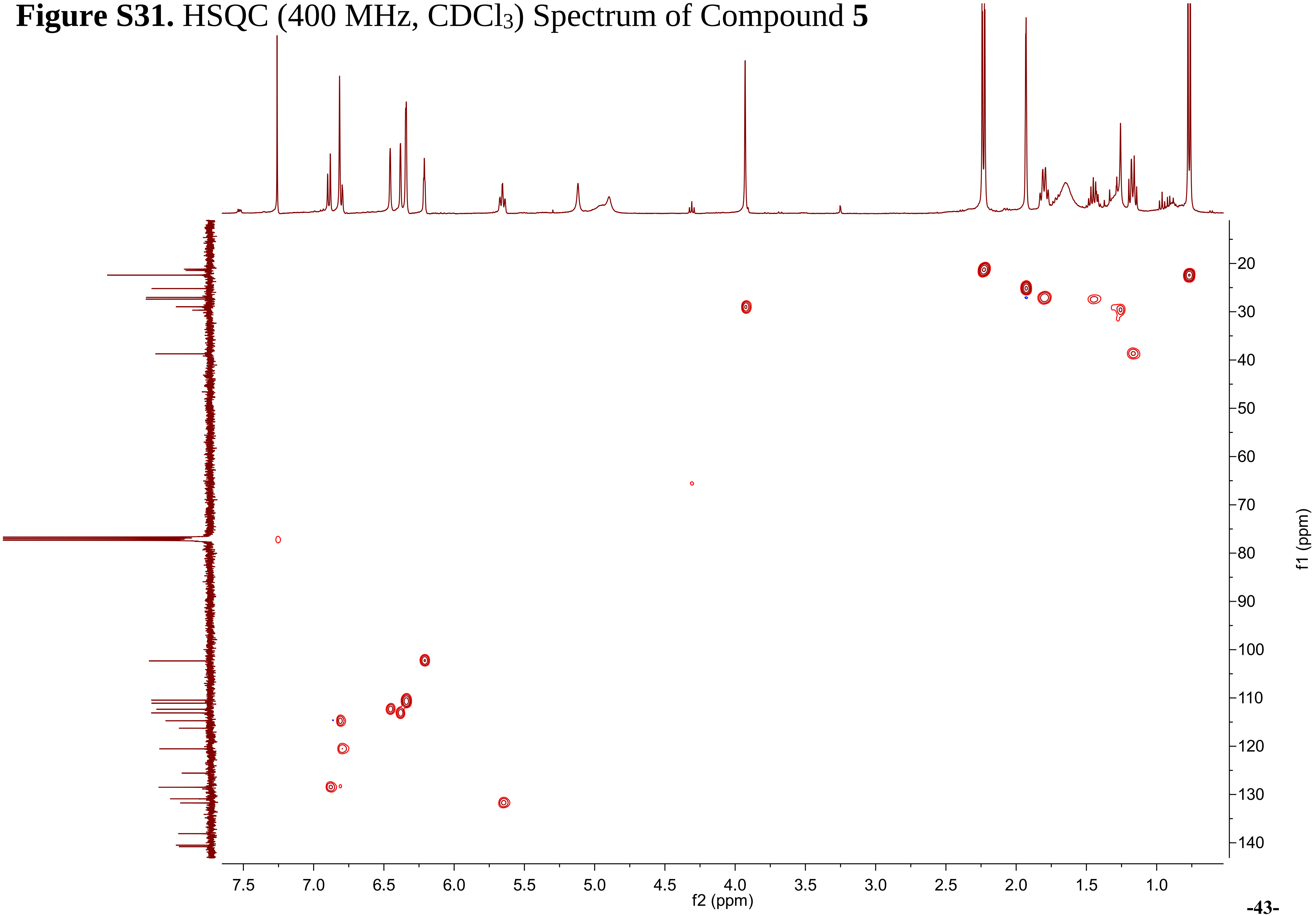

Figure S32. ^{1}H-^{1}H COSY (400 MHz, $CDCl_3$) Spectrum of Compound **5**

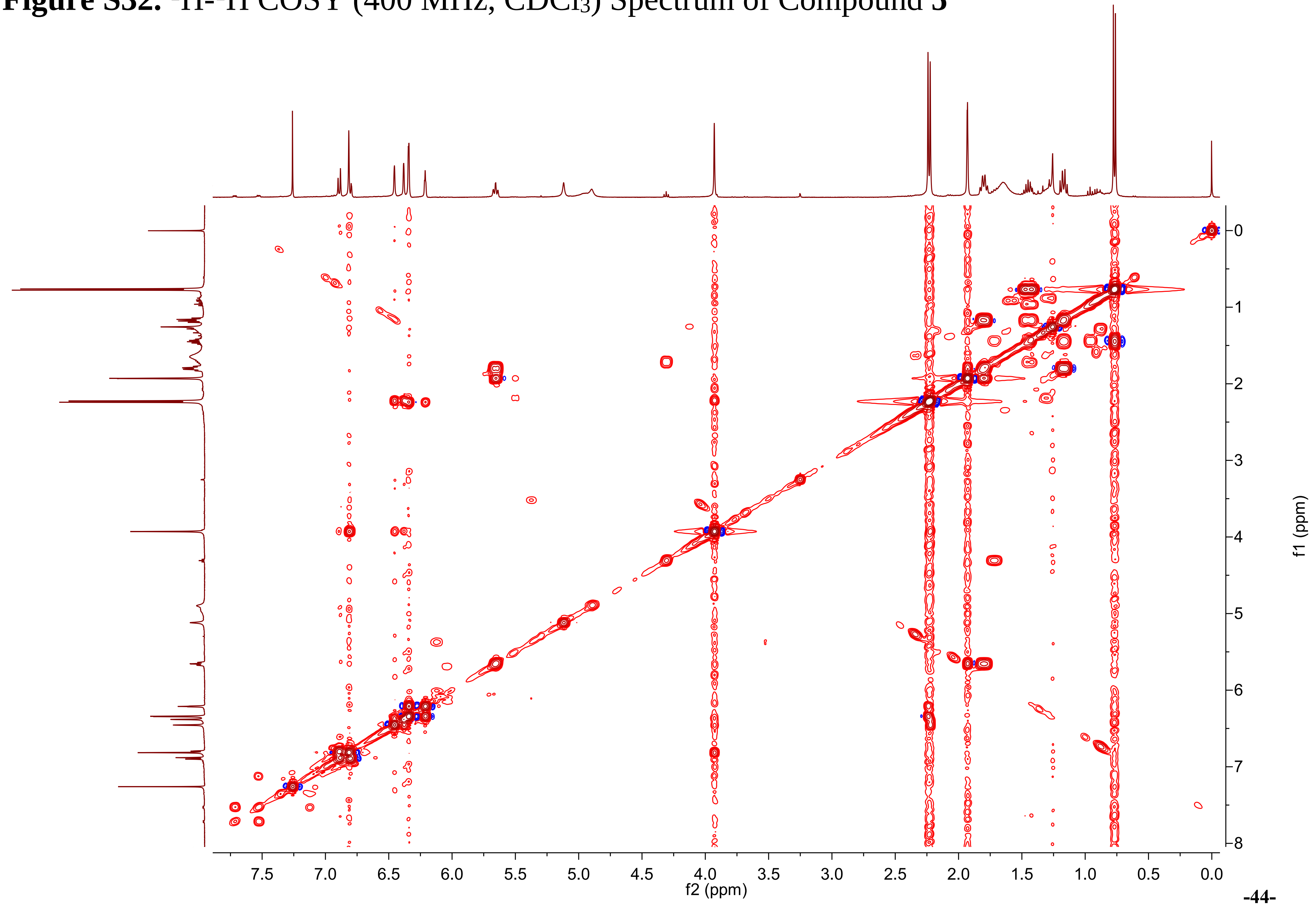

Figure S33. HMBC (400 MHz, $CDCl_3$) Spectrum of Compound **5**

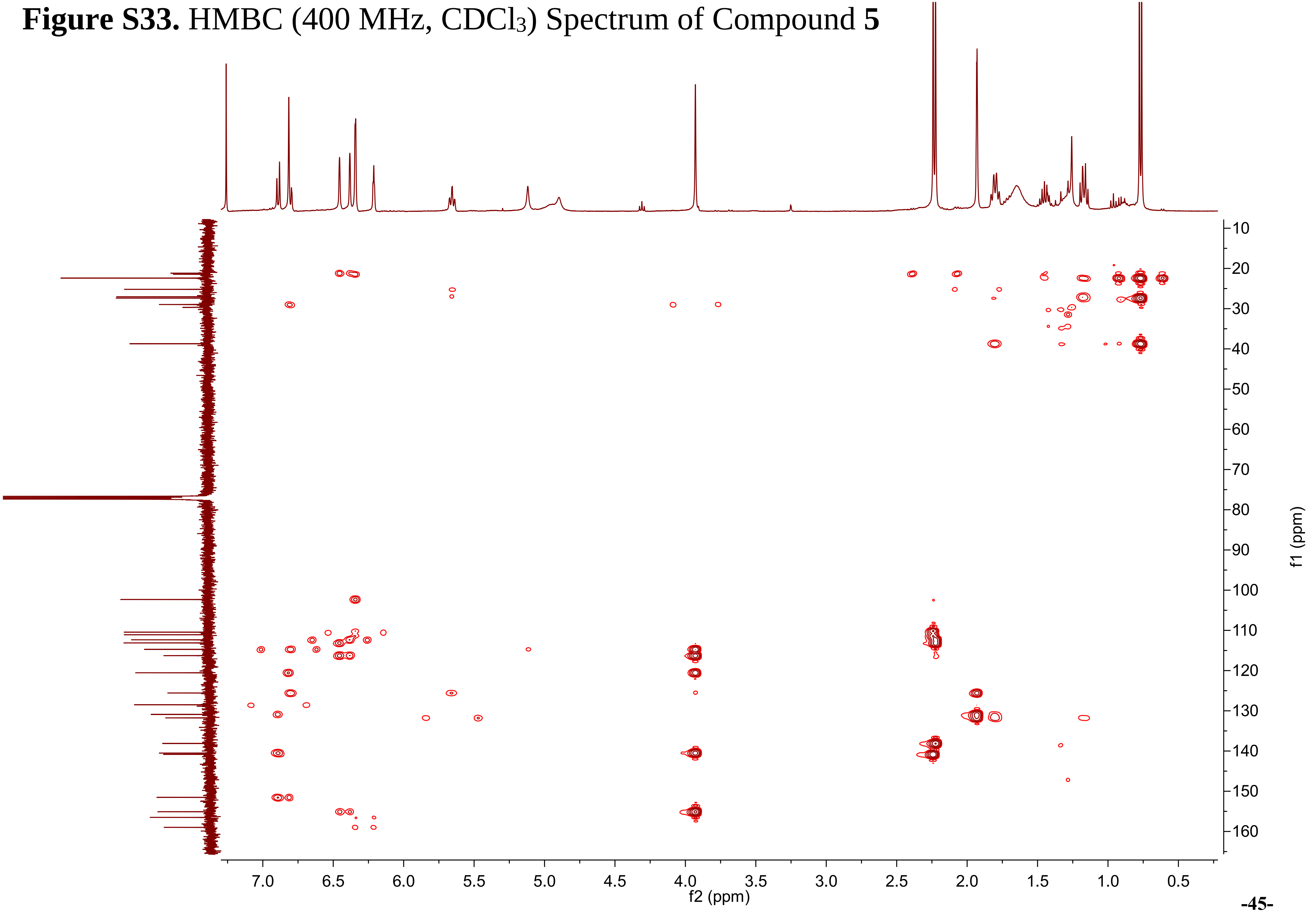

Figure S34. 1H NMR (400 MHz, $CDCl_3$) Spectrum of Compound **6**

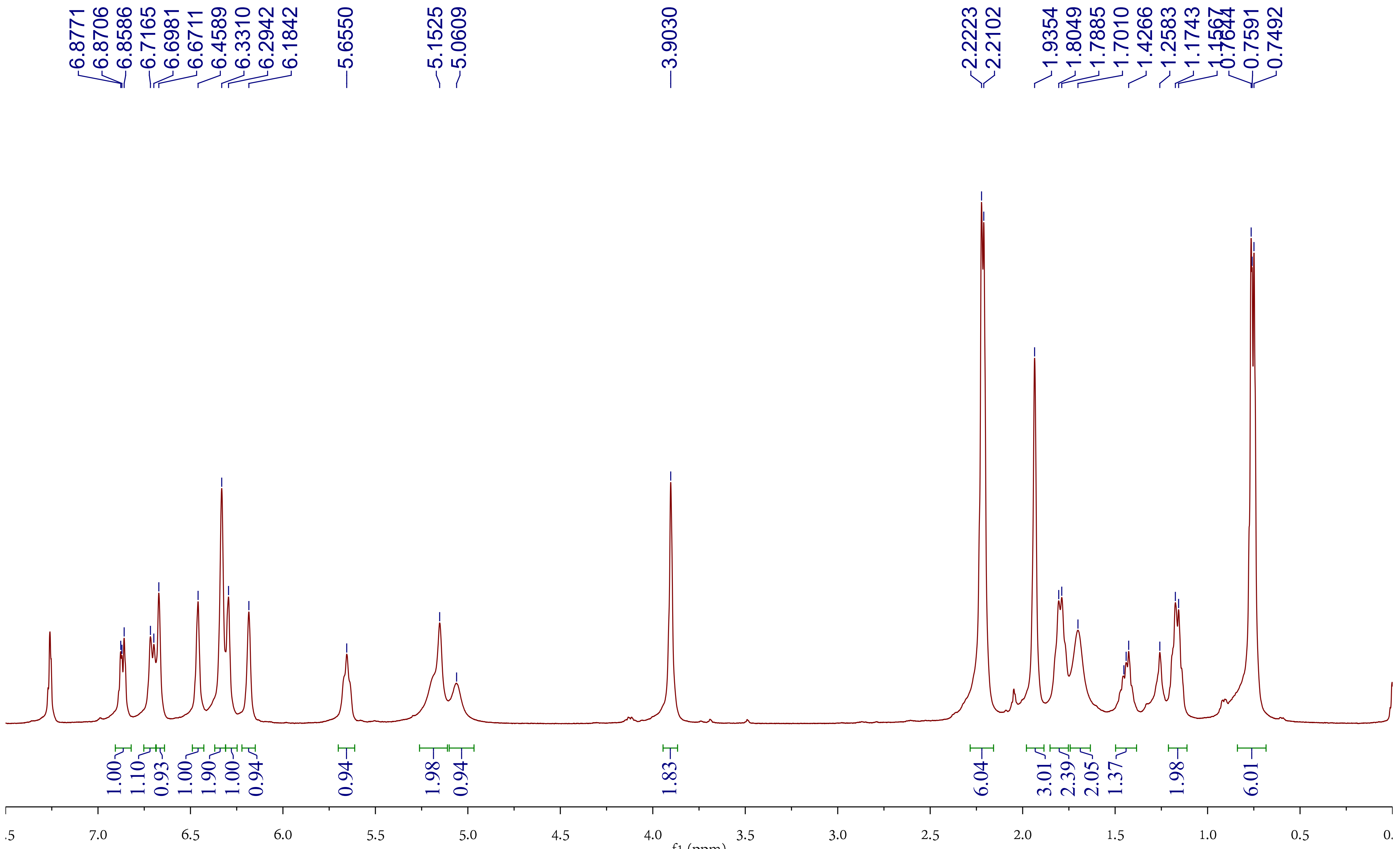

Figure S35. ^{13}C NMR (100 MHz, $CDCl_3$) Spectrum of Compound **6**

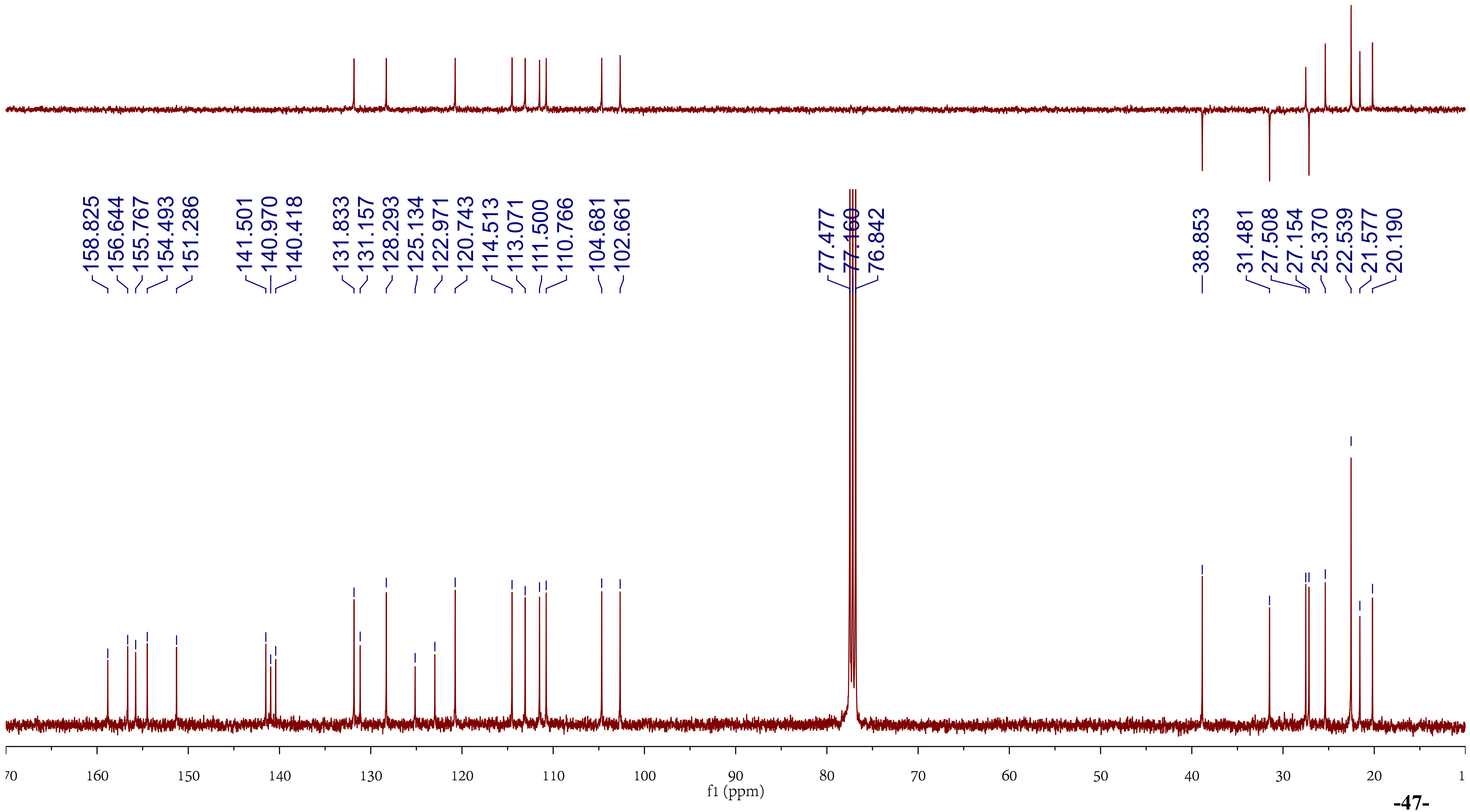

Figure S36. ^{1}H NMR (400 MHz, $CDCl_3$) Spectrum of Compound **7**

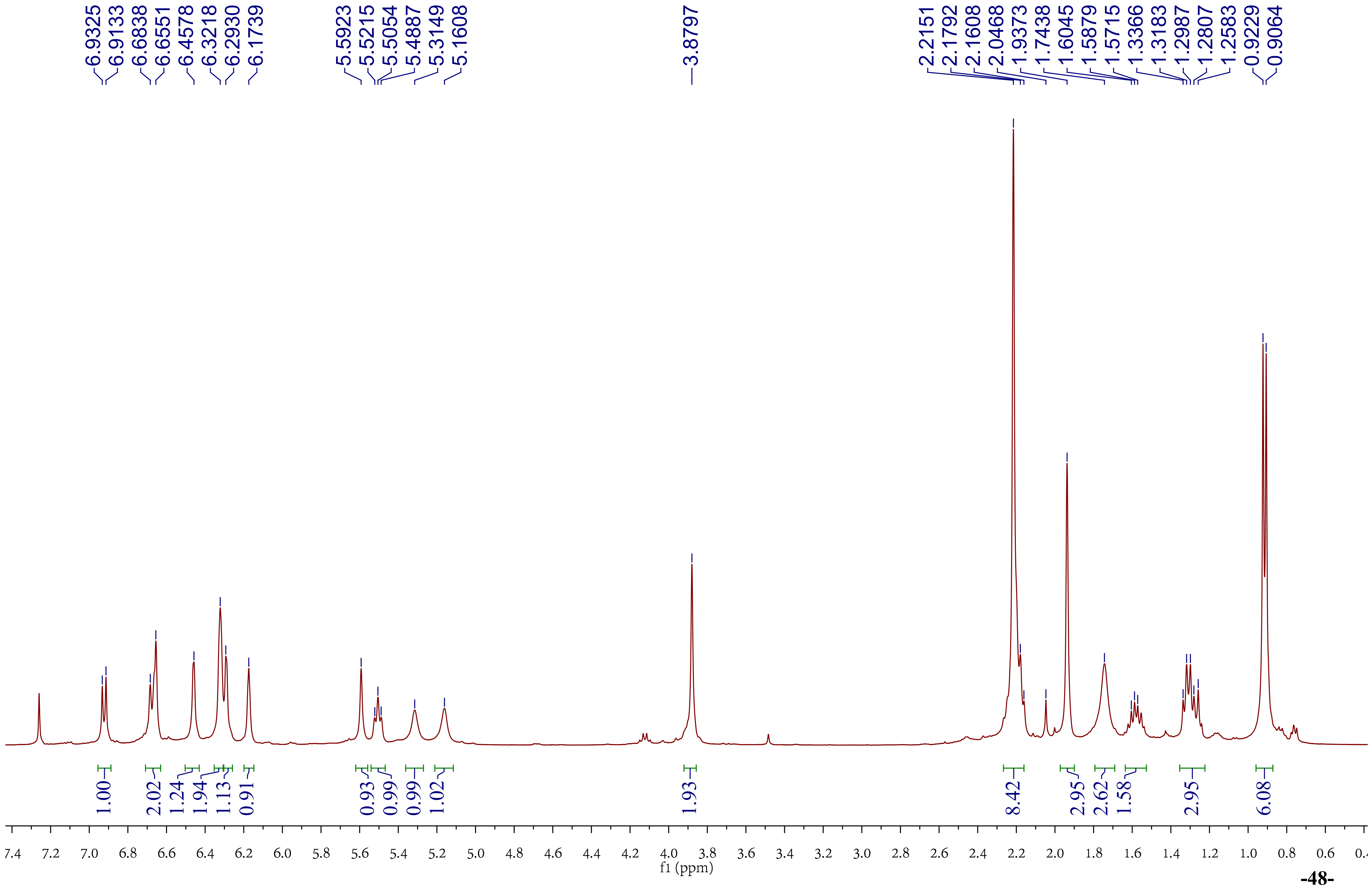

Figure S37.^{13}C NMR (100 MHz, $CDCl_3$) Spectrum of Compound **7**

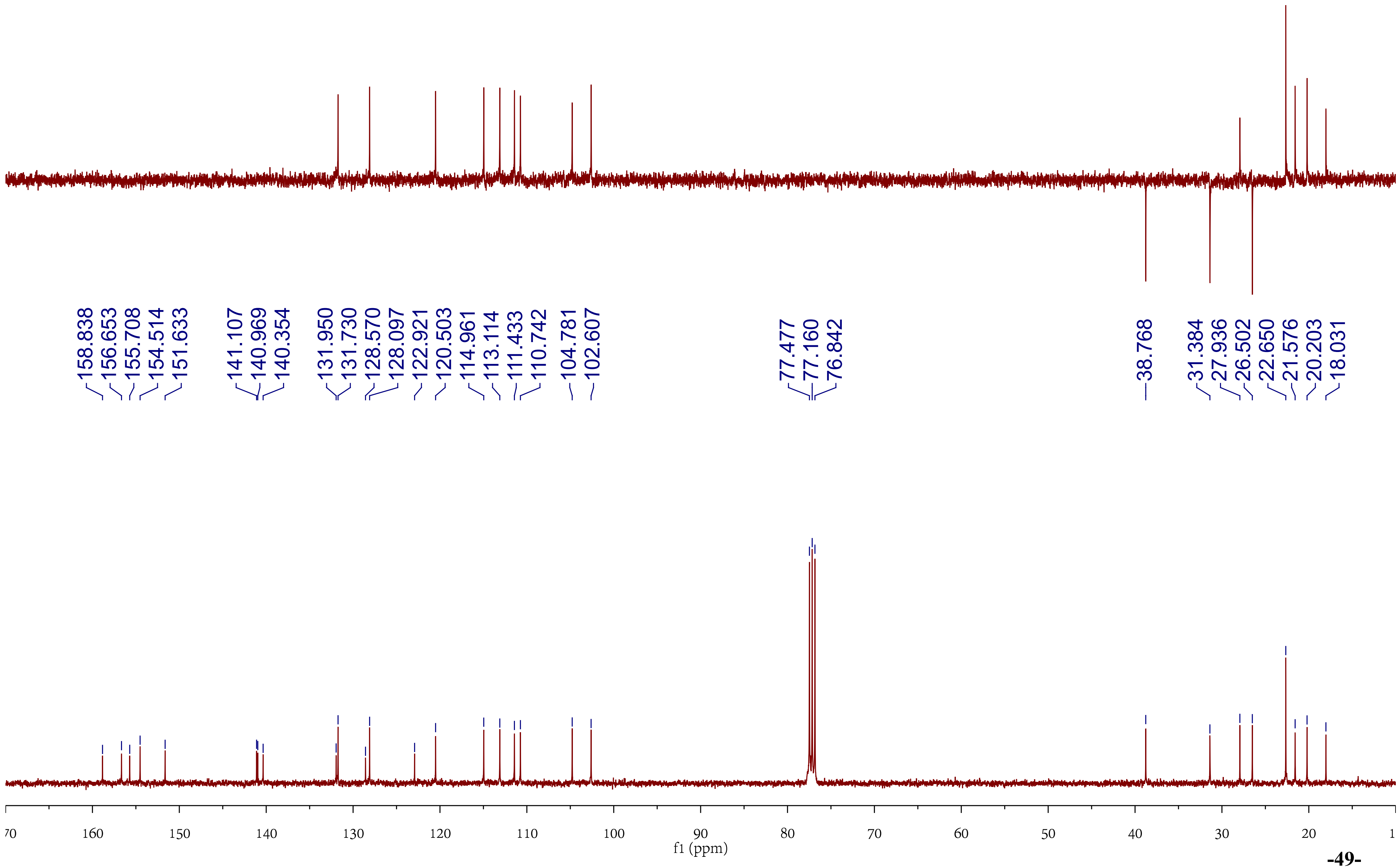

Figure S38. ^{1}H NMR (400 MHz, $CDCl_3$) Spectrum of Compound **8**

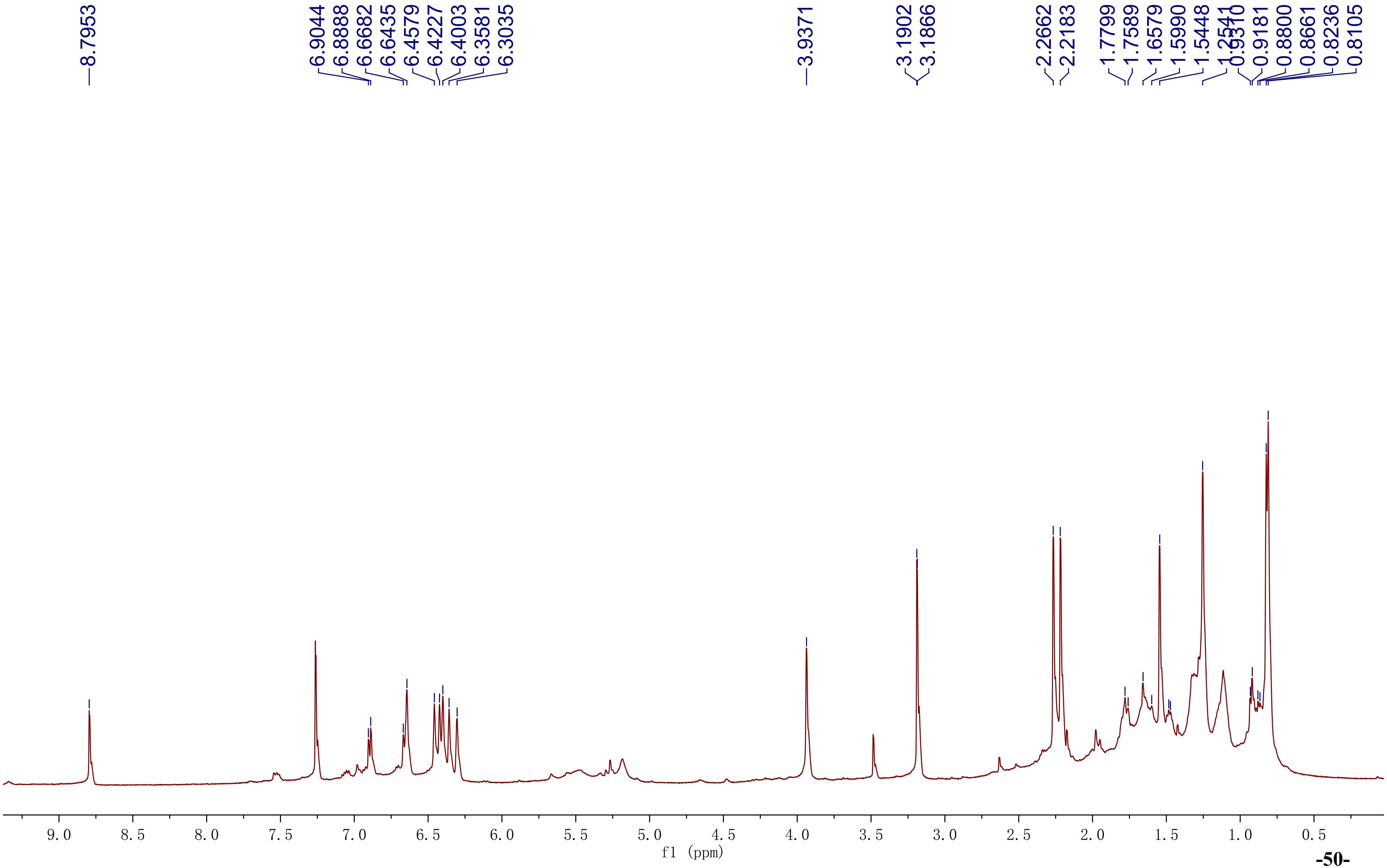

Figure S39. ^{13}C NMR (100 MHz, $CDCl_3$) Spectrum of Compound **8**

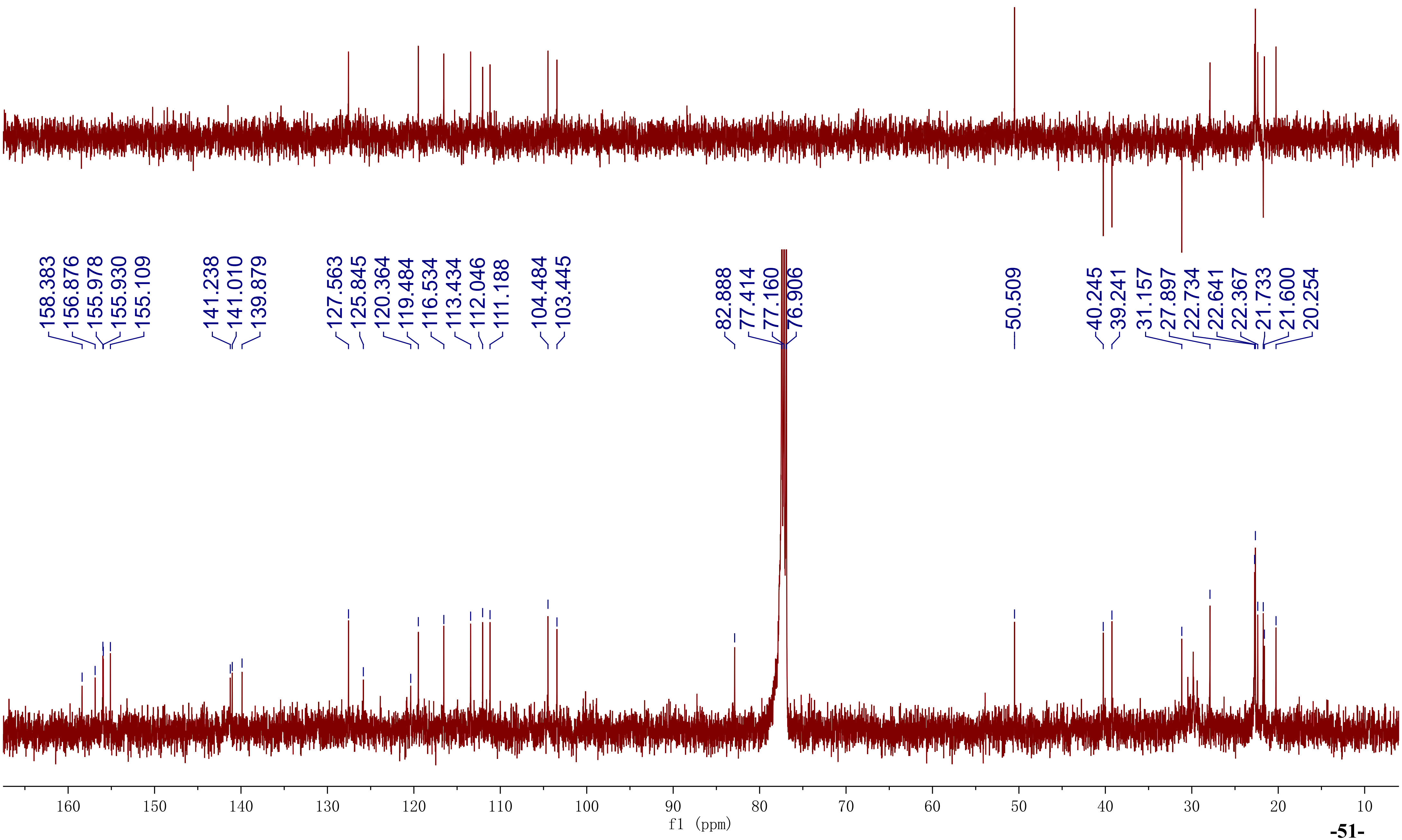

Figure S40. HSQC (400 MHz, $CDCl_3$) Spectrum of Compound **8**

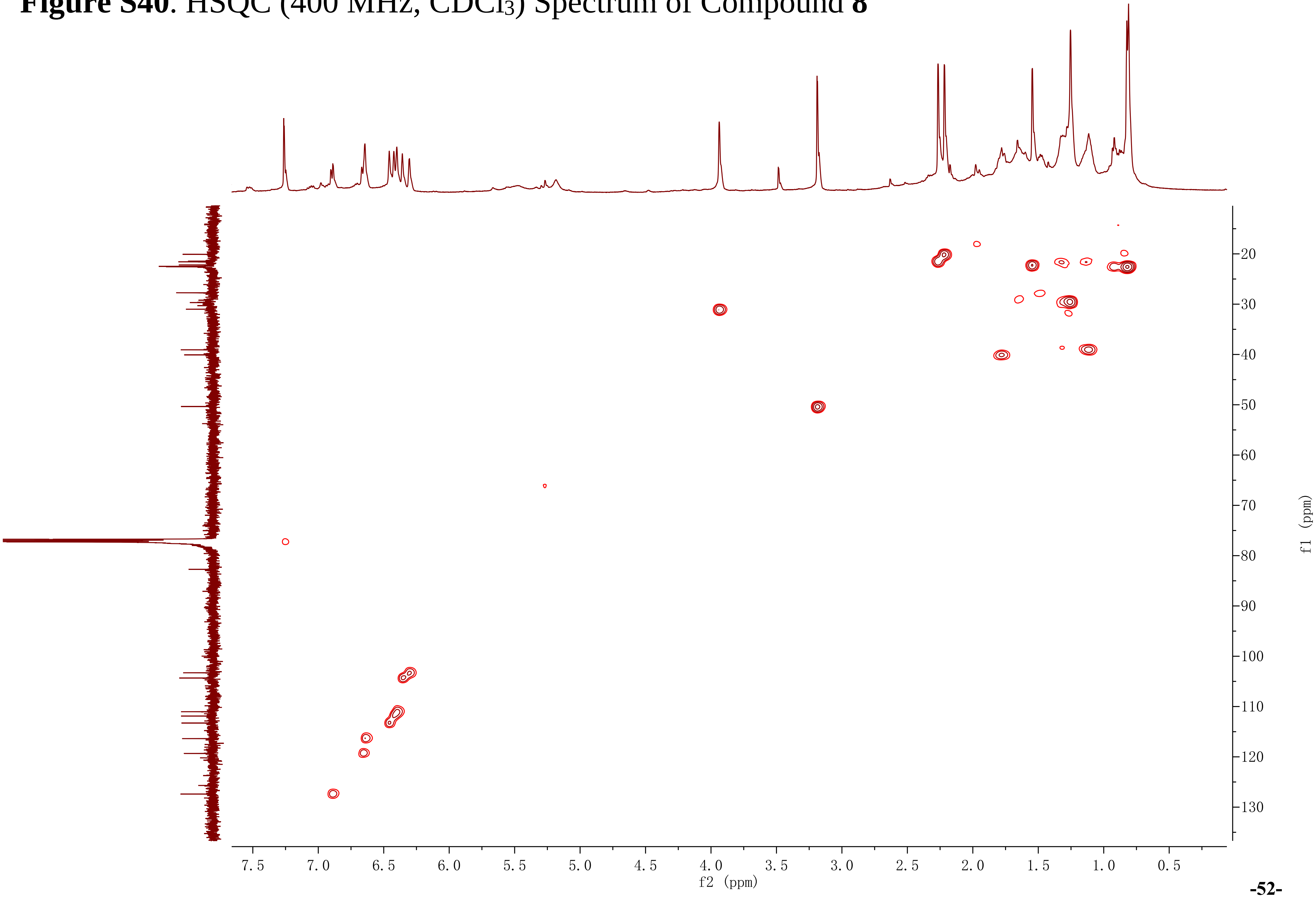

Figure S41. ^1H-^1H COSY (400 MHz, $CDCl_3$) Spectrum of Compound **8**

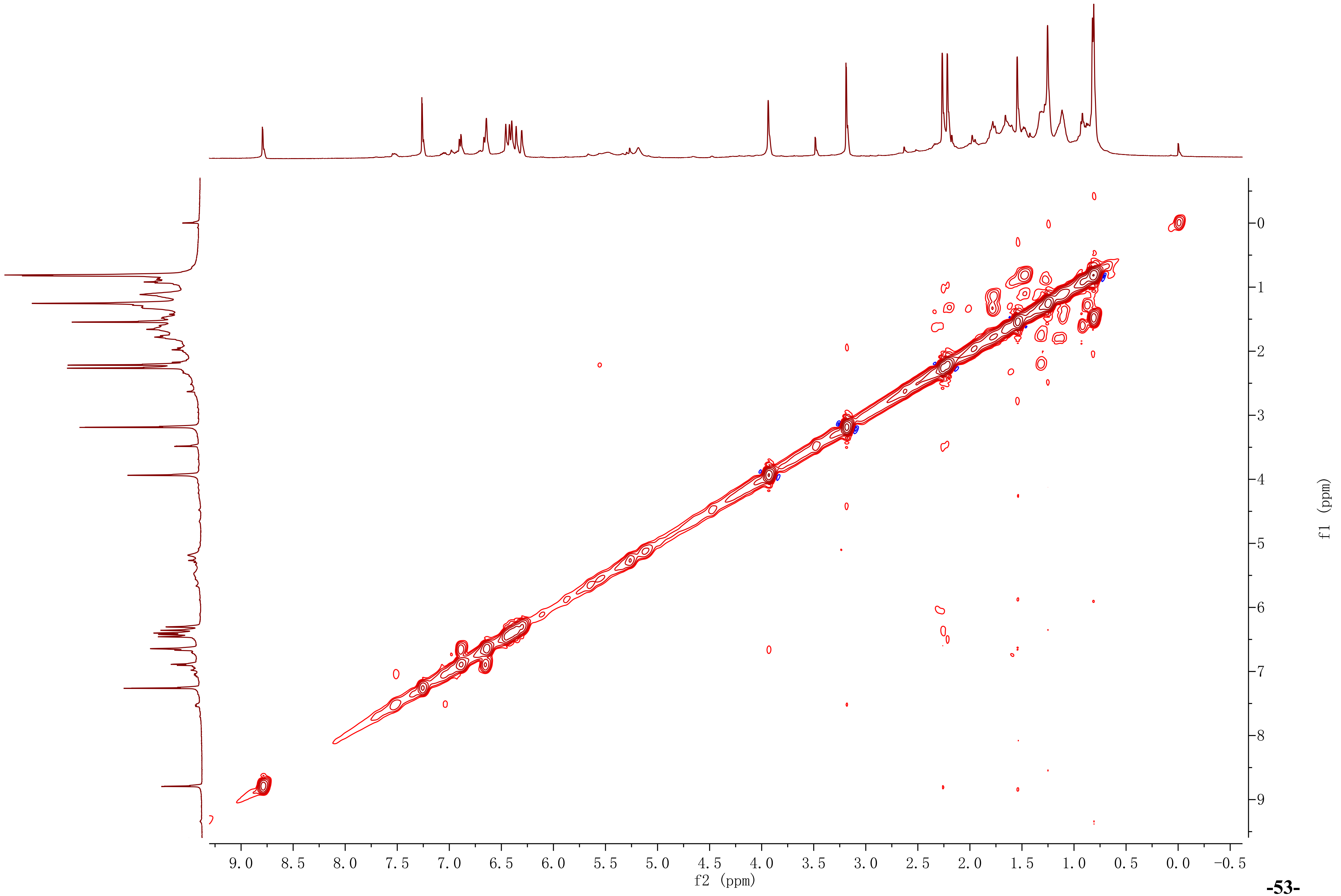

Figure S42. HMBC (400 MHz, $CDCl_3$) Spectrum of Compound **8**

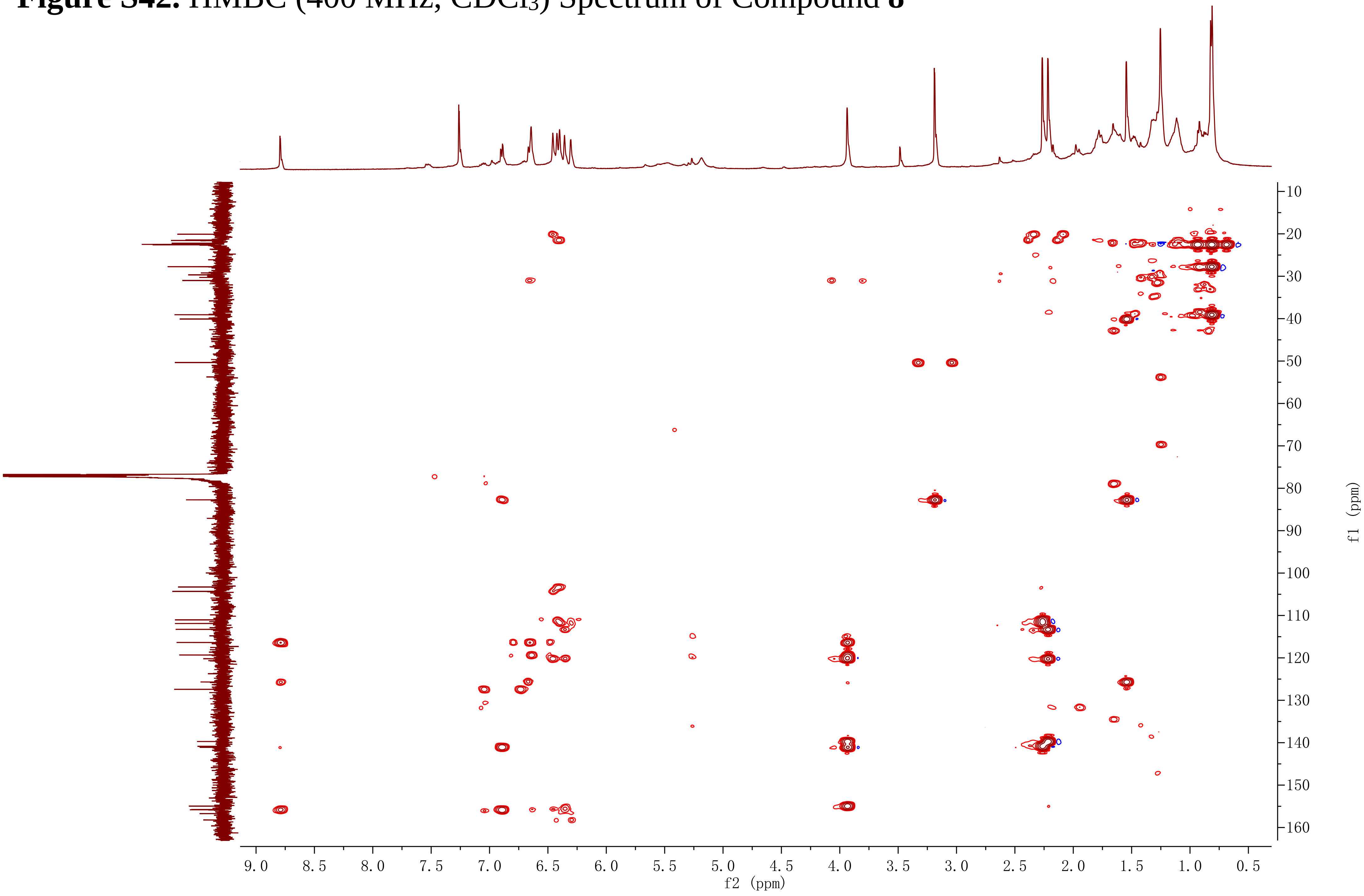

Figure S43. ^{1}H NMR (500MHz, $CDCl_3$) Spectrum of Compound **9**

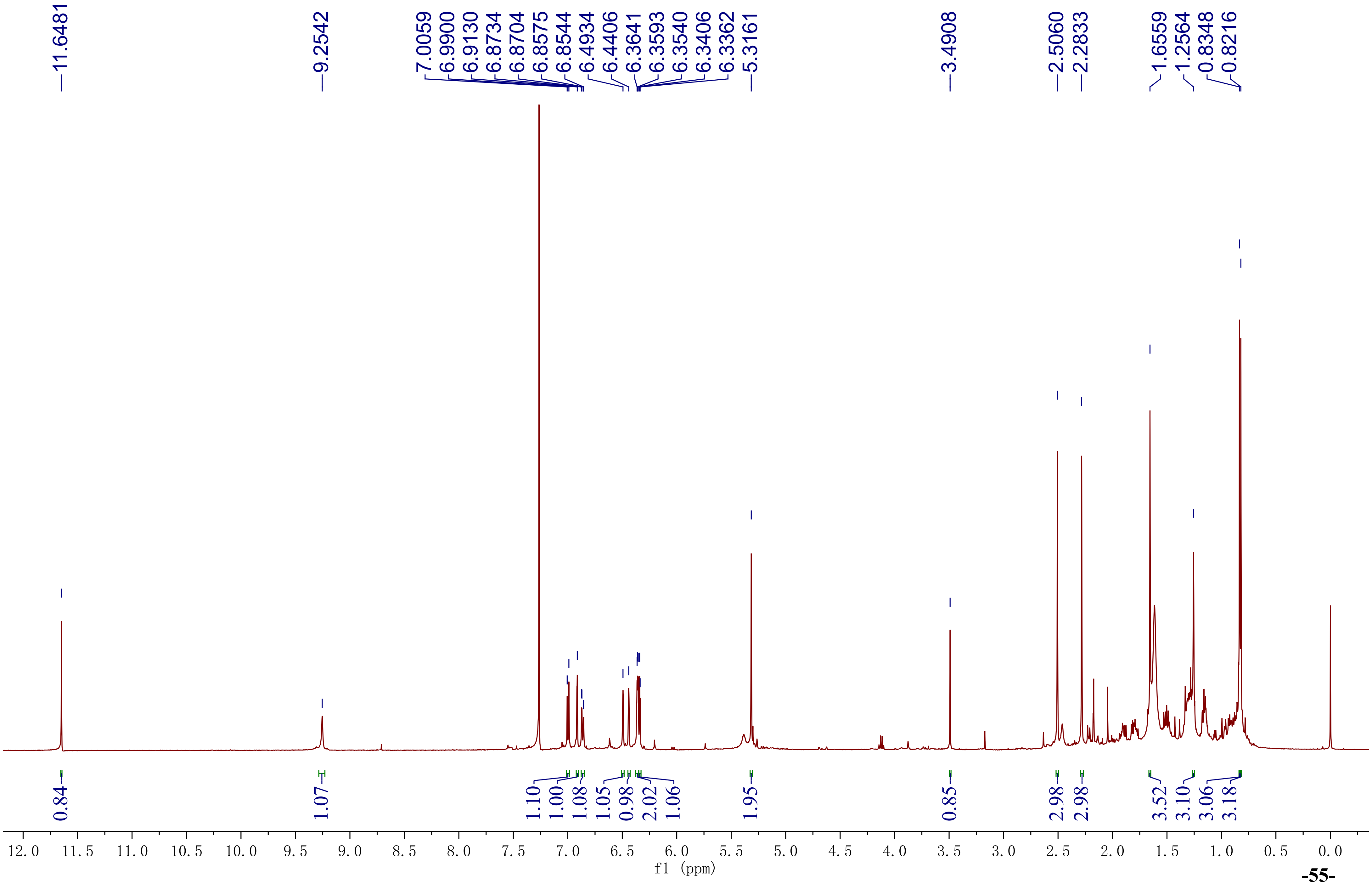

Figure S44. ^{13}C NMR (125 MHz, $CDCl_3$) Spectrum of Compound **9**

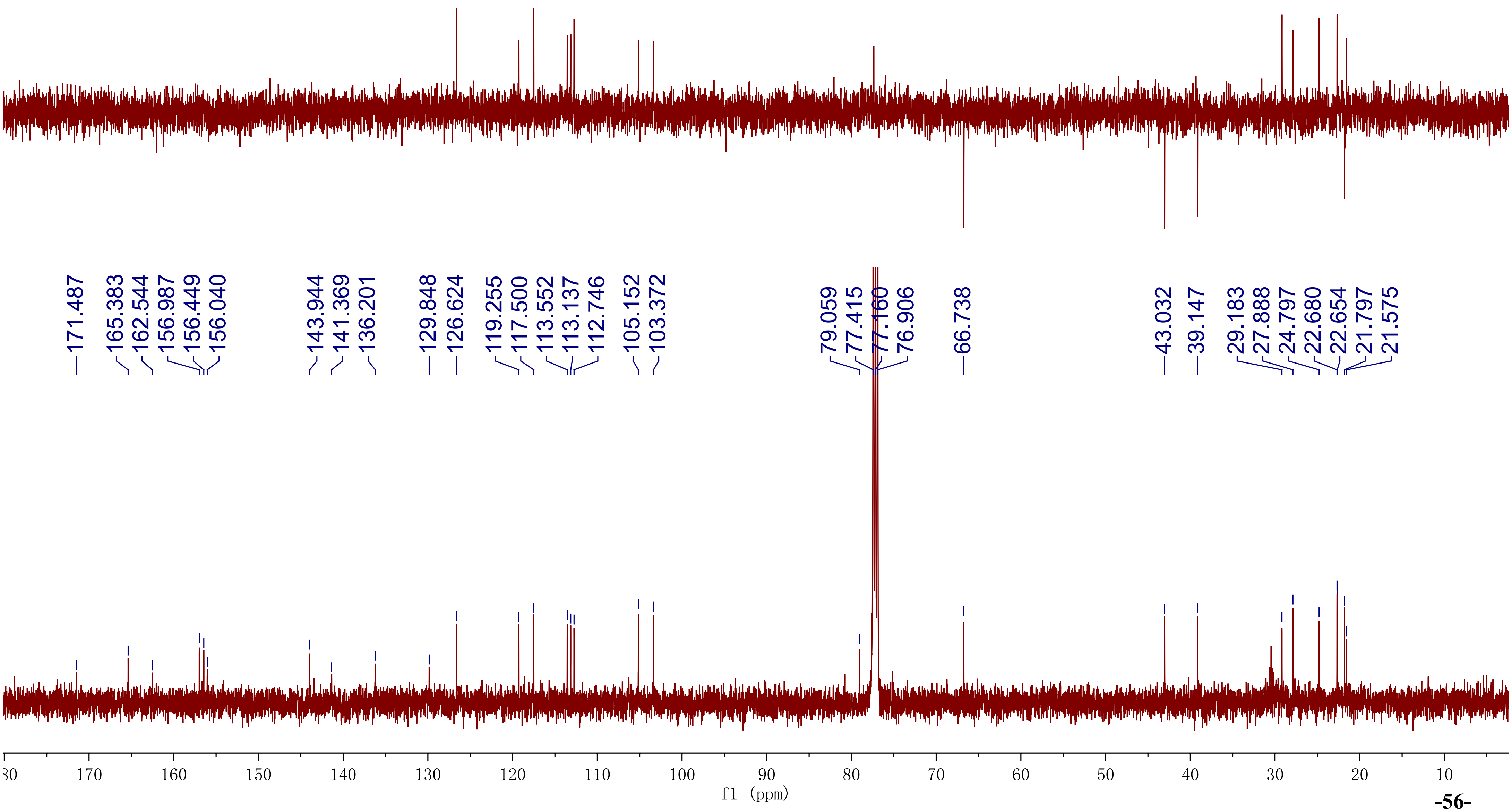

FigureS45.^{1}H NMR (400 MHz, $CDCl_3$) Spectrum of Compound **10**

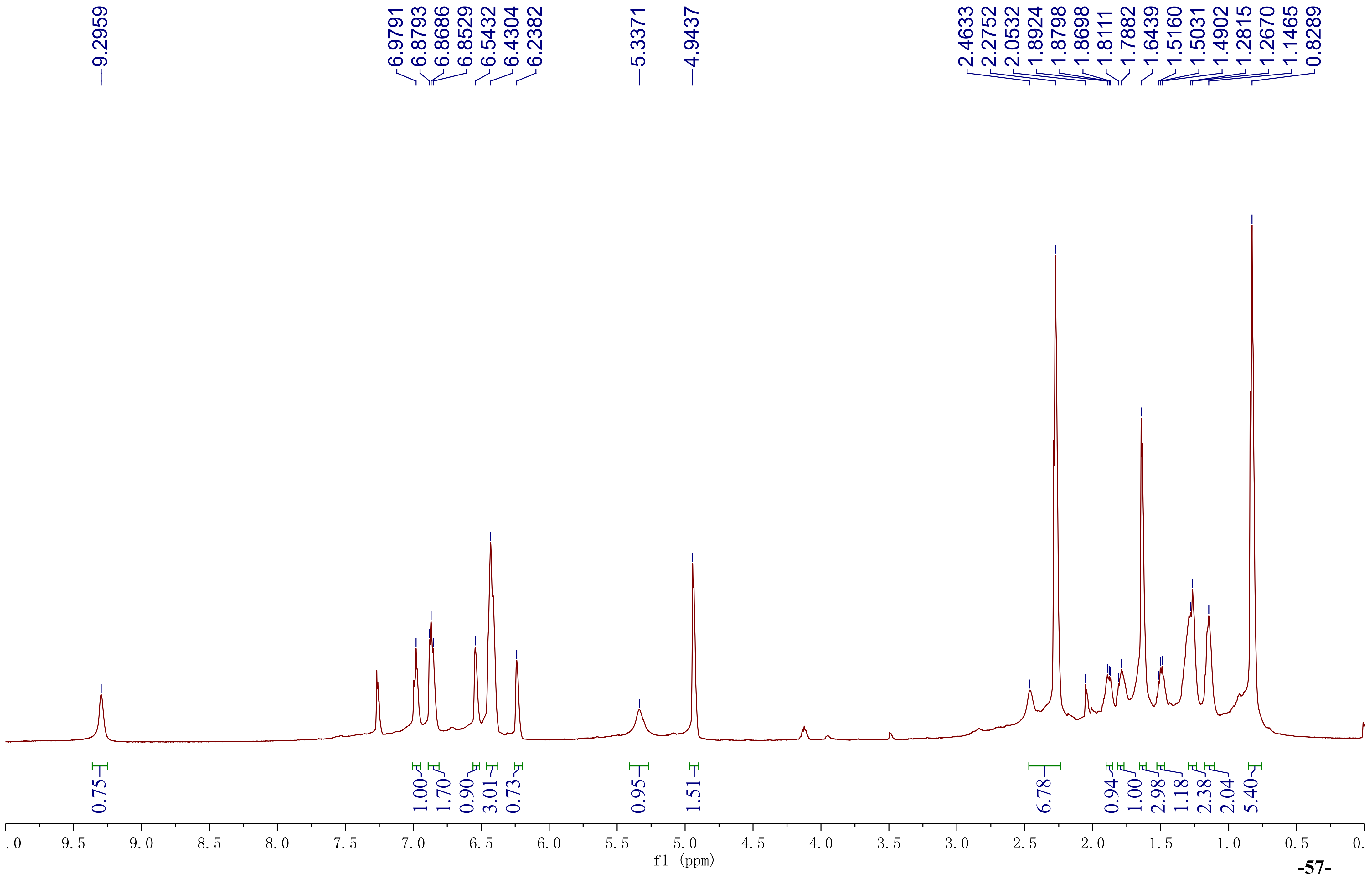

Figure S46. ^{13}C NMR (100 MHz, $CDCl_3$) Spectrum of Compound **10**

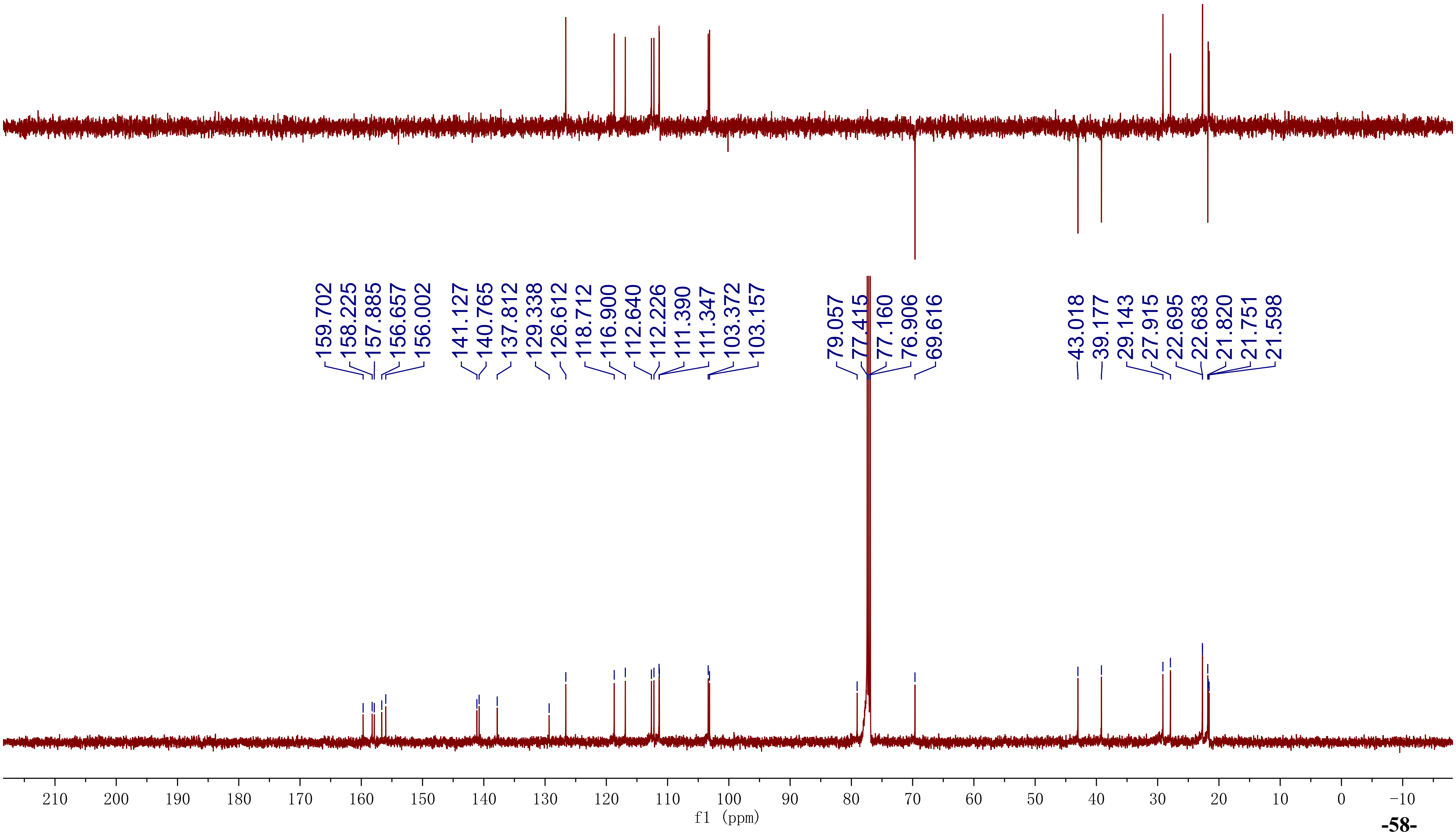

Figure S47. ^{1}H NMR (400 MHz, Acetone-d_6) Spectrum of Compound **11**

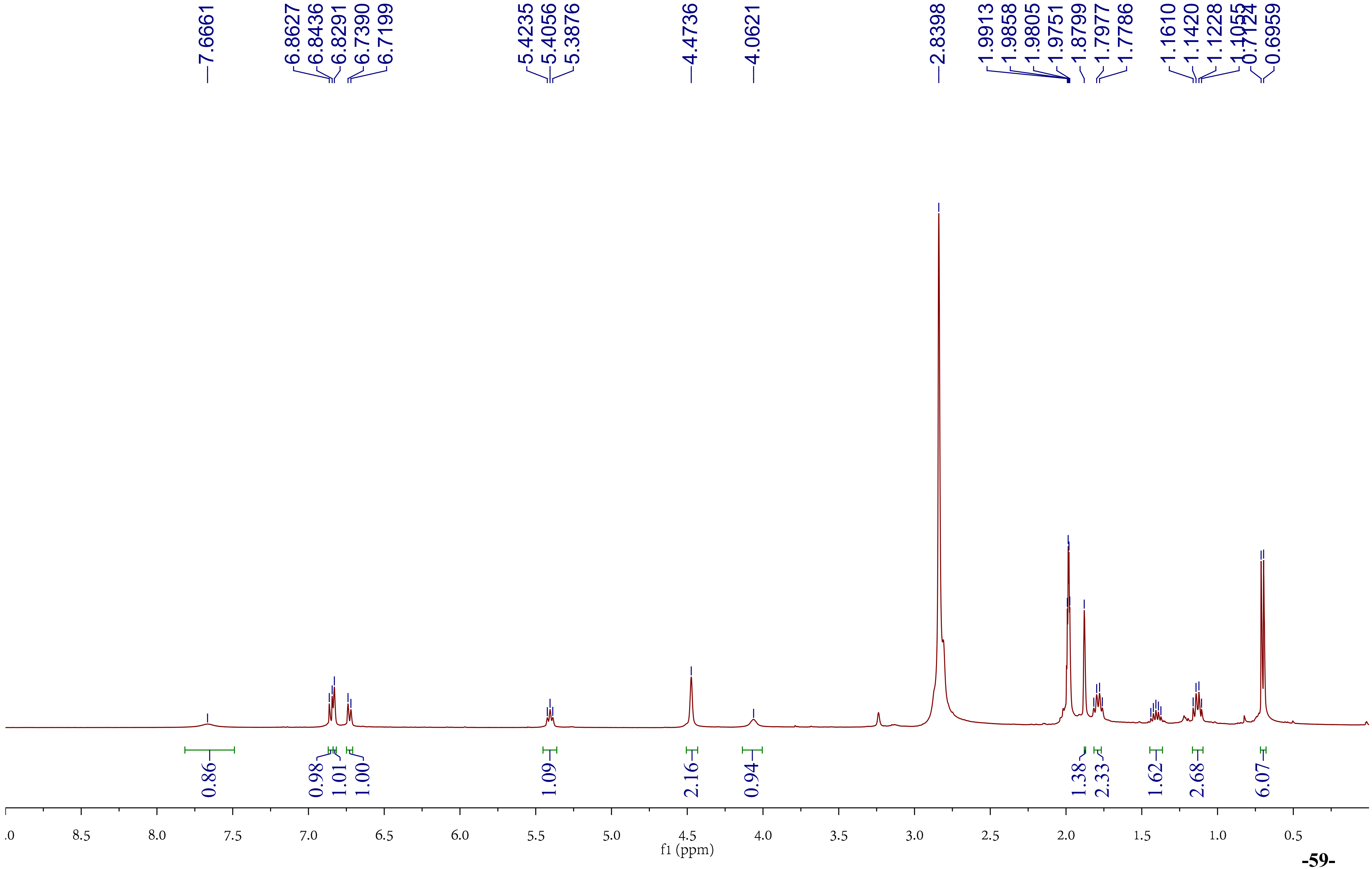

Figure S48. ^{13}C NMR (100 MHz, Acetone-d_6) Spectrum of Compound **11**

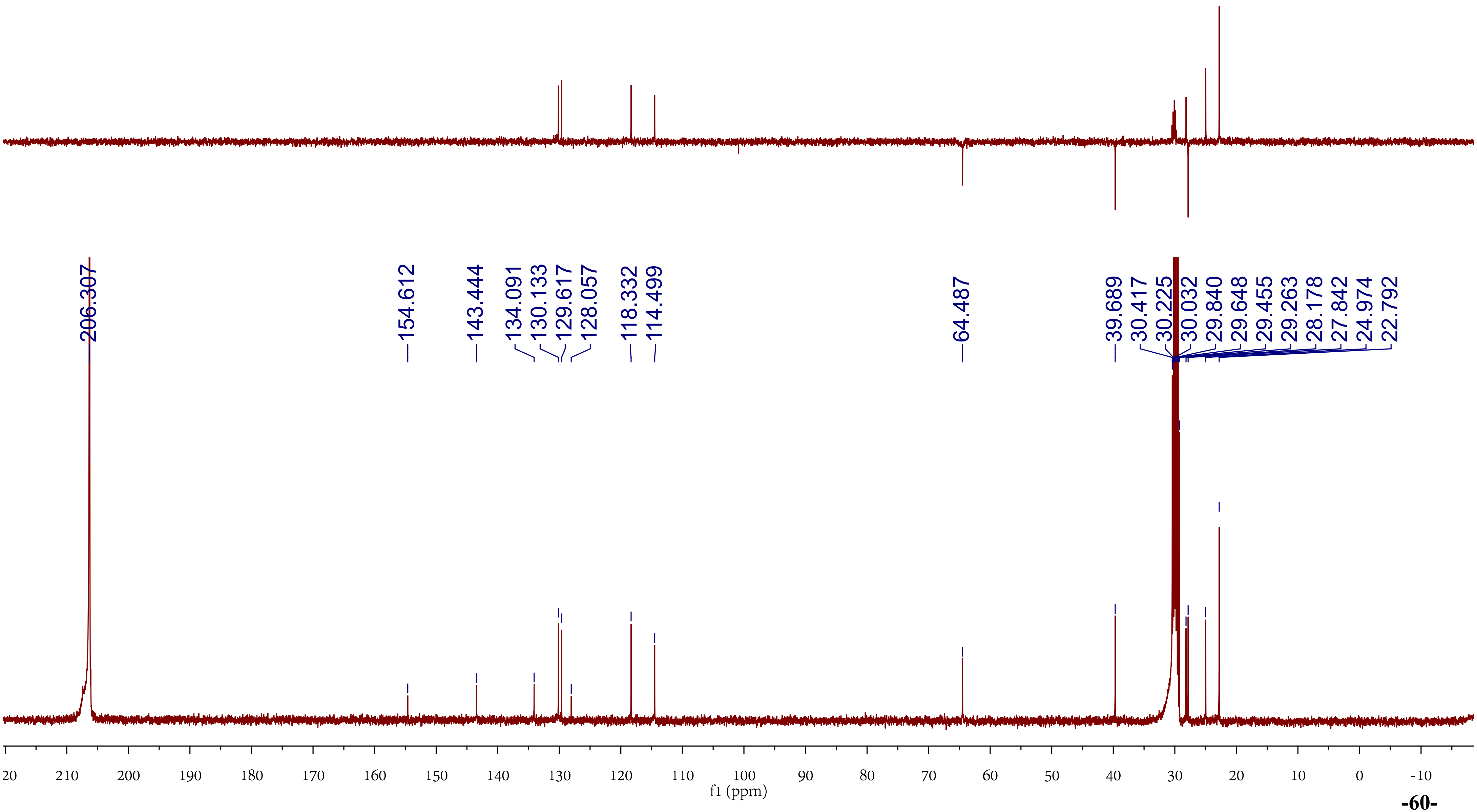

Figure S49. ^{1}H NMR (400 MHz, $CDCl_3$) Spectrum of Compound **12**

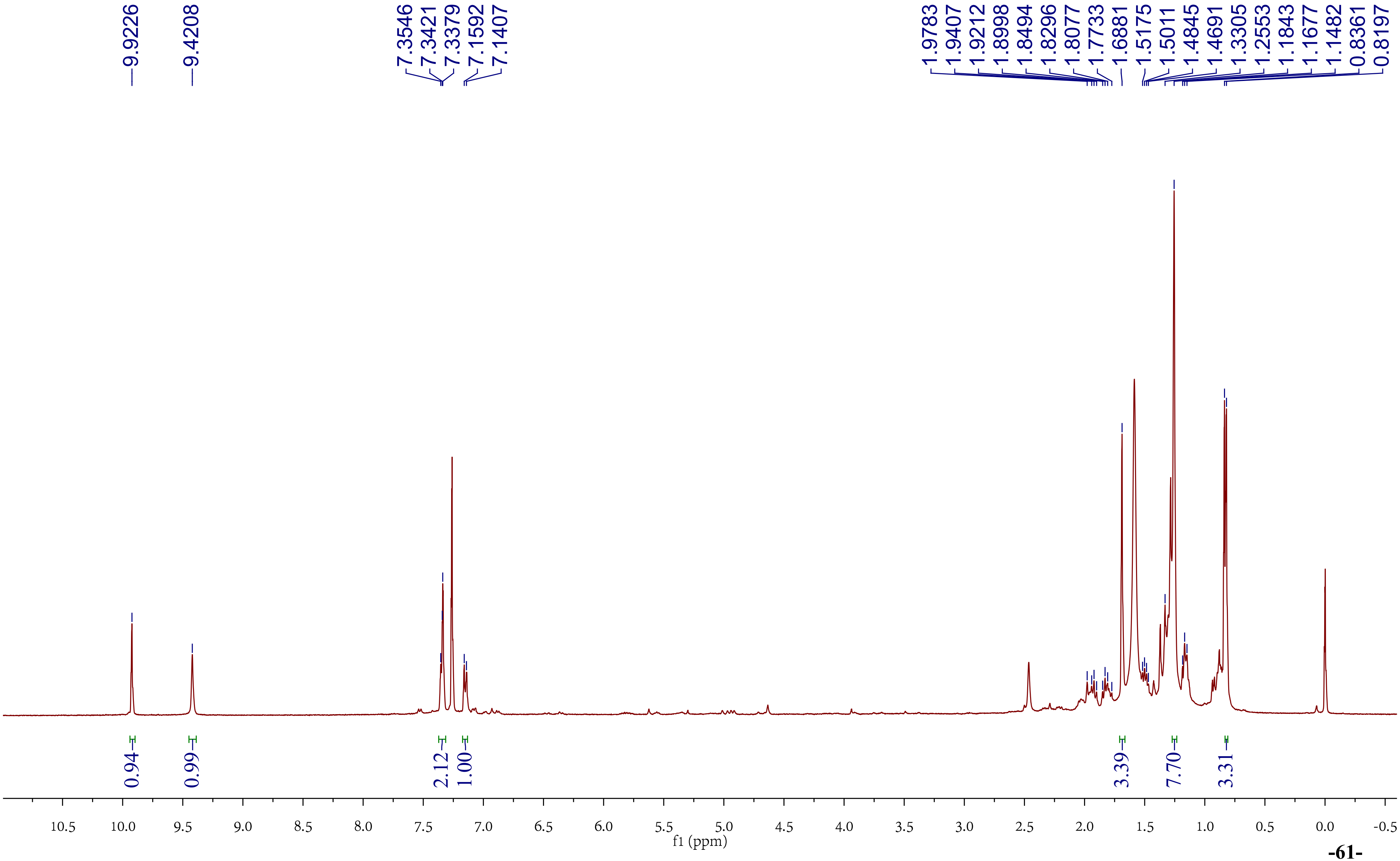

Figure S50.^{13}C NMR (100 MHz, $CDCl_3$) Spectrum of Compound **12**

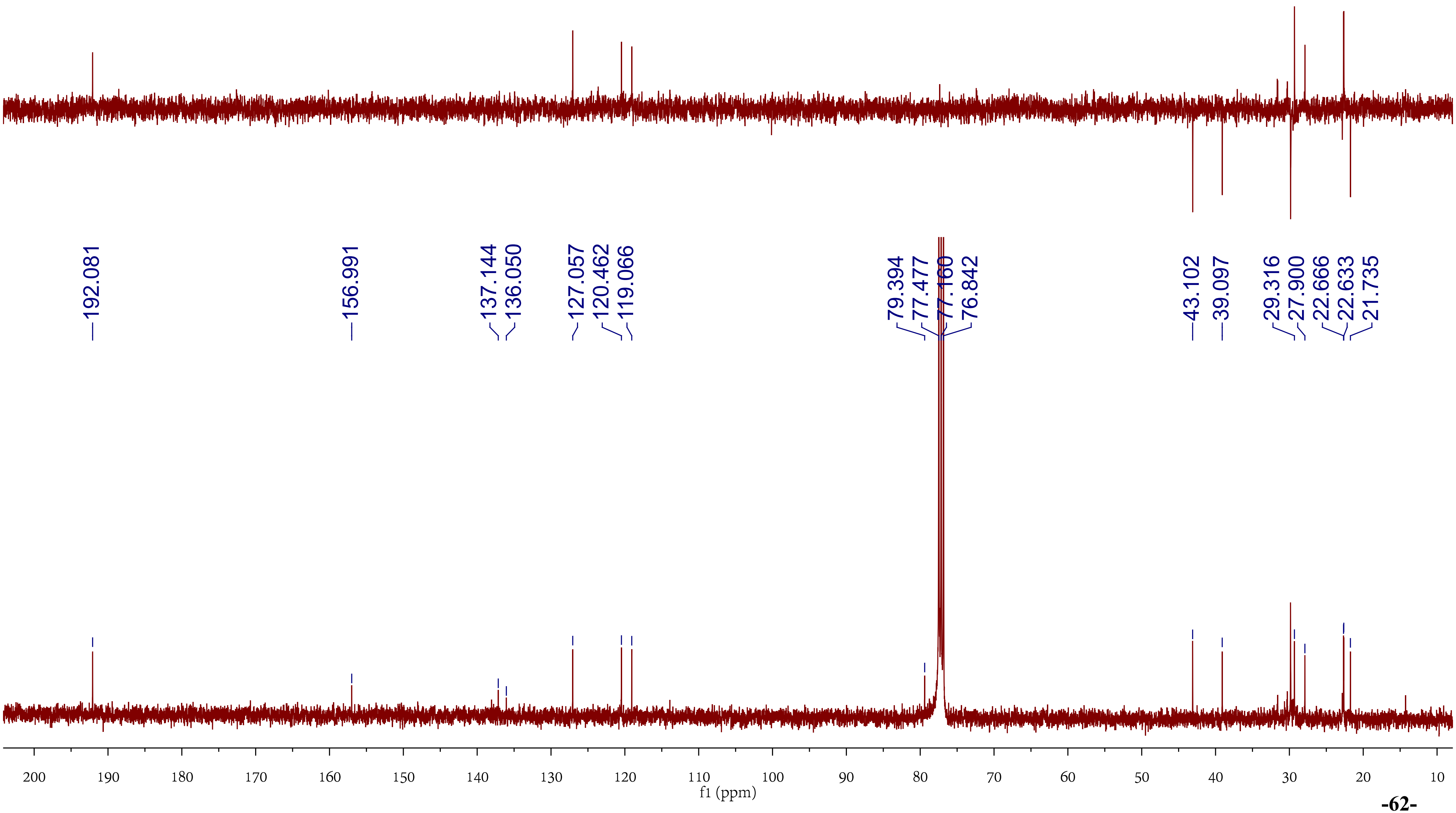

Figure S51. ^{1}H NMR (500 MHz, Acetone-d_6) Spectrum of Compound **13**

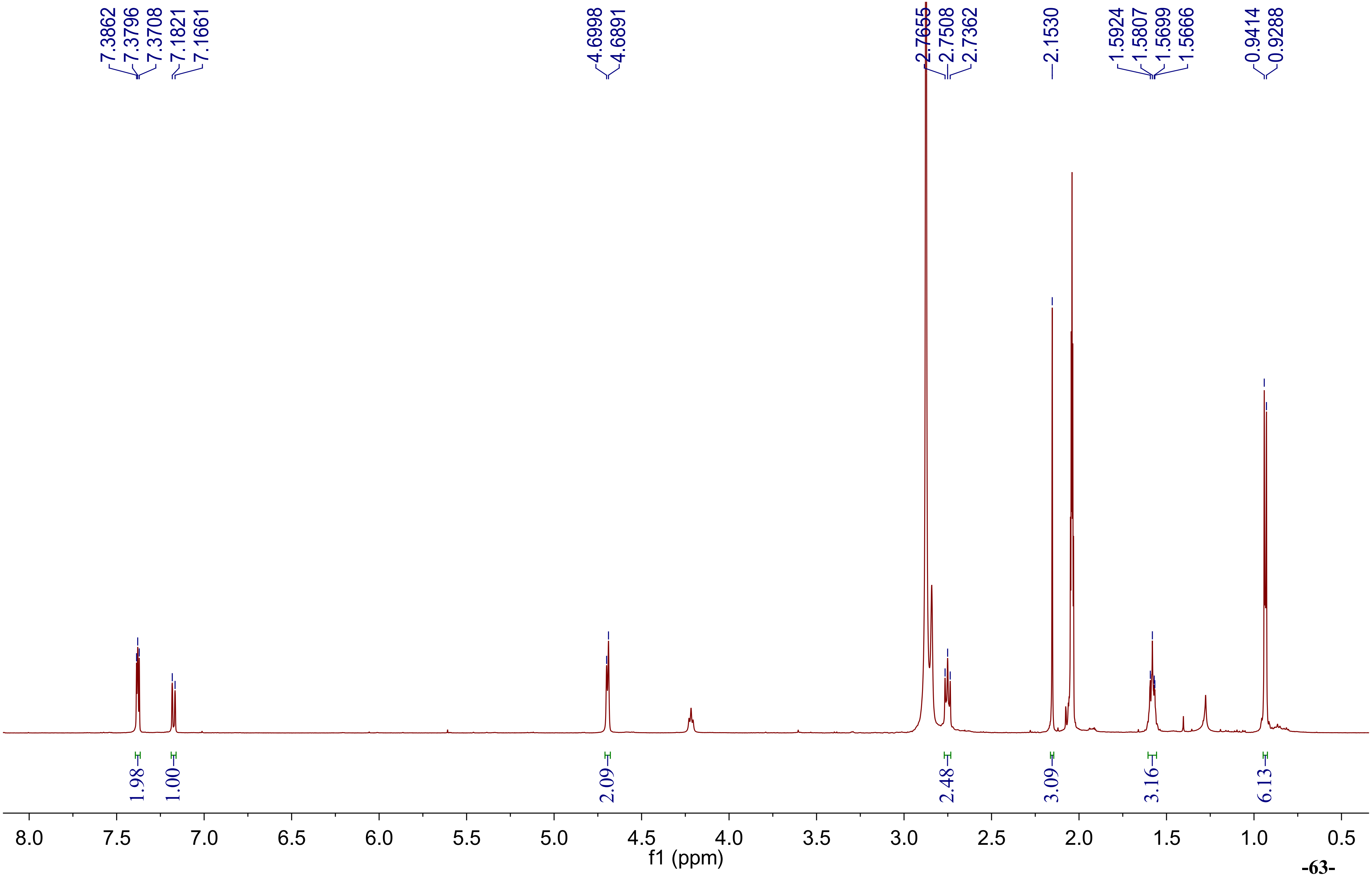

Figure S52. ^{13}C NMR (125 MHz, Acetone-d_6) Spectrum of Compound **13**

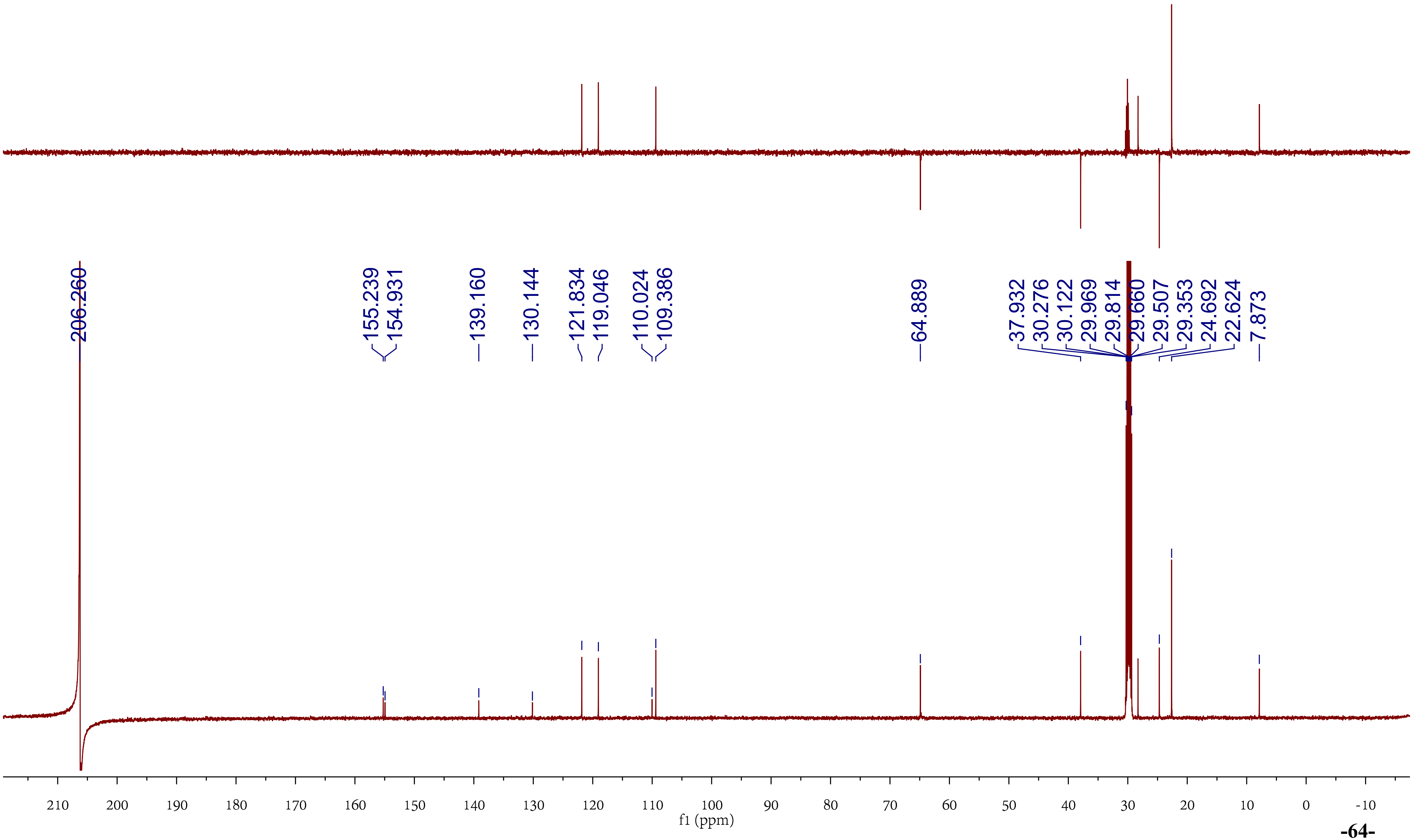

Figure S53. ^{1}H NMR (500 MHz, Acetone-d_6) Spectrum of Compound **14**

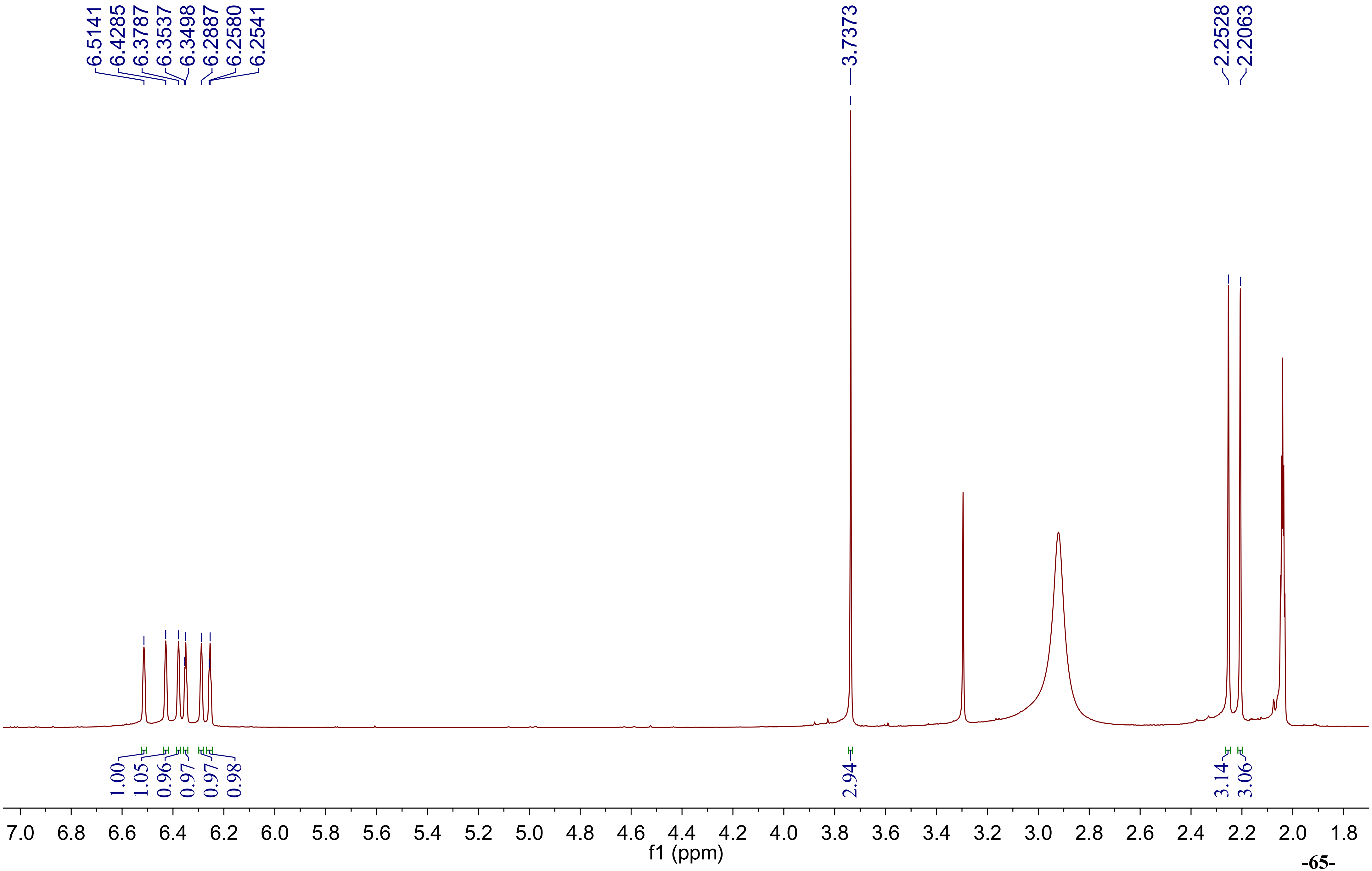

Figure S54. ^{13}C NMR (125 MHz, Acetone-d_6) Spectrum of Compound **14**

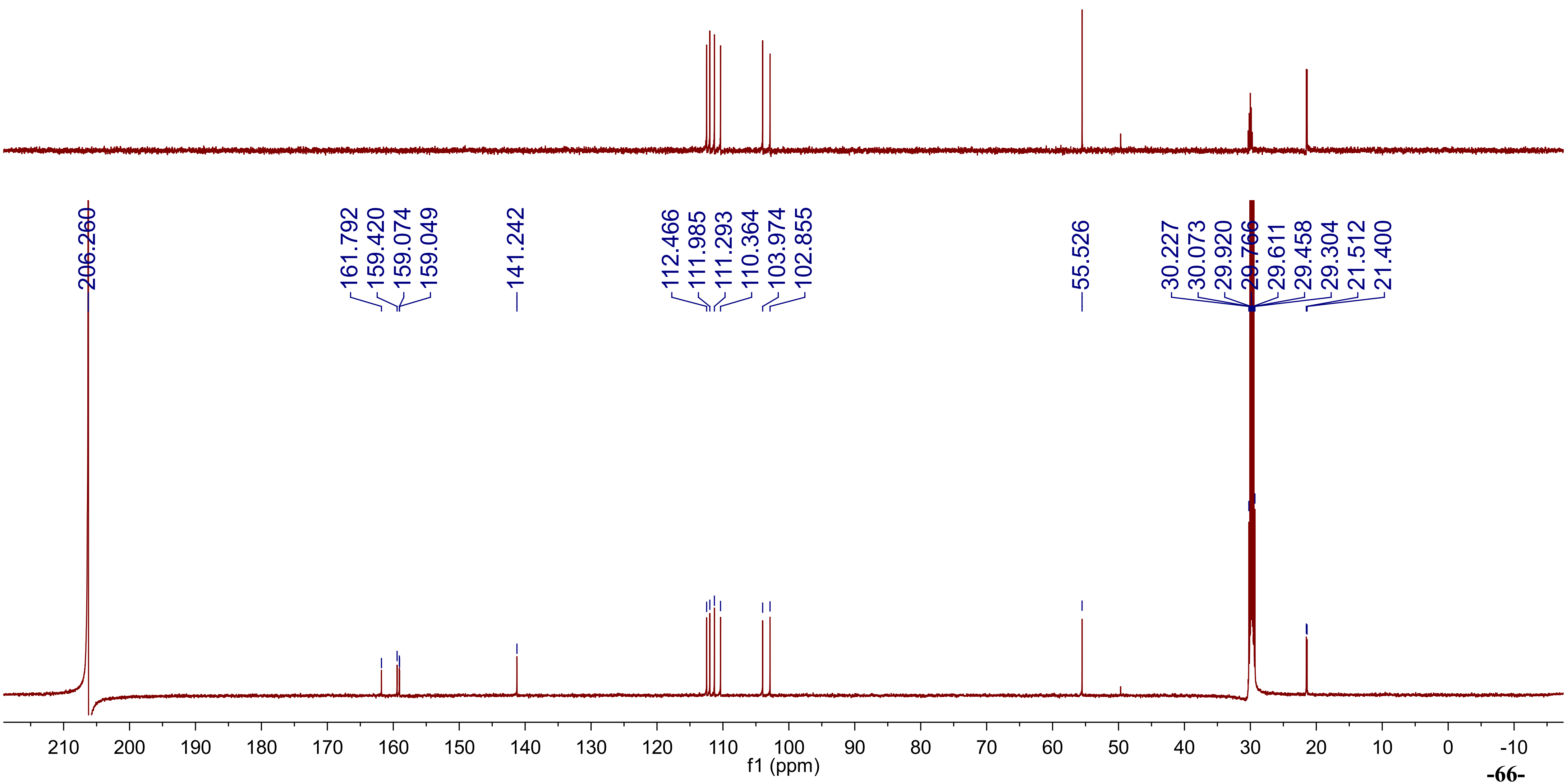

Figure S55. ^{1}H NMR (400 MHz, $CDCl_3$) Spectrum of Compound **15**

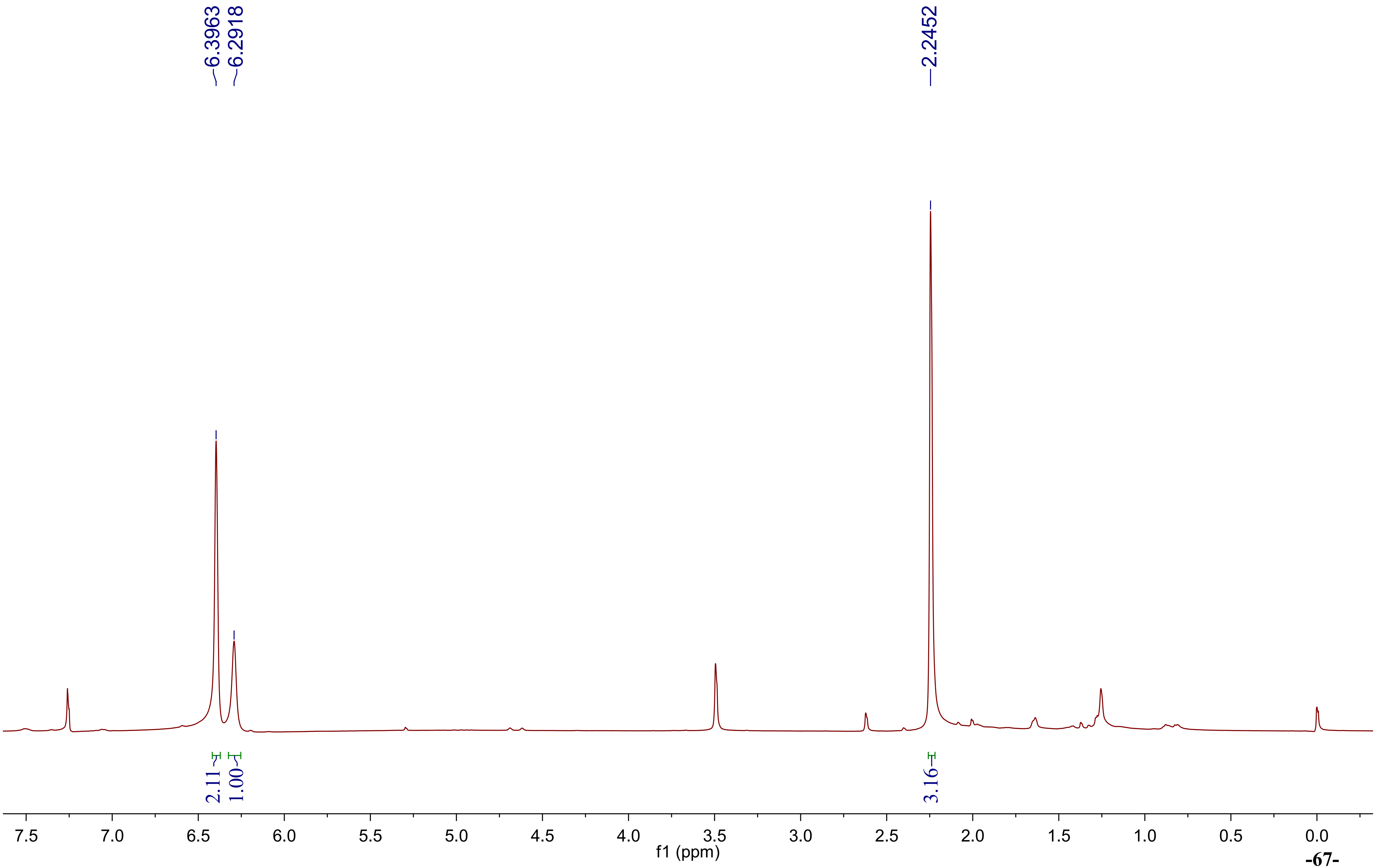

Figure S56. ^{13}C NMR (100 MHz, $CDCl_3$) Spectrum of Compound **15**

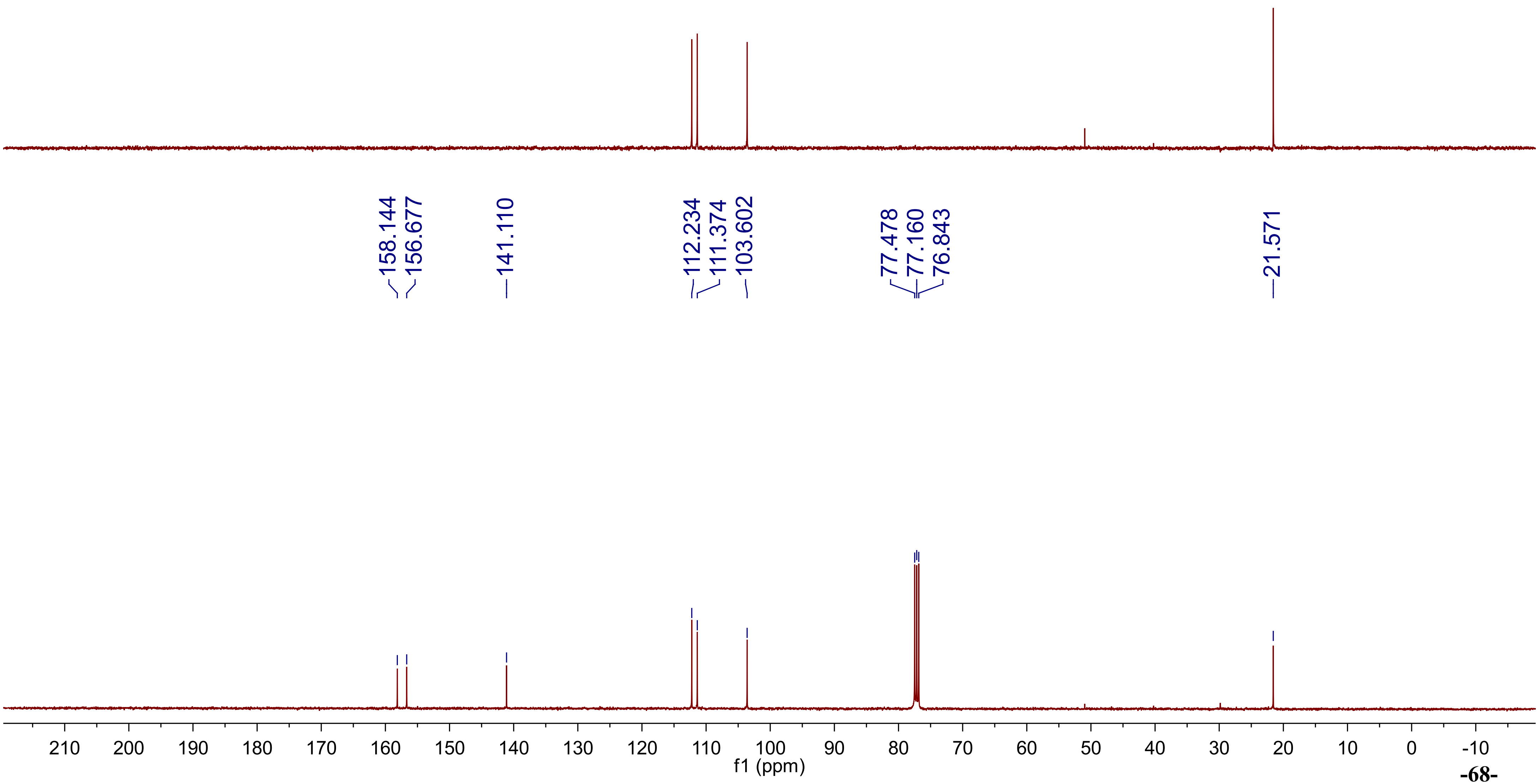

Figure S57. B3LYP/6-31G(d) optimized lowest energy conformers for compound **1a**

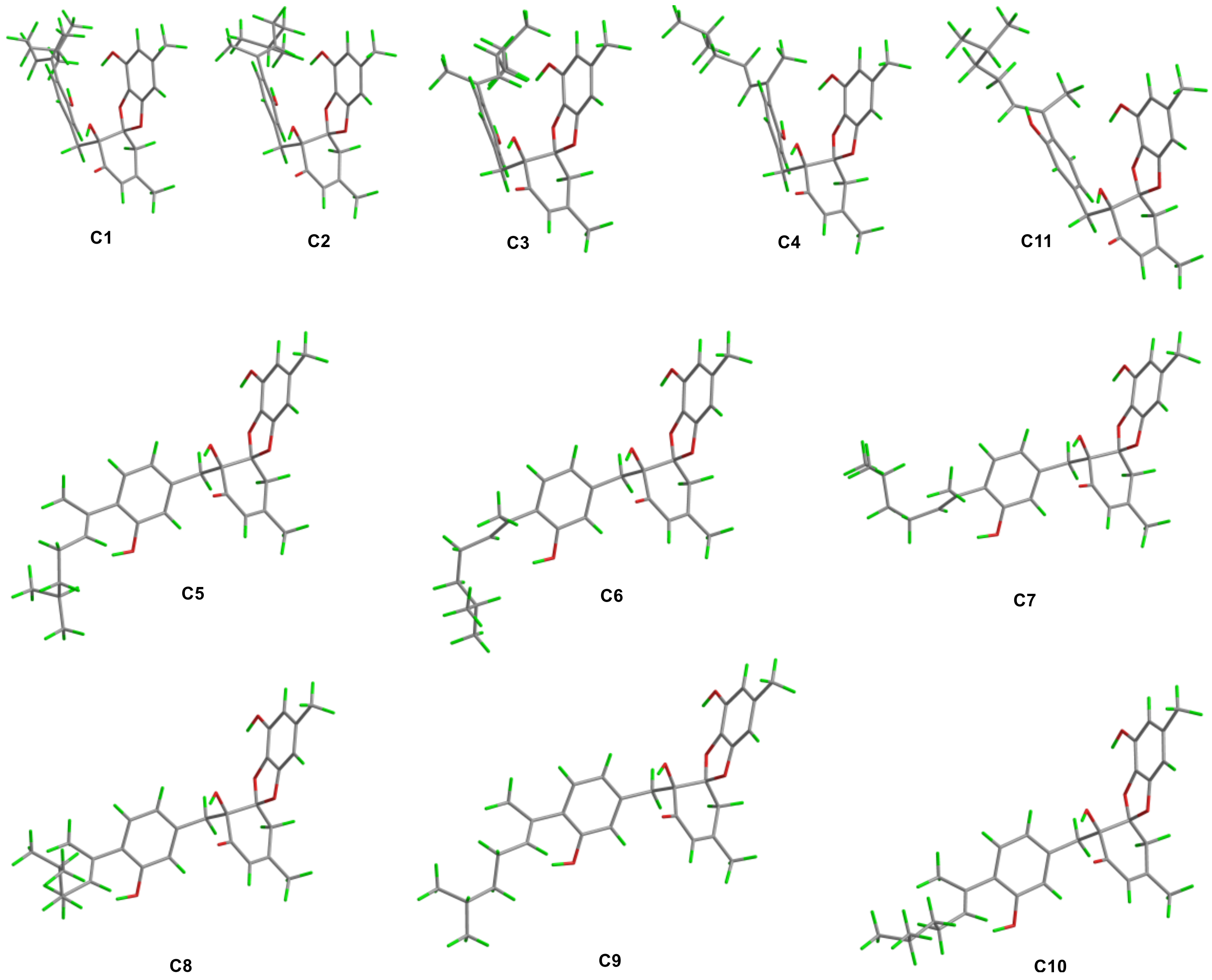

Table S11. Energy (298.15 K) analysis for **1a**

Conf.	G (Hartree)	ΔG (Kcal/mol)	Boltzmann Distribution
C1	-1576.36819	1.32781116	0.022361901
C2	-1576.36916	0.71912646	0.062503648
C3	-1576.36917	0.71285136	0.063169494
C5	-1576.368522	1.11947784	0.031790403
C6	-1576.369855	0.28300701	0.130540797
C7	-1576.369938	0.23092368	0.142542078
C8	-1576.369133	0.73606923	0.060740714
C9	-1576.369044	0.79191762	0.055274122
C10	-1576.370306	0	0.210522097
C11	-1576.370273	0.02070783	0.203287651

Table S12. Calculated ECD data for **1a** in PCM (acetonitrile)

State	C1		C2		C3		C4	
	Excitation energies(eV)	Rotatory Strengths*	Excitation energies(eV)	Rotatory Strengths*	Excitation energies(eV)	Rotatory Strengths*	Excitation energies(eV)	Rotatory Strengths*
1	4.0915	-1.5385	4.0923	-3.1039	4.0927	-3.1232	4.0919	-0.5203
2	4.4354	-16.5781	4.4334	-12.7353	4.4343	-13.3108	4.4358	-15.737
3	4.8345	53.7981	4.8354	93.4621	4.8404	86.6287	4.8396	55.4791
4	5.1532	-3.4616	5.1512	-2.1455	5.1509	-1.2867	5.1533	-2.4993
5	5.1892	4.4017	5.1737	9.6844	5.1737	9.8081	5.1916	5.0709
6	5.3021	18.0378	5.2983	35.3054	5.2991	33.1908	5.3016	8.2064
7	5.3596	137.8147	5.353	53.5263	5.3599	37.1048	5.369	154.6956
8	5.4826	-128.3308	5.4815	153.2529	5.4835	173.9194	5.4864	-147.3244
9	5.6978	30.4734	5.7043	55.4934	5.7061	78.721	5.6993	44.7001
10	5.7238	-52.7886	5.7176	-143.2772	5.7166	-168.932	5.7253	-56.5473
11	5.8606	14.874	5.858	-1.2454	5.865	-2.8901	5.8666	28.3229
12	6.0475	-37.1153	6.0408	45.2323	6.0452	45.7462	6.0459	-76.7824
13	6.1675	21.8355	6.1533	18.9055	6.1602	22.981	6.1731	2.0262
14	6.2249	188.8616	6.2297	331.5164	6.2333	341.3656	6.2295	133.8002
15	6.2773	-57.4899	6.2976	-104.0719	6.3019	-112.767	6.2806	-27.6343
16	6.3432	-333.8968	6.331	-121.5043	6.3352	-147.38	6.3484	-298.6678
17	6.3851	85.762	6.4187	163.4995	6.423	146.5867	6.378	144.7508
18	6.4272	-54.9288	6.4298	-284.977	6.4316	-236.892	6.425	25.5331
19	6.4807	54.9278	6.4502	-49.4182	6.4574	-62.3849	6.4875	145.4138
20	6.5025	136.3953	6.5001	108.9017	6.5075	96.8194	6.5233	-22.7732
21	6.5322	-16.9786	6.5134	-24.9772	6.5232	-22.925	6.5537	16.7281
22	6.7217	-81.7126	6.6888	-141.4948	6.6914	-135.833	6.7156	-119.506
23	6.8246	-3.019	6.8253	2.7951	6.8307	1.9729	6.8255	1.5233
24	6.8864	46.5142	6.8616	-37.4693	6.8667	-36.4911	6.8665	20.3054
25	6.9348	-56.9547	6.9375	-40.2693	6.9378	-40.9461	6.9343	-55.1902
26	6.966	34.1833	6.9574	20.495	6.9579	20.7265	6.9692	23.9623
27	6.9798	-20.4109	7.0013	-19.4456	7.0044	-21.4934	6.9808	-13.4206
28	7.0343	4.9686	7.0361	-4.2731	7.0369	-3.3181	7.0395	2.3689
29	7.0667	8.3627	7.0522	11.5941	7.0573	16.1006	7.0693	-17.7001
30	7.0784	10.6769	7.0806	3.5216	7.0836	6.4488	7.0807	4.6775
State	C5		C6		C7		C8	
	Excitation energies(eV)	Rotatory Strengths*	Excitation energies(eV)	Rotatory Strengths*	Excitation energies(eV)	Rotatory Strengths*	Excitation energies(eV)	Rotatory Strengths*
1	4.0848	23.5945	4.0851	22.7967	4.0848	22.8677	4.0844	25.1991
2	4.533	-9.6098	4.5348	-11.6488	4.5348	-12.4499	4.5335	-8.3142
3	4.8121	-1.3875	4.81	37.8924	4.8159	25.9525	4.8161	15.6902
4	5.0584	-37.6282	5.063	-8.164	5.0729	-17.2985	5.0698	-21.5812

5	5.1889	5.9786	5.1888	4.5888	5.1888	4.6168	5.1888	6.0939
6	5.324	10.9844	5.3258	50.7271	5.3261	43.4012	5.3228	15.2268
7	5.3581	42.1637	5.3583	46.5975	5.3585	28.8628	5.3581	34.5536
8	5.4863	-129.7009	5.4897	48.9202	5.5104	67.0804	5.5068	-82.1067
9	5.5421	7.7399	5.5451	-8.7974	5.5453	-18.1039	5.5419	-2.083
10	5.7904	-13.3515	5.7903	-12.3013	5.7904	-11.3727	5.7902	-12.5235
11	6.0775	-77.3565	6.0777	-83.9464	6.0815	-64	6.0809	-80.5922
12	6.1047	55.6308	6.1038	80.592	6.1027	93.0273	6.1034	45.0133
13	6.2985	-38.6447	6.297	-22.39	6.3025	-24.6072	6.3034	39.1149
14	6.3131	-158.905	6.313	-20.8145	6.3183	-43.9463	6.3191	-186.809
15	6.4144	-133.4872	6.4064	-127.8528	6.3979	-100.476	6.4146	-104.975
16	6.443	227.8874	6.42	-124.0851	6.4212	-129.482	6.4341	70.3589
17	6.453	-69.1878	6.4495	36.7388	6.4493	-15.108	6.4493	61.6388
18	6.5193	165.3658	6.5277	117.216	6.5229	116.3311	6.5163	170.2942
19	6.6337	58.9543	6.6787	33.4642	6.6697	40.7743	6.6261	60.3156
20	6.7406	6.8175	6.7381	-28.7887	6.7452	-8.3972	6.7454	-12.1398
21	6.7488	69.583	6.7536	-4.7449	6.7809	-7.292	6.7778	88.4869
22	6.8227	-36.178	6.8229	87.7724	6.8151	86.6988	6.8152	-95.788
23	6.9292	-3.4544	6.9268	-0.0737	6.9291	0.3049	6.9328	-5.1078
24	6.9482	-0.6536	6.9543	-6.6219	6.9667	-6.9928	6.9588	-0.719
25	6.975	-54.7188	6.9759	-55.011	6.9758	-49.984	6.9746	-53.0338
26	7.1205	44.1397	7.1162	44.2614	7.1219	66.572	7.1296	-9.9861
27	7.1427	-9.2554	7.1405	9.422	7.1427	37.0258	7.1466	-29.5972
28	7.2197	10.1179	7.2252	-6.8963	7.2252	-6.5433	7.219	10.81
29	7.2642	39.5122	7.261	-34.5382	7.2644	-23.794	7.2696	33.3319
30	7.322	12.0441	7.3262	-17.9735	7.3276	-18.7703	7.3194	20.8814

State	C9		C10		C11	
	Excitation energies(eV)	Rotatory Strengths*	Excitation energies(eV)	Rotatory Strengths*	Excitation energies(eV)	Rotatory Strengths*
1	4.0844	24.0096	4.0841	25.251	4.0888	2.0868
2	4.5339	-9.4403	4.5336	-8.6275	4.4545	-13.6654
3	4.814	1.7084	4.8106	11.9336	4.872	11.7642
4	5.0641	-35.7391	5.0598	-25.1212	5.1353	2.2038
5	5.1889	5.9117	5.1889	5.939	5.1705	15.5782
6	5.3234	15.2672	5.3225	15.5277	5.3149	0.1167
7	5.3583	38.1827	5.3578	38.9701	5.4482	64.0269
8	5.4976	-122.784	5.4916	-110.173	5.531	-50.9819
9	5.5432	1.3681	5.5427	11.6511	5.6918	57.7791
10	5.7904	-13.4886	5.7905	-12.8529	5.7249	3.2878
11	6.0817	-72.2347	6.0787	-83.2625	5.8265	-19.2047
12	6.1094	51.7527	6.1032	43.4062	6.0777	-44.5291
13	6.3017	-81.1312	6.3014	150.6083	6.1041	-13.0692
14	6.3189	-109.109	6.3174	-312.182	6.2444	-117.142

15	6.4156	-129.246	6.4148	-74.7862	6.3331	307.2264
16	6.4441	251.0435	6.439	109.7457	6.3926	344.455
17	6.4542	-91.5705	6.4499	3.6297	6.4267	4.2213
18	6.5242	164.6088	6.5212	181.4572	6.4456	-99.2268
19	6.6355	62.3925	6.6277	57.8065	6.4743	-127.712
20	6.7448	-11.4662	6.7427	-13.4153	6.4833	-35.9977
21	6.781	105.908	6.7877	50.2674	6.5338	101.9165
22	6.8534	-57.4813	6.8618	-49.6786	6.7127	-243.186
23	6.9364	-3.5576	6.9358	-1.8764	6.7562	-1.7355
24	6.9574	-1.3464	6.9504	-2.0511	6.7967	-37.8164
25	6.9754	-53.9282	6.9748	-54.394	6.9297	-19.2196
26	7.1264	45.0557	7.1322	2.4714	6.9481	5.1825
27	7.1458	-6.6909	7.1505	-34.9156	6.993	28.5313
28	7.2191	11.369	7.2192	11.877	7.0324	-11.4928
29	7.2688	40.9707	7.2657	31.7637	7.0546	-11.7473
30	7.3225	13.71	7.3242	13.6419	7.0916	-16.6202
